# Exposures and potential health implications of contaminant mixtures in linked source water, finished drinking water, and tapwater from public-supply drinking water systems in Minneapolis/St. Paul area, USA†

**DOI:** 10.1039/d3ew00066d

**Published:** 2023-05-11

**Authors:** Kelly L. Smalling, Paul M. Bradley, Kristin M. Romanok, Sarah M. Elliot, Jane de Lambert, Michael J. Focazio, Stephanie E. Gordon, James L. Gray, Leslie K. Kanagy, Michelle L. Hladik, Keith A. Loftin, R. Blaine McCleskey, Elizabeth K. Medlock-Kakaley, Mary C. Cardon, Nicola Evans, Christopher P. Weis

**Affiliations:** a U.S. Geological Survey, Lawrenceville, NJ, USA; b U.S. Geological Survey, Columbia, SC, USA; c U.S. Geological Survey, Mounds View, MN, USA; d Minnesota Department of Health, Saint Paul, MN, USA; e U.S. Geological Survey, Reston, VA, USA; f U.S. Geological Survey, Kearneysville, WV, USA; g U.S. Geological Survey, Lakewood, CO, USA; h U.S. Geological Survey, Sacramento, CA, USA; i U.S. Geological Survey, Lawrence, KS, USA; j U.S. Geological Survey, Boulder, CO, USA; k U.S. Environmental Protection Agency, Durham, NC, USA; l U.S. National Institute of Environmental Health Sciences/NIH, Bethesda, MD, USA

## Abstract

Continued improvements in drinking-water quality characterization and treatment/distribution infrastructure are required to address the expanding number of documented environmental contaminants. To better understand the variability in contaminant exposures from the drinking water resource (surface and groundwater), through the distribution process, to the point-of-use (tapwater), in 2019 a synoptic assessment of broad chemical exposures was conducted in system-specific source waters, finished drinking water and service-area tapwater from 10 drinking water treatment facilities in the greater Minneapolis/St. Paul area of Minnesota, United States. Source water, finished water (collected pre-distribution in the treatment facility), and tapwater samples were analyzed for 465 unique organic compounds, 34 inorganic constituents, and 3 field parameters as well as *in vitro* estrogen, androgen, and glucocorticoid bioactivities. Mixtures of organic and inorganic contaminants were prevalent in source water, finished water, and tapwater samples, indicating the continued need for broad assessments of mixed contaminant exposures to characterize potential drinking-water human health outcomes. Contaminant concentrations were similar among drinking water sources and no exceedances of Environmental Protection Agency maximum contaminant level(s) (MCL) were observed in any treated sample (finished water or tapwater) in this study. No treated sample contained estrogenic, androgenic, or glucocorticoid activity at concentrations that may cause adverse human health effects. However, there were multiple exceedances of non-enforceable MCL goal(s) (MCLG), and other health advisories combined with frequent exceedances of benchmark-based hazard indices in both finished water and tapwater samples. These results indicate that exposure to contaminant mixtures is a potential public health concern underscoring our continued efforts to assess contaminant mixture exposures at the drinking-water point of consumption using a broad analytical scope.

## 1. Introduction

Compliance monitoring, regulation, and treatment of public supply drinking water in the United States (US) and elsewhere provide human health benefits,^1^ but continued improvements in both the treatment processes (*e.g.*, contaminant removal, disinfection) and infrastructure (*e.g.*, aging pipes, premise plumbing) are needed to address the expanding number of measured environmental contaminants.^2,3^ In recent years, the public has become more aware of the presence of contaminant mixtures at trace levels in the environment which can potentially increase household exposures (*e.g.*, through drinking water) and ultimately affect human health.^4,5^ In the US, the Safe Drinking Water Act (SDWA) requires drinking-water treatment facilities to routinely monitor chemicals regulated by the U.S. Environmental Protection Agency (EPA) prior to distribution.^6,7^ Few constituents (*e.g.*, lead (Pb) and copper (Cu)^8,9^) and residual disinfectants^10^ are routinely monitored after distribution and only at select point-of-use (tapwater) locations within the distribution pipeline. However, comprehensive assessments of human exposures to both regulated and unregulated contaminants broadly across a public-supply service area, including at the consumer’s tap, is both impractical and an acknowledged public health data gap of concern globally.^11–13^

Organic and inorganic contaminant mixtures are prevalent in both surface^14,15^ and groundwater drinking-water sources.^16,17^ As analytical methods become more sensitive/robust, a greater number of unregulated contaminant mixtures are being assessed more broadly, many of which break through the existing treatment processes and are observed in both finished drinking water^2^ and tapwater samples.^14,18,19^ Current research has focused on informing the public health data gap on exposures to a wide range of regulated and unregulated contaminants at the point-of-use (*i.e.*, homes, workplaces, and schools).^14,18,19^ Specifically for unregulated contaminants and mixtures of organic and inorganic contaminants, most studies to date have addressed source water quality and human exposure through drinking water separately, largely ignoring treatment and distribution-system factors (*e.g.*, aging infrastructure, premise plumbing, disinfection) that contribute to changes in contaminant exposures from the treatment plant to the tapwater point of exposure.

The U.S. Geological Survey (USGS) routinely collaborates with the EPA, National Cancer Institute (NCI), National Institute of Allergy and Infectious Disease (NIAID), National Institute of Environmental Health Science (NIEHS), Tribal Nations, universities, water utilities, communities, and others to inform exposure to a wide range of contaminants in drinking water at the point-of-use in studies across the US.^14,18–20^ Drinking water research at the USGS is national in scope but conducted modularly with spatially specific pilot studies designed to address community questions and priorities. For this reason, collection protocols, sampling personnel, targeted analytical methods/laboratories and quality assurance procedures are maintained across study areas to ensure comparability. Studies conducted to date have assessed contaminant mixtures in both public and private supply in a range of socioeconomic and source water vulnerability settings across the US.

To better understand how contaminant mixtures change as they move from drinking water resources (surface and groundwater), through the distribution process and to the tap, in 2019, the USGS, EPA, NIEHS and Minnesota Department of Health (MDH) conducted a synoptic assessment of broad chemical exposures in system-specific source waters, finished drinking water (hereafter, finished water) and service-area tapwater (hereafter, tapwater) samples corresponding to 10 drinking water treatment facilities and service areas. Sample pairing supported an initial assessment of infrastructure-associated exposure variability between the untreated intake (source), finished water (pre-distribution within the treatment plant) and the end-user tap (in the distribution pipeline).

## 2. Methods

### 2.1. Site selection and sample collection

For this synoptic assessment, source water samples representing three surface water and nine groundwater sources, were collected from 10 drinking water treatment facilities in the greater Minneapolis/St Paul area of Minnesota, US (Table S1†). We also collected samples from 10 finished water (collected in the drinking water treatment facility prior to distribution) and 17 service-area tapwater locations representing all 10-drinking water treatment distribution pipelines. Samples were collected one time in August 2019 with sample times varying throughout the day and without precleaning, screen removal or flushing of the sample tap and not comparable with the lead/copper rule sampling for compliance monitoring.^8^ Eight of the ten drinking water treatment facilities employed conventional treatment (flocculation, sedimentation, filtration) with chloride disinfection (*e.g.*, chlorine, chloramine, or chlorine dioxide). Two drinking water facilities employed no treatment. The data to support the findings and conclusions of this study are available from Romanok *et al.*^21^

### 2.2. Analytical methods and quality assurance

Tapwater samples were analyzed by the USGS for 465 unique organic compounds using six targeted methods,^22–27^ 34 inorganic constituents using three targeted methods;^28–30^ three field parameters (pH, temperature, specific conductance) and alkalinity^31^ as discussed in detail previously.^14,18,20,32^ Organic analytes included cyanotoxins, disinfection byproducts (DBP), pesticides, per/polyfluoroalkyl substances (PFAS), volatile organic compounds (VOC), and pharmaceuticals; additional method details are in the ESI† (Table S2). All samples for pharmaceuticals and pesticides were syringe filtered (0.7 μm nominal pore size, glass fiber) in the field. Bottles for pharmaceutical and VOC analysis were pretreated with ascorbic acid to neutralize chlorine/chloramine. Detailed information on analytes and detection limits for each of the methods are available in Romanok *et al.*^21^ and Table S2.†

Tapwater samples were also analyzed for *in vitro* estrogen (ER), androgen (AR), and glucocorticoid (GR) bioactivities by EPA using the T47D-kBluc cell line (American Type Cell Culture, Manassas, Virginia; ATCC CRL-2865; human estrogen receptor α/β) and the CV1 cell line (ATCC CCL-70) transduced (adenovirus) with the chimpanzee androgen receptor or the human glucocorticoid receptor as described in previously published methods.^33–37^ Briefly, cells were plated in 96-well luminometer plates and standards, controls, and samples were run in quadruplicate, and each sample screen was at least duplicated. After 24 hours cells were visually scored for cytotoxicity and any wells with cells exhibiting cytotoxic effects were excluded from subsequent analysis.^37,38^ Luminescence was quantified^39^ and endocrine-active samples were identified using a tiered screening process for tapwater.^40^ Biological equivalency values (BioEq) were calculated using an enrichment factor (EF) of 10 000 (ref. 41) and BioEq above the respective assay minimum detectable concentration (MDC; T47kBluc: 0.068 ng 17β-estradiol equivalents (Eq.) per L; CV1-chAR: 0.9 ng 4,5α-dihydrotestosterone Eq. L^−1^; and CV1-hGR: 5.41 ng dexamethasone Eq. L^−1^) were considered positive for endocrine activity.^39,40^

Quantitative (≥ limit of quantitation, ≥LOQ) and semi-quantitative (between LOQ and long-term method detection limit, MDL) results were treated as detections.^42–44^ Quality-assurance/quality-control included analyses of four field blanks, as well as two inorganic laboratory blanks, spikes, and stable isotope surrogates. Potassium (0.006 mg L^−1^) and sodium (0.054 mg L^−1^) were detected in inorganic laboratory blanks at concentrations less than 1% of those observed in tapwater samples; results were not censored (Table S5†). Among detected organics, 2-i-Pr-6-Me-4-pyrimidinol was detected in three of the four blanks in the concentration range observed in tapwater samples; results were censored at two times the maximum blank concentration (0.0053 μg L^−1^; Table S5†), resulting in removal from the dataset. The median surrogate recovery for organic analytes (Table S6†) was 103% (interquartile range 93–116%).

### 2.3. Statistical analysis and risk screening

Differences (centroids and dispersions) among sample types (source water, finished water, and tapwater) and source water types (surface water, groundwater) were assessed by one-way PERMANOVA (*n* = 9999 permutations) on Euclidean distance.^45^

A screening-level assessment^46,47^ of potential cumulative biological activity of mixed-organic contaminants in each tapwater sample was conducted as described previously.^14,48,49^ The ToxEval version 1.3.0^50^ was used to sum (non-interactive, concentration addition model, *e.g.* ref. 51–53) individual exposure activity ratios (EAR) from the toxicity ForeCaster (ToxCast, high-throughput screening data^54^) to estimate sample-specific cumulative EAR $\left(\sum \text{EAR}\right)$.^14,49^ EAR is the ratio of the detected concentration in the sample to the activity concentration at cutoff (ACC) obtained from the ToxCast database. The ACC estimates the point of departure concentration at which a defined threshold of response (cutoff) is achieved for a given biological activity and is less prone to violations of relative potency assumptions.^49^ ACC data in the ToxEval v1.3.0 employed in the present study were from the August 2022 invitroDBv3.5 release of the ToxCast database.^54^ Non-specific-endpoint, baseline, and unreliable response-curve assays were excluded.^14,49^ A ∑EAR=1 indicates a level that is expected to modulate a molecular target *in vitro* while a ∑EAR=0.001 is considered a precautionary screening level of interest. ∑EAR results and exclusions are summarized in Tables S8–S10.†

Because the ∑EAR approach was limited to organic compounds in ToxCast, an analogous human-health-based assessment^46,47,55^ of cumulative organic and inorganic contaminant risk was also conducted to sum the toxicity quotient (TQ; ratio of detected concentration to corresponding health based benchmark) of individual detections to estimate sample-specific cumulative TQ $\left(\sum \text{TQ}\right)$.^56^ A precautionary screening-level approach was employed based on the most protective human-health benchmark (*i.e.*, lowest benchmark concentration) among MCLG,^6,57^ WHO guideline values (GV) and provisional GV (pGV),^58^ USGS health-based screening level (HBSL^59^), and state drinking-water MCL or health advisories (DWHA). For the ∑TQ assessment, MCLG values of zero (*i.e.*, no identified safe-exposure level for sensitive sub-populations, including infants, children, the elderly, and those with compromised immune systems and chronic diseases^6,60^) were set to the respective method reporting limit, except for Pb, which was set to 1 μg L^−1^ as suggested by the American Academy of Pediatrics.^61^ Due to the inclusion of a margin of safety in health benchmarks, a ∑TQ=1 indicates a high probability of risk while a MATH ∑TQ<0.1 indicates no risk. ∑TQ results and respective health-based benchmarks are summarized in Tables S11 and S12.† Screening assessments were conducted in the program R version 3.6.1.^62^

## 3. Results and discussion

### 3.1. Organic and inorganic constituents in source water, finished water and tapwater

**Fig. 1:**
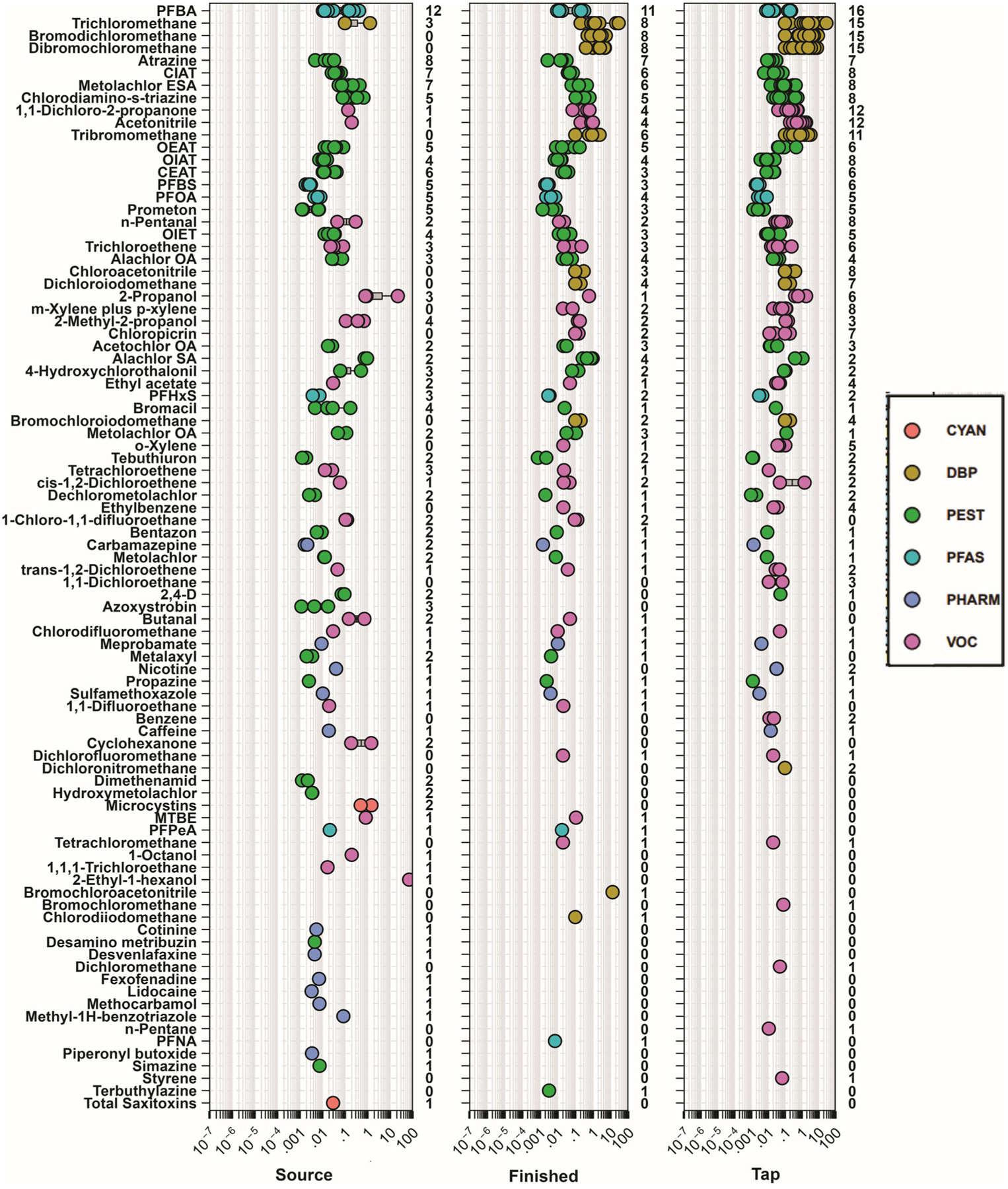
Concentrations (circles, μg L^−1^) and number of sites (right axes) for 89 organic analytes detected in intake source water samples from three surface water and nine groundwater sources (left plot, 12 total samples), samples from water-filtration-plant pre-distribution (finished water, center plot, 10 total samples) and service-area tapwater business locations (right plot, 17 total samples) during 2019, for 10 drinking water treatment plants in the greater Minneapolis/St. Paul area of Minnesota, US. Circles are data for individual samples. Boxes, centerlines, and whiskers indicate interquartile range, median, and 5th and 95th percentiles, respectively. Individual compounds are organized in descending order based on detection frequencies (left axis) in finished water samples (center plot).

**Fig. 2:**
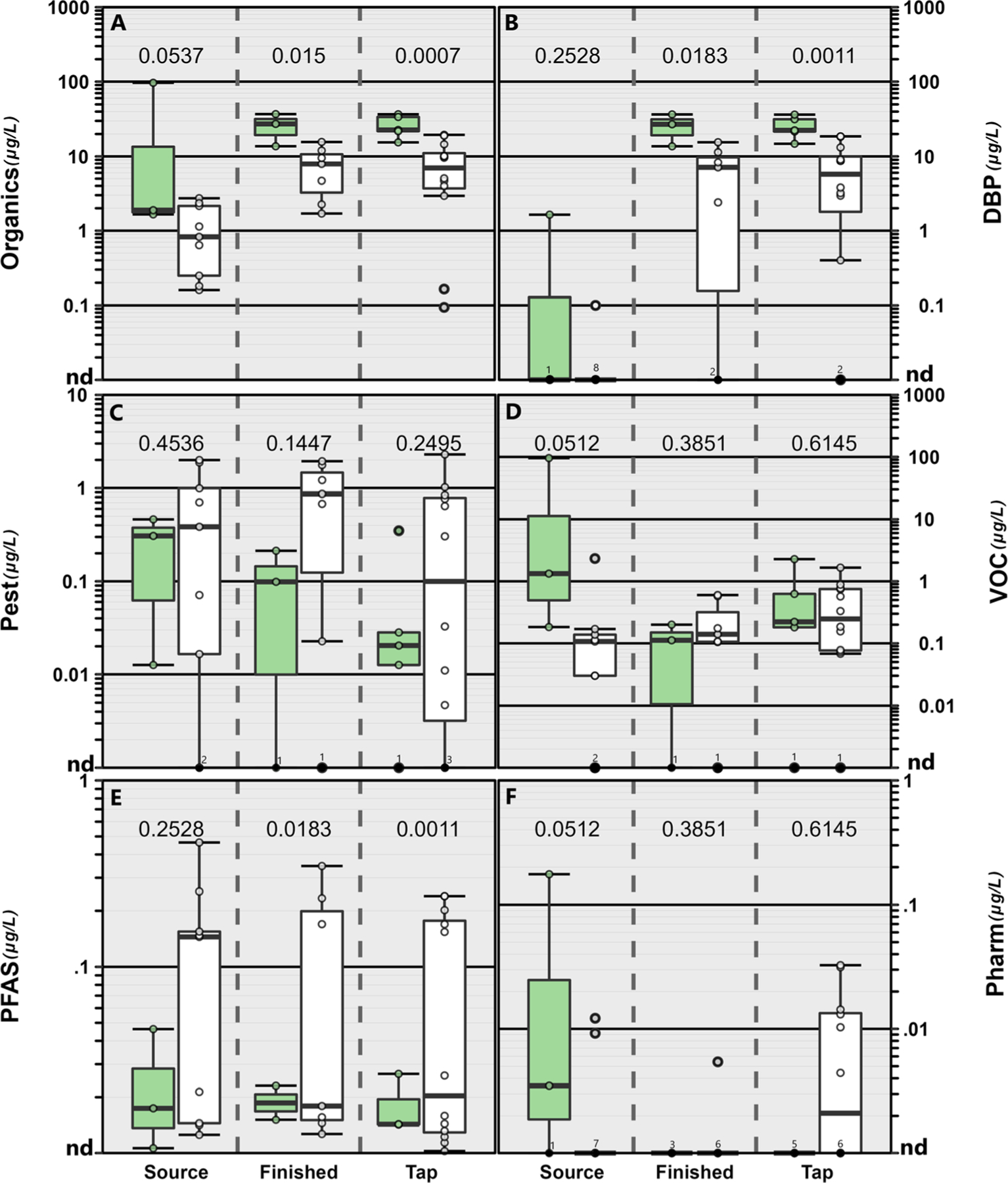
Concentrations (μg L^−1^) of cumulative organics (A), disinfection byproducts (DBP; B), pesticides (Pest; C), volatile organic compounds (VOC; D), per-and polyfluoroalkyl substances (PFAS; E) and pharmaceuticals (Pharm; F) in source water, finished water and tapwater samples collected from the greater Minneapolis/St Paul area, Minnesota in 2019. Samples originating from surface water sources are shown in the green boxes and those originating from groundwater sources are shown in the white boxes. Circles (●) are data for individual samples. Boxes, centerlines, and whiskers indicate interquartile range, median, and 5th and 95th percentiles, respectively. Above each boxplot pair, the permuted probability that the centroids and dispersions are the same is estimated to be *p* < 0.05 (One-Way PERMANOVA; 9999 permutations; *p* < 0.05).

Regulated and unregulated organic and inorganic contaminants were observed in source water, finished water, and tapwater samples collected in Minnesota (Fig. 1; Tables S3 and S4†), consistent with other studies in the US^2,14,15,19,20,63–65^ and globally.^66–70^ Of the 465 unique organic contaminants assessed during the study, 89 (19%) were detected at least once and 81% were never detected. The PFAS, perfluorobutanoate (PFBA) was the only organic compound observed in 100% of the samples, while three DBPs, the herbicide atrazine, and a degradate of the herbicide metolachlor (metolachlor sulfonic acid) were observed in >50% of the samples (Fig. 1; Table S3†). One surface-water source sample had the highest cumulative organic contaminant concentration observed (97.0 μg L^−1^) with concentrations close to an order of magnitude higher than any other source water sample collected (Fig. 2). Cumulative concentrations in this sample were dominated by two VOCs (2-ethyl-1-hexanol and isopropyl alcohol) which were not detected in the respective finished water and tapwater samples, indicating loss/removal during treatment (Fig. 1). Cyanotoxins including saxitoxins and microcystins were observed in three surface-water source samples at concentrations ranging from 0.05–1.5 μg L^−1^ with no detections in any finished water or tapwater sample.

Concentrations/detections differed among sample types (source water, finished water, tapwater) due to environmental and infrastructure factors. Concentrations of pesticides, VOCs, and pharmaceuticals were similar among sample type (Fig. 2). Due to the variability in PFAS in sources originating from groundwater in our study, we observed no differences in PFAS concentrations between surface and groundwater sources (*p* = 0.2422); however, concentrations were higher in finished water (*p* = 0.0168) and tapwater (*p* = 0.0006) samples originating from groundwater sources (Fig. 2). PFAS has been observed frequently in drinking water resources throughout the US^71^ particularly in groundwater sources.^72,73^ A greater number of organic compounds (excluding DBPs) were often observed in surface source waters compared to finished water and tapwater samples (Table S3†), consistent with other studies^2,15^ and indicative of some removal during treatment. However, in groundwater sources the number of compounds observed was similar among sample types.

As expected, observed differences in organic contaminants among source and drinking waters were attributable to concentrations of DBPs in both finished water and tapwater samples (Fig. 1 and 2). Chlorine-based disinfection (*e.g.*, chlorination, chloramination, or chlorine dioxide) is common in US public-supply drinking water^74,75^ to kill harmful microorganisms thereby eliminating drinking-water specific epidemics (*e.g.*, cholera).^76,77^ Thus, DBPs were detected in 8 of the 10 finished water and in 15 of the 17 tapwater samples, comprising 51–99% and 13–99% of the mass concentration of organics detected in finished water and tapwater samples, respectively. Conversely, DBPs were not detected in the tapwater samples from the two facilities (005, 015) that did not employ treatment (Table S3†). Spatiotemporal variability in DBP concentrations and profiles between treatment-plant finished water and tapwater have been studied extensively in previous studies.^78,79^ DBP formation and cumulative concentrations varied by drinking water source; finished water and tapwater samples originating from groundwater sources had lower cumulative DBP concentrations (median 7.1 μg L^−1^; IQR: 1.4–10.2 μg L^−1^) compared to those originating from surface water sources (median: 24.7 μg L^−1^; IQR: 19.9–32.8 μg L^−1^; Fig. 2). These results are not surprising as DBP formation is driven by the types and concentrations of natural organic matter in drinking water sources.^80^ Groundwater-sourced tapwater typically has lower concentrations of source-water organic matter and, thus DBPs, compared to surface-water sourced tapwater.^74,81^ Of the DBPs detected, trichloromethane (chloroform), a byproduct of chlorine-based treatment but also reported to occur naturally at low levels,^82,83^ was observed most frequently (67% of the samples) including in three source water samples (two groundwater and one surface-water sample; Fig. 1).

**Fig. 3:**
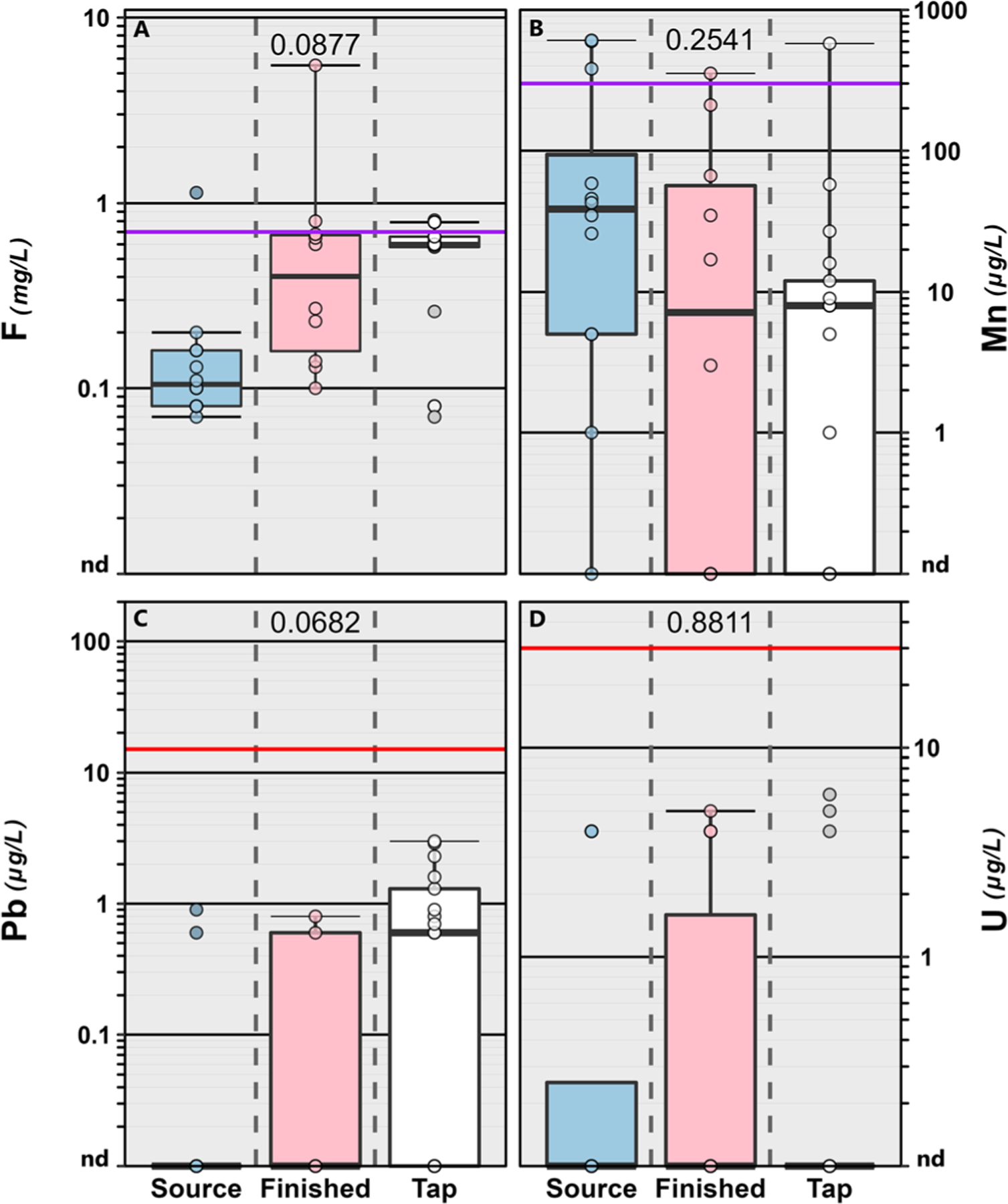
Concentrations (mg L^−1^) of fluoride (F; A) and concentrations (μg L^−1^) of manganese (Mn; B), lead (Pb; C) and uranium (U; D) in source water (blue shaded boxes), finished water (pink shaded boxes) and service-area tapwater (white boxes) samples collected from the greater Minneapolis/St. Paul area, Minnesota in 2019. Seven (2 source water and 5 finished water) samples were removed from Pb plot (bottom left) because they were collected from non-potable faucets with brass taps. For all Pb data see Fig. S1.† Circles (●) are data for individual samples. Boxes, centerlines, and whiskers indicate interquartile range, median, and 5th and 95th percentiles, respectively. For each element, colored lines indicate health-based National Primary Drinking Water Regulation Maximum Contaminant Level (MCL: U) and non-health-based National Primary Drinking Water Regulation Action Level (Pb) or non-enforceable EPA Drinking Water Health Advisory (Mn) or the US Public Health Service optimum (F). The MCL Goals (MCLGs) for Pb and U are zero. The numbers in each panel indicate the permuted probability that the centroids and dispersions are the same among sample type is estimated to be *p* < 0.05 (One-Way PERMANOVA; 9999 permutations; *p* < 0.05).

Conversely, 31 (94%) of the 33 inorganics assessed were detected at least once (Table S4†). No differences among sample types were observed for field parameters including pH (*p* = 0.861) and specific conductance (*p* = 0.565). Copper (Cu) which was detected in 95% of the samples (all but two) with a maximum concentration of 1040 μg L^−1^ (median: 32.0 μg L^−1^; IQR: 6.00–94.0 μg L^−1^). Lead (Pb) was also detected in 54% of the samples with a maximum of 78.1 μg L^−1^ (finished water sample) and a median of 1.3 μg L^−1^ (IQR: 0.80–3.0 μg L^−1^). Detections of both Cu and Pb in drinking water are more likely associated with distribution system infrastructure (*e.g.*, legacy pipe materials and Cu fittings) and premise-plumbing materials.^84,85^ In our study, elevated Pb concentrations were observed at treatment-plant sampling points (source water and finished water) with non-potable brass faucet taps (Fig. 3 and S1†) and not at tapwater sampling points with potable faucets, suggesting that the non-potable brass taps were a source of elevated Pb and not representative of the drinking-water supply. These seven data points were removed from further analyses (Fig. S1†), particularly those associated with effects-based screening (see section 3.3). The frequent detections of contaminants from infrastructure and plumbing (Cu, Pb) as well as those produced (DBPs) or not removed during the treatment (*e.g.*, pesticides, VOC) reinforces the need for continued assessment of contaminant mixtures directly at the tap using an array of analytical methods to adequately represent the complexity of these mixtures and human exposures in public supply drinking water where compliance monitoring does not always reflect residential exposures within the distribution system.

### 3.2. *In vitro* estrogenic, androgenic, and glucocorticoid activity

Estrogenic activity was detected above bioassay-specific MDC (Table S7†) in six samples ranging from 0.028–0.868 ng E2Eq L^−1^ (median: 0.072 ng E2Eq L^−1^). Samples with activity included three surface-water sources (median: 0.112 ng E2Eq L^−1^), one groundwater source (0.028 ng E2Eq L^−1^), one finished water (0.210 ng E2Eq L^−1^), and one tapwater (0.028 ng E2Eq L^−1^) location. No samples produced androgenic or glucocorticoid activity above respective method MDC. No sample exceeded any effects-based trigger values (indicative of adverse health effects) developed for bioassays that quantify the same molecular endpoints.^86^

### 3.3. Individual contaminant risk-based screening

No exceedances of any available EPA promulgated MCL or action level (AL) were observed in any tapwater sample collected during the study (Fig. 3; Tables S3 and S4†). EPA also sets non-enforceable MCLG values for public supply drinking water that are established without cost and treatment technology considerations and are based on a margin of exposure to provide a safety threshold for sensitive subpopulations including infants, children, the elderly and those with compromised immune systems or chronic illnesses.^6,60^

Established MCLG or health-based screening values are available for 52% (47 of 89) of the organic compounds detected (Table S12†). Twenty-eight of the detected organic compounds have health-based screening values and none were exceeded in any finished water or tapwater sample collected (Table S12†). However, nineteen of the detected organic compounds have MCLG values and eight were exceeded at least once including three DBPs, four VOCs and 1 PFAS (Table S3†). The four VOCs (benzene, tetrachloroethane, tetrachloromethane, and trichloroethane) are known carcinogens and consequently have MCLG of zero.^6,57^ Benzene was observed only in two tapwater samples; tetrachloromethane was observed in one finished water and one tapwater sample; and tetrachloroethane and trichloroethane were observed in both finished water and tapwater samples (tetrachloroethane: one finished water, two tapwater; trichloroethane: one finished water, six tapwater samples) receiving water from both surface and groundwater supplies. Consistent with other studies, VOCs are observed frequently at low levels in drinking water supplies at concentrations that could have adverse health effects.^87,88^

The DBPs, bromodichloromethane, dibromochloromethane, and tribromomethane were observed most frequently and in 80%, 80% and 60% of the finished water and 88%, 88% and 65% of the tapwater samples, respectively. Trichloromethane was also observed in 80% of the finished water, 88% of the tapwater and 25% of the source water samples while dichloromethane was only observed in a single tapwater sample. Iodinated, haloacetonitriles and halonitromethanes are rarely monitored routinely and are not currently regulated but are considered more toxic than regulated trihalomethanes and haloacetic acids;^89–91^ iodinated haloacetonitriles, and halonitromethanes were detected in several samples in this study with concentrations ranging from 0.012–13.0 μg L^−1^. The public-health benefits of disinfection as a means to prevent water-borne disease outbreaks and to control pathogen occurrence in drinking-water infrastructure has been well documented,^76,77^ however, the ubiquitous detection and subsequent health effects of regulated and unregulated DBPs are growing public health concerns.^89^ Continued monitoring of regulated, unregulated, and unknown DBPs is important to improve our understanding of the exposure and associated cumulative risk in public supply drinking water.^20,89,92^

We utilized targeted analysis of 32 PFAS in source water, finished water, and tapwater samples as fractional indicators of the presumptive 8000+ PFAS contaminant space.^93^ PFBA concentrations ranged from 0.008 to 0.425 μg L^−1^ (median: 0.016 μg L^−1^) and were below Minnesota’s non-enforceable health-based guidance level of 7 μg L^−1^.^94^ PFOA was detected in 42% (5/12) of source water samples, 40% (4/10) of the finished water samples and 29% (5/17) of tapwater samples with concentrations ranging from 0.002 to 0.008 μg L^−1^ (median of detected values = 0.0052 μg L^−1^). All tapwater samples with PFOA detections were below Minnesota’s health risk limit of 0.035 μg L^−1^.^95^ US State and Federal agencies continue to update drinking water regulations and health-based advisories for PFAS. More protective drinking water regulations and health-based advisories for PFAS are rapidly proliferating at the US state and federal levels^96^ due to their ubiquitous occurrence, persistence in the environment,^97^ widespread detection in drinking water resources,^14,19,20,71,98,99^ and documented health concerns.^96,100–103^ Newly proposed MCLs for PFOA (0.004 μg L^−1^) and PFOS (0.004 μg L^−1^) were released for public comment in March 2023 by EPA as part of the National Primary Drinking Water Standards Rule.^104^ PFOS was not detected in any sample, but proposed PFOA MCLs were exceeded in 28% of the samples collected including 5 source waters (all groundwater); 2 finished water; and 4 tapwater samples. A MCLG (level at which there is no known or anticipated adverse effect on the human ensuring an adequate margin of safety) of zero also was proposed for PFOS and PFOA;^104^ every detection of PFOA (14 of 39) was a *de facto* exceedance of the proposed MCLG. Further, to account for dose additive noncancer effects of PFBS, PFNA, PFHxS, GenX, EPA proposed an MCLG for the mixture of these four PFAS based on a hazard index approach.^105^ The proposed hazard index (HI) of 1 for the sum of PFBS + PFNA + PFHxS + GenX was only exceeded in 1 finished water sample. Detections of multiple PFAS compounds is consistent with other drinking water^71,98^ and tapwater studies^14,19,20^ in the US. Information generated herein and elsewhere indicates the need for further assessments of cumulative health risks of PFAS mixtures,^106^ and PFAS in combination with other organic and inorganic contaminants of concern.

Fifty-two percent (16 of 31) of the inorganics detected have either established MCLG values or state/federal health-based screening levels (Table S12†). Ten of the inorganics detected have established MCLG values and three were exceeded at least once (Table S4†) in finished water and tapwater. The MCLG for lead and uranium are zero and was exceeded (*i.e.*, detected) in 70% and 30% of the finished water and 59% and 18% of the tapwater, respectively, collected during the study (Table S4†). Arsenic was only detected in one groundwater source sample at 2 μg L^−1^ and was not observed above the detection limit in the corresponding finished water and tapwater samples. As noted previously, approximately half the MCLG exceedances in finished water samples were observed in locations with non-potable brass taps (Fig. 3 and S1†) and are not considered relevant in this study from a human exposure perspective. Arsenic and uranium occur naturally in the environment and often are not completely removed during public-supply drinking-water treatment processes.^107^ Adverse health effects associated with uranium and lead in drinking water have been well documented. Recent studies have linked drinking water uranium exposure to osteotoxicity^108^ and nephrotoxicity^109^ in humans. Drinking water lead exposure is of particular concern to formula-fed infants, children and pregnant or breast-feeding women.^61,110,111^ Health effects can include fetal death and reduce birth weights,^112^ cognitive impairment,^61,110^ cardiovascular diseases and mortality.^61,113^

Detected fluoride concentrations were well below the EPA MCL (4000 μg L^−1^)^6,57^ indicating little concern for toxic effects within this study. However, 80%, and 76% of the finished water and tapwater samples, respectively were below the US Public Health Service^114^ optimum level to prevent dental caries. Consistent with groundwater results,^115,116^ fluoride levels in source water samples were low (median: 0.105 mg L^−1^) indicating some supplementation during treatment.

EPA and USGS maintain a life-time drinking-water health advisory^117^ and a health based screening level,^59^ respectively, of 300 μg L^−1^ for manganese while the Minnesota Department of Health (MDH) maintains a health based screening value of 100 μg L^−1^ for infants.^94^ Concentrations of manganese exceeded the EPA/USGS and the MDH screening levels in 7% (2 of 27 samples) and 11% (3 of 27 samples) of the tapwater samples, respectively. Observed concentrations were above the screening levels more often in source water, compared to finished water or tapwater (Fig. 3). These results are consistent with drinking-water aquifer data from across the US, in which approximately 7% of the samples were above the guidance value for manganese.^118^ Due to potential cognitive and behavioral effects to children from manganese exposure at concentrations below 300 μg L^−1^ (ref. 119–121) there have been calls by public health communities to reevaluate the current drinking water standards as well as continued monitoring of drinking water supplies, especially those relying on groundwater sources.^122–124^

### 3.4. Effects-based screening assessments (∑EAR and ∑TQ) in source waters and drinking water

We used two bioactivity weighted approaches (∑EAR and ∑TQ) to screen all samples for cumulative exposure effects of potential human-health interest based on detected mixtures. Although source water samples are not typically assessed for potential human-health effects, we opted to include all samples collected during this study as a means of comparing values among source water, finished water and tapwater samples. The ∑EAR approach uses high throughput exposure-effects data from ToxCast to estimate cumulative activity of over 10 000 organics using molecular endpoints (*in vivo*), however, not all predicted molecular responses are necessarily adverse at the organismal level^18,49^ and may not accurately reflect apical human health endpoints.^49,125,126^ ∑EAR is often used as a precautionary screening tool but because it has no coverage of inorganic contaminants, the ∑TQ approach is also utilized as well.^18,127^ The ∑TQ approach assesses the effects of both organic and inorganic contaminant exposures, is targeted at apical human-health endpoints but is limited by existing health benchmarks.^18^ Both approaches have their advantages and disadvantages but together they provide a reasonable assessment of the potential human-health effects to mixed contaminant exposures assuming cumulative effects are reasonably approximated by concentration addition.^127,128^

**Fig. 4:**
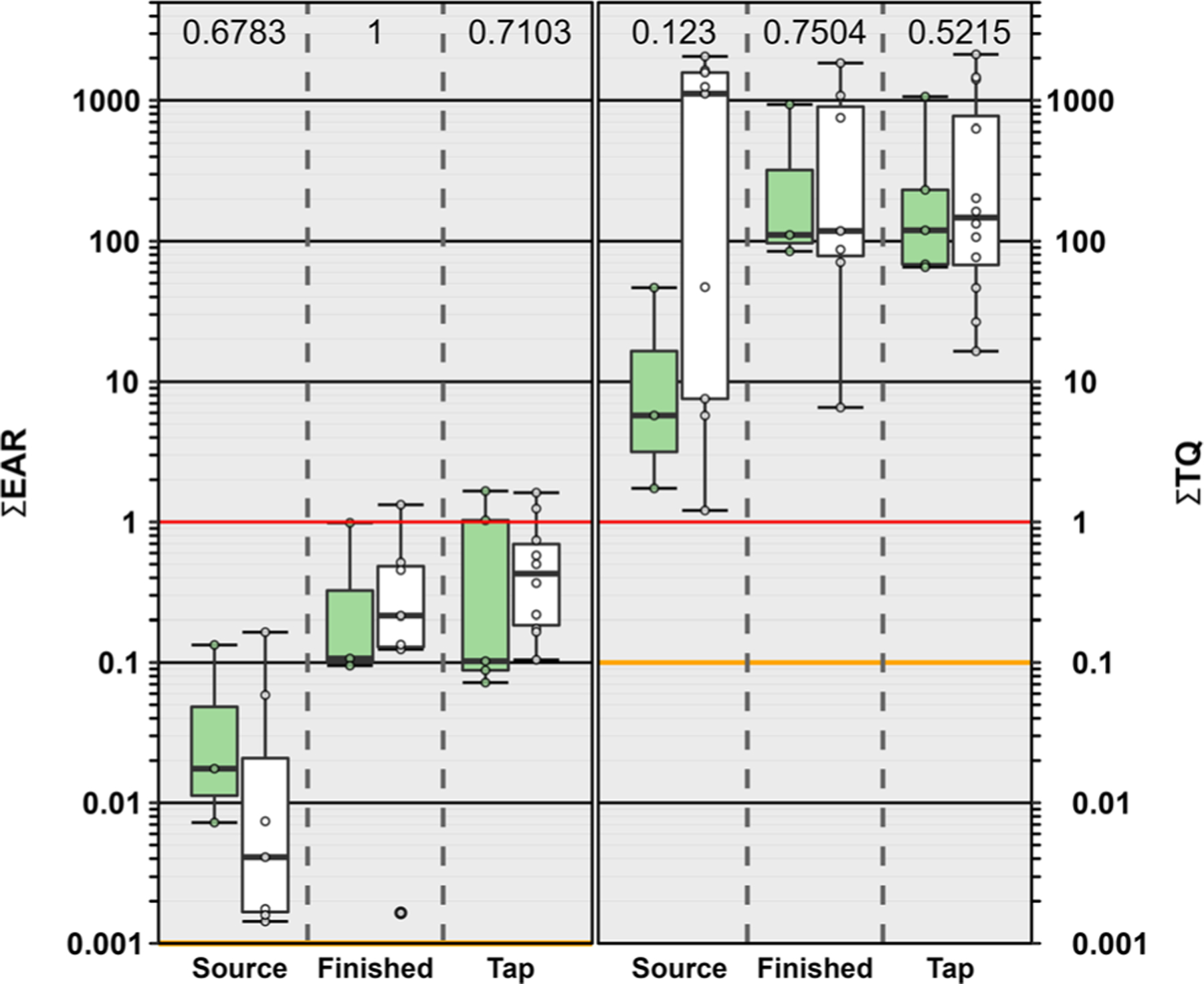
Left. Cumulative maximum exposure-activity ratios $\left(\sum \text{EAR}\right)$ across all assays for 44 analytes listed in ToxCast and detected in source water, finished water and tapwater samples. Solid red and yellow lines indicate concentrations shown to modulate effects *in vitro* and effects-screening-level thresholds (EAR = 1 and EAR = 0.001), respectively. Right. Human health benchmark cumulative toxicity quotient $\left(\sum \text{TQ}\right)$ for inorganic and organic analytes listed in Table S11† and detected in source water, finished water and tapwater samples. Solid red and yellow lines indicate benchmark equivalent concentrations and effects-screening-level threshold of concern (TQ = 1 and TQ = 0.1), respectively. Samples originating from surface water sources are shown in the green boxes and those originating from groundwater sources are shown in the white boxes. Circles (●) are data for individual samples. Boxes, centerlines, and whiskers indicate interquartile range, median, and 5th and 95th percentiles, respectively. Above each boxplot pair, the permuted probability that the centroids and dispersions are the same is estimated to be *p* < 0.05 (One-Way PERMANOVA; 9999 permutations; *p* < 0.05).

Of the 89 compounds detected in this study, 49% (44) had exact Chemical Abstract Services number matches in the ToxCast database. No differences were observed in ∑EAR values among source water, finished water, and tapwater samples (*p* = 0.097). We did observe higher ∑EAR in source waters originating from surface water compared to those originating from groundwater (*p* = 0.0143) and these differences were driven by VOCs and several pesticides. ∑EAR ranged from 0.00026–0.1645 (median: 0.0057; IQR: 0.0016–0.028) in source water, 0.002–1.33 (median: 0.174; IQR: 0.111–0.500) in finished water and 0.00002–1.66 (median: 0.218; IQR: 0.102–0.740) in tapwater (Fig. 4). A ∑EAR greater than 1 (solid red line, Fig. 4) indicates cumulative exposures at concentrations capable of modulating molecular endpoints *in vitro* while ∑EAR>0.1 indicate elevated probabilities of an effect and ∑EAR=0.001 is a considered precautionary level of potential concern (yellow line, Fig. 4) as described previously.^14^ Four tapwater samples and one finished water sample had ∑EAR>1 indicating a high probability of molecular effects which were attributable to the DBP, dibromochloromethane (Fig. S2†). Fifteen of the 39 samples had a ∑EAR>0.1 indicating elevated probabilities of effects driven primarily by dibromochloromethane in all but two source water samples where the MATH EAR was driven by VOCs (Table S9†). The precautionary screening level, ∑EAR=0.001, was exceeded in all but four samples in this study (two source water, and two tapwater samples; Table S9†). Currently our target analytical methods only capture a small proportion (465 analytes) versus the more than 100 000 commercial organic compounds in production,^129^ not including the unknown number of degradates^130,131^ and metabolites that could occur in the environment. For these reasons, a precautionary screening level of 0.001 was deemed acceptable for these and other investigations of potential effects.^14^ Our results indicate low potential effects when considering only the detected organic compounds with exact matches in ToxCast. However, the ∑EAR exceedances of 0.001 in 79% of the samples in our study demonstrate that more information is needed on cumulative effects of contaminant mixtures with an emphasis on unregulated, unmonitored, and unknown compounds in both source and drinking waters.

To estimate cumulative effects from exposures to both organic and inorganic contaminant mixtures, a benchmark based ∑TQ approach was used. All source water and public supply tapwater samples in this study exceeded both the ∑TQ=0.1, threshold of potential concern, and a ∑TQ=1, indicating a high probability of aggregated risk when considering exposures to both organic and inorganic contaminants (Fig. 4; Table S11†). Individual MATH ∑TQ comparisons indicated that potential exposure risk was dominated by PFAS (PFOA), DBPs (bromodichloromethane, tribromomethane), VOC (trichloroethane), cyanotoxin (microcystins) and regulated inorganics (fluoride, lead, manganese, uranium; Fig. S3, Table S11†). When detected, maximum TQ values for PFOA were an order of magnitude higher than those reported for bromodichloromethane, tribromomethane and lead (Fig. S3; Table S11†). ∑TQ values were lower in source waters derived from surface water compared to both finished water and tapwater samples (*p* = 0.0083; Fig. 4) but no differences were observed among groundwater source sample types. These apparent differences were due to the frequent detection of PFOA in samples sourced from groundwater compared to surface water.

## 4. Conclusion

Human-health risk from mixtures of regulated and unregulated contaminants in drinking water is a function of cumulative exposure whether additive, synergistic, or antagonistic and associated hazard or effect. This study provides a broad assessment of mixed organic and inorganic contaminant exposures in tapwater compared to traditional compliance monitoring of source water and finished drinking water to inform the importance of these exposures as drivers of human health outcomes. It is important to note that approximately 80% of the organic compounds analyzed in each sample were not detected indicating the quality of drinking water resources in Minnesota and the effectiveness of treatment. However, these results also demonstrate that human exposure to organic and inorganic contaminants not typically measured during routine compliance monitoring or at the point-of-use are ubiquitous in public supply tapwater throughout the study area. Contaminant concentrations were similar among drinking water sources and these sources were important drivers of organic and inorganic contaminant detections in finished water and tapwater. Further, these results suggest that an expanded analytical coverage of treated drinking water prior to distribution, including DBPs, PFAS, and pesticides may adequately predict tapwater exposures in Minneapolis/St. Paul area. However, concentrations of several contaminants including lead and infrequently detected organic contaminants indicate the continued characterization and monitoring of a broad suite of organic and inorganic contaminants at the tap to support public health agencies and decision makers.

Only one-third of the contaminants detected have established MCLs and no exceedances were observed in any finished water or tapwater sample in this study, indicating both compliance with existing regulations and effective treatment. However, multiple exceedances of EPA MCLG values and state advisories for inorganic (*e.g.*, lead, manganese and uranium) and organic (*e.g.*, bromodichloromethane, tribromomethane, PFOA) analytes combined with frequent exceedances of ∑TQ=1, indicated potential cumulative risk to vulnerable subpopulations and emphasized the continued need to support contaminant mixture exposure assessments at the tap using a range of analytical methods including nontarget analysis and other high throughput platforms (*e.g.*, effects-based monitoring) at various points in the distribution pipeline. Improved public communication and outreach of low level (sub-MCL) mixed contaminant exposures in public supply tapwater is needed. This information can be used to support consumer assessments of acceptable personal risk as well as potential point-of-use treatment decisions to minimize their exposure risk.

## Supplementary Material

Supplement1

## Water impact

Contaminant mixtures are present in drinking water resources in the US and globally. Currently, potential human exposures to contaminant mixtures in drinking water are assessed in the source-water or finished-water at the treatment facility, ignoring treatment and distribution-system changes contributing to actual exposures at the consumer tap. Point-of-use contaminant mixture exposures and associated human health risks are poorly understood.

## Footnotes

† Electronic supplementary information (ESI) available: Data discussed in this paper are summarized in ESI (Tables S1–S12) and in the USGS data release^21^ (https://doi.org/10.5066/P9YQ24QW). See DOI: https://doi.org/10.1039/d3ew00066d
