# Supplementary material for: Exposures and potential health implications of contaminant mixtures in linked source water, finished drinking water, and tapwater from public-supply drinking water systems in Minneapolis/St. Paul area, USA
Source: Environ Sci (Camb). Author manuscript; Available in PMC 2025 Jun 20. (PMC12180763; doi:10.1039/d3ew00066d)
Supplement: Supplement1 [file NIHMS2070936-supplement-Supplement1.pdf]

Electronic Supplementary Material (ESI) for Environmental Science: Water Research & Technology.
This journal is © The Royal Society of Chemistry 2023

Supplemental Information

Exposures and potential health implications of contaminant mixtures in linked source water, finished drinking water, and tapwater from public-supply drinking water systems in Minneapolis/St. Paul area, USA

Kelly L. Smalling[a*], Paul M. Bradley[b], Kristin M. Romanok[a], Sarah M. Elliot[c], Jane de Lambert[d], Michael J. Focazio[e], Stephanie E. Gordon[f], James L. Gray[g], Leslie K. Kanagy[g], Michelle L. Hladik[h], Keith A. Loftin[i], R. Blaine McCleskey[j], Elizabeth K. Medlock-Kakaley[k], Mary C. Cardon[k], Nicola Evans[k], Christopher P. Weis[l]

[a] U.S. Geological Survey, Lawrenceville, NJ, USA
[b] U.S. Geological Survey, Columbia, SC, USA
[c] U.S. Geological Survey, Mounds View, MN, USA
[d] Minnesota Department of Health, Saint Paul, MN, USA
[e] U.S. Geological Survey, Reston, VA, USA
[f] U.S. Geological Survey, Kearneysville, WV, USA
[g] U.S. Geological Survey, Lakewood, CO, USA
[h] U.S. Geological Survey, Sacramento, CA, USA
[i] U.S. Geological Survey, Lawrence, KS, USA
[j] U.S. Geological Survey, Boulder, CO, USA
[k] U.S. Environmental Protection Agency, Durham, NC, USA
[l] U.S. National Institute of Environmental Health Sciences/NIH, Bethesda, MD, USA

Corresponding author: Kelly L. Smalling, USGS, New Jersey Water Science Center, Lawrenceville, NJ 08628, ksmall@usgs.gov

The authors declare they have no actual or potential competing financial interests.

Data discussed in this paper are summarized in Supporting Information (Figures S1-3, Tables S1-S12) and in the USGS data release[28] (https://doi.org/10.5066/P9YQ24QW).

Contents: Cover Page, Table of contents, 3 Figures, 12 Tables

Page 4: Figure S1. Comparison of lead concentrations in source water (blue boxes), finished water (pink boxes) and service-area tapwater (white boxes) using all data including locations where samples were collected from potable and non-potable faucets (left) and potable faucets only (right).

Page 5: Figure S2. Exposure-activity ratios (EAR) and number of samples (right axes) for organic analytes (left axis, in order of decreasing total detections) detected in source water, finished water, nd service-area tapwater samples collected from Minnesota, 2019.

Page 6: Figure S3. Toxicity Quotients (TQ) and number of samples (right axes) for organic analytes (left axis, in order of decreasing total detections) detected in source water, finished water, and service-area tapwater samples collected from Minnesota, 2019.

Page 7: Table S1. Site information for samples collected as part of the U.S. Geological Survey (USGS) Ecosystems Mission Area, Environmental Health Infrastructure Project, Minnesota Tapwater Exposure study in, 2019.

Page 9: Table S2. Method information for compounds analyzed for the U.S. Geological Survey (USGS) Ecosystems Mission Area, Environmental Health Infrastructure Project, Minnesota Tapwater Exposure study, 2019.

Page 38: Table S3. Summary data for detected organic compounds, in micrograms per liter, analyzed in source water, finished water, and service-area tapwater samples collected for the U.S. Geological Survey (USGS) Ecosystems Mission Area Environmental Health Infrastructure Project, Minnesota Tapwater Exposure Study, 2019.

Page 40: Table S4. Subset of detected trace element and ion concentrations (in units shown) in source water, finished water, and service-area tapwater samples collected for the U.S. Geological Survey (USGS) Ecosystems Mission Area, Environmental Health Infrastructure Project, Minnesota Tapwater Exposure Study, 2019.

Page 42: Table S5. Quality assurance organic and inorganic analyte results (units listed) laboratory blanks analyzed at the U.S. Geological Survey, Redox Chemistry laboratory, Boulder, Colorado in bottled water samples collected for the USGS Ecosystems Mission Area, Environmental Health Infrastructure Project, Minnesota Tapwater Exposure Study, 2019.

Page 42: Table S6. Quality assurance surrogate recovery or internal dilution standard summary statistics (percent) for the U.S. Geological Survey (USGS) Ecosystems Mission Area, Environmental Health Infrastructure Project, Minnesota Tapwater Exposure Study, 2019.

Page 45: Table S7. Mammalian Bioactivities in ng equivalents per liter (ng L-1) of standard steroid hormone (E2, DHT, DEX) analyzed in source water, finished water, and service-area tapwater samples collected in Minnesota, 2019.

Page 46: Table S8. Compound: endpoint combinations excluded from ToxCast evaluation due to unreliable concentration-response relationship and resulting lack of confidence in activity concentration at cutoff (ACC).

Page 48: Table S9. Site-specific Exposure Activity Ratios (EAR) for those compounds with exact Chemical Abstract Service (CAS) number matches and with reliable concentration-response relationship and ACC data in ToxCast, Minnesota Tapwater Exposure Study 2019.

Page 49: Table S10. Site-specific Exposure Activity Ratios (EAR) for all bioassay endpoints within each class shown. Data are for those compounds with exact Chemical Abstract Service (CAS) number matches and with reliable concentration-response relationship and ACC data in ToxCast, Minnesota Tapwater Exposure Study 2019.

Page 50: Table S11. Site specific toxicity quotient(s) (TQ) for human-health benchmarks (lowest concentration available from MCL, MCLG, WHO, AAP, American Academy of Pediatrics, HBSL, or HHBP benchmarks) for each compound shown. TQ is the ratio of the measured concentration to the specific benchmark selected. See Table S12 for benchmark list and links.

Page 51: Table S12. Human-health-based benchmarks in microgram/L (µg/L) used to create toxicity quotient results in Table S11.

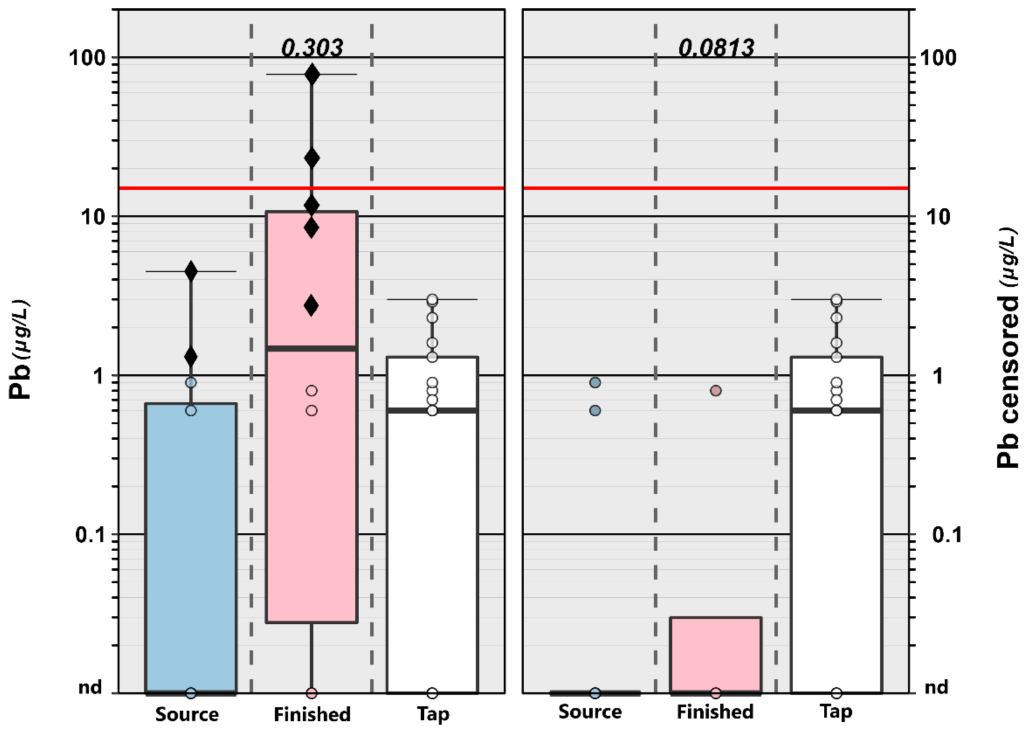

Figure S1. Comparison of lead concentrations in source water (blue boxes), finished water (pink boxes) and service-area tapwater (white boxes) using all data including locations where samples were collected from non-potable faucets (left) and potable faucets only (right). Red line is the non-health-based National Primary Drinking Water Regulation Technology Treatment Action Level. Circles are data for individual samples (both plots) and diamonds (left plot only) are samples collected from non-potable brass faucets and were removed from the analysis. Boxes, centerlines, and whiskers indicate interquartile range, median, and 5th and 95th percentiles, respectively. Numbers above each boxplot pair indicate the permuted probability that the centroids and dispersions are the same (PERMANOVA; 9999 permutations).

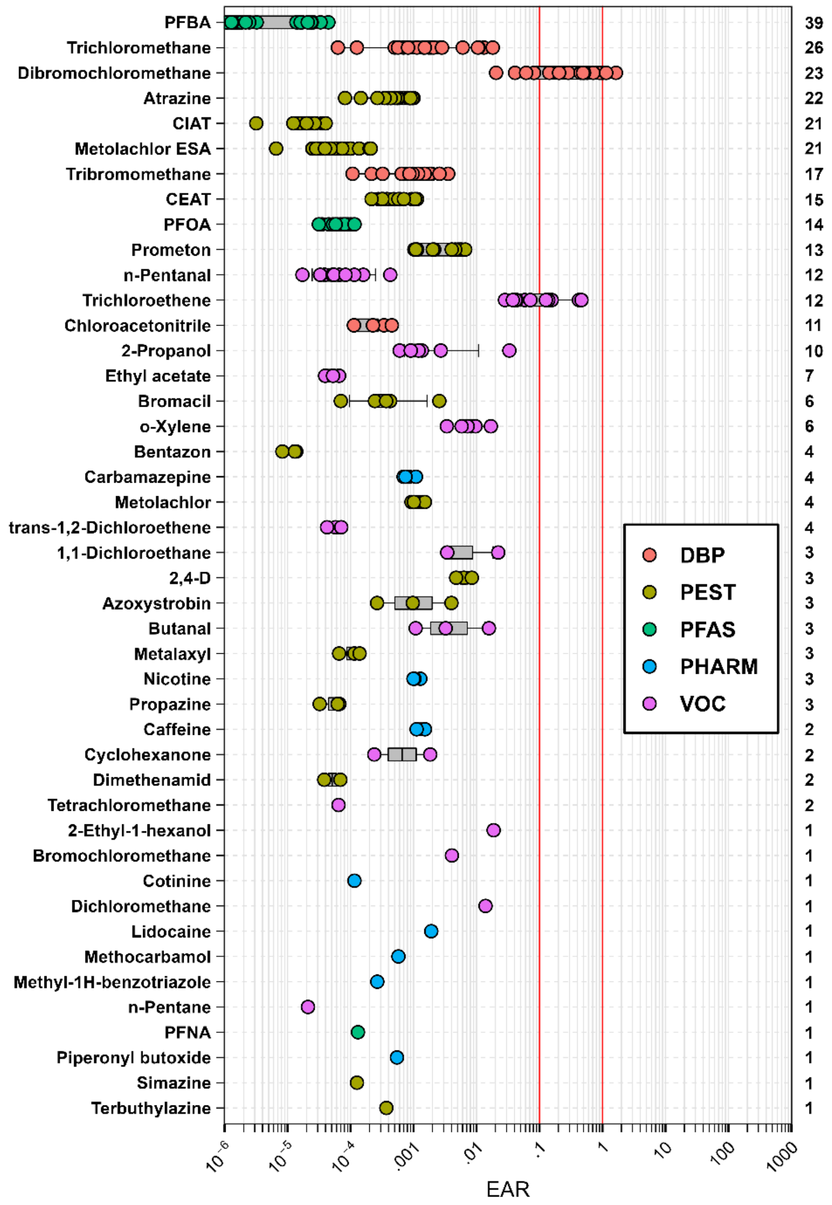

Figure S2. Exposure-activity ratios (EAR) and number of samples (right axes) for organic analytes (left axis, in order of decreasing total detections) detected in source water, finished water and service-area tapwater samples collected from Minnesota, 2019. Circles are data for individual samples. Solid red and yellow lines indicate concentrations shown to modulate effects in vitro and effects-screening-level thresholds (EAR = 0.001), respectively. Boxes, centerlines, and whiskers indicate interquartile range, median, and 5th and 95th percentiles, respectively. [DBP, disinfection byproducts; PEST, pesticides; PFAS, per-and polyfluoroalkyl substances; PHARM, pharmaceuticals; VOC, volatile organic chemicals.]

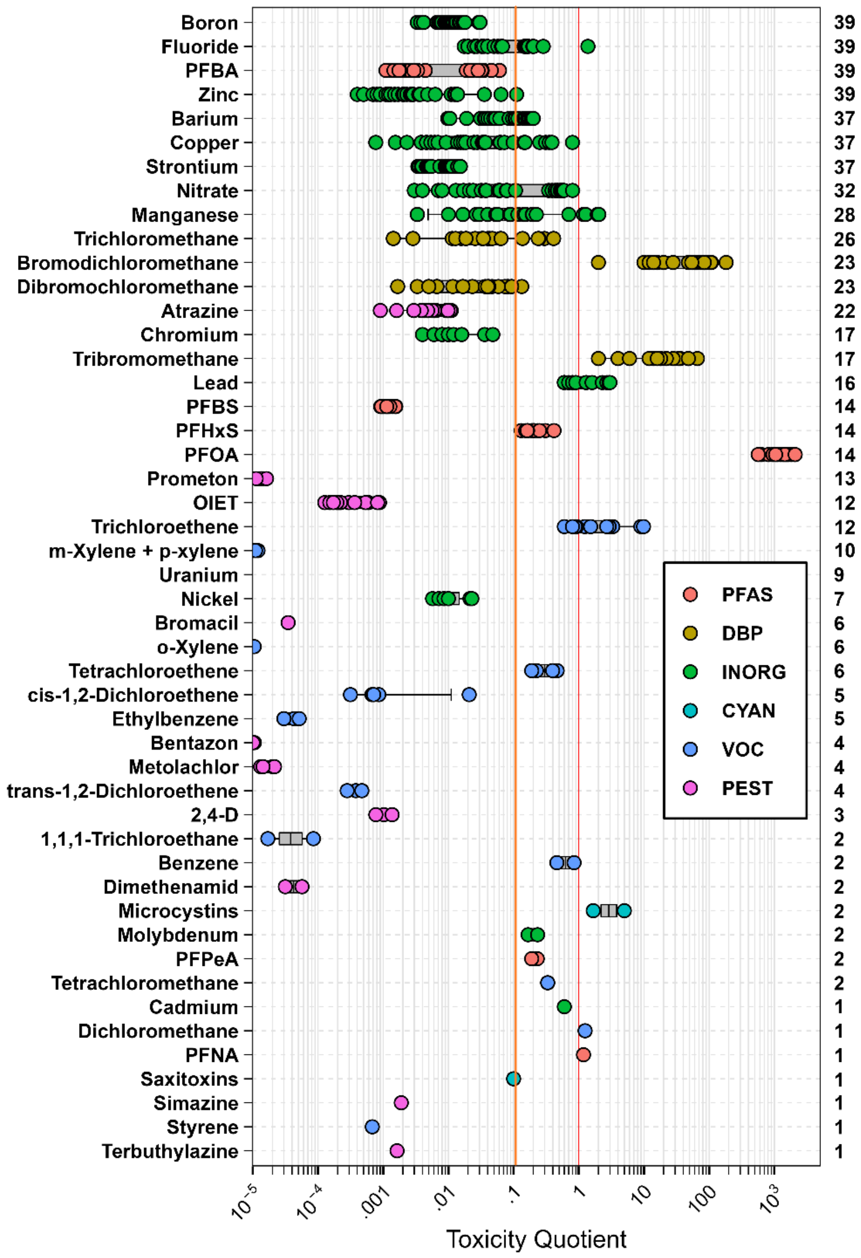

Figure S3. Toxicity Quotients (TQ) and number of samples (right axes) for organic analytes (left axis, in order of decreasing total detections) detected in source water, finished water, and service-area tapwater samples collected from Minnesota, 2019. Circles are data for individual samples. Solid red and yellow lines indicate benchmark equivalent concentrations and effects-screening-level threshold of concern (TQ = 0.1), respectively. Boxes, centerlines, and whiskers indicate interquartile range, median, and 5th and 95th percentiles, respectively. [DBP, disinfection byproducts; INORG, inorganics (ions, trace elements); PEST, pesticides; PFAS, per- and polyfluoroalkyl substances; VOC, volatile organic chemicals.]

Table S1. Site information for samples collected as part of the U.S. Geological Survey (USGS) Ecosystems Mission Area, Environmental Health Infrastructure Project, Minnesota Tapwater Exposure study in, 2019.Quantitation and method detection limits can be found in references cited.

[--; data not available]

Site code	Site type	Sampling date (yyyymmdd)	Sampling time (hhmm)	Source of water	Well depth (feet)
MNTW2019 023	Source water	20190826	1400	Surface water	--
MNTW2019 022	Finished water	20190826	1320	Surface water	--
MNTW2019 025	Tapwater	20190826	1430	Surface water	--
MNTW2019 024	Tapwater	20190826	1500	Surface water	--
MNTW2019 010	Source water	20190820	1040	Groundwater	403
MNTW2019 009	Finished water	20190820	1010	Groundwater	403
MNTW2019 007	Tapwater	20190820	0910	Groundwater	403
MNTW2019 008	Tapwater	20190820	0940	Groundwater	403
MNTW2019 027	Source water	20190827	1030	Groundwater	355
MNTW2019 026	Finished water	20190827	1010	Groundwater	355
MNTW2019 030	Tapwater	20190827	1100	Groundwater	355
MNTW2019 028	Source water	20190827	0910	Groundwater	285
MNTW2019 029	Finished water	20190827	0930	Groundwater	285
MNTW2019 031	Tapwater	20190827	1130	Groundwater	285
MNTW2019 037	Source water	20190828	1000	Groundwater	157
MNTW2019 036	Finished water	20190828	0920	Groundwater	157
MNTW2019 038	Tapwater	20190828	1030	Groundwater	157
MNTW2019 039	Tapwater	20190828	1110	Groundwater	157
MNTW2019 011	Source water	20190820	1310	Surface water	--
MNTW2019 012	Finished water	20190820	1340	Surface water	--
MNTW2019 013	Tapwater	20190820	1440	Surface water	--
MNTW2019 014	Tapwater	20190820	1530	Surface water	--
MNTW2019 020	Source water	20190826	1100	Groundwater	290
MNTW2019 019	Finished water	20190826	1030	Groundwater	290
MNTW2019 006	Tapwater	20190826	1150	Groundwater	222
MNTW2019 018	Source water	20190826	0950	Groundwater	222
MNTW2019 017	Finished water	20190826	0920	Groundwater	222
MNTW2019 021	Tapwater	20190826	1220	Groundwater	222
MNTW2019 002	Source water	20190819	1320	Groundwater	342
MNTW2019 005	Tapwater	20190819	1420	Groundwater	342
MNTW2019 004	Source water	20190819	1120	Surface water	--
MNTW2019 003	Finished water	20190819	1010	Surface water	--
MNTW2019 001	Tapwater	20190819	0920	Surface water	--

MNTW2019 016	Source water	20190821	0930	Groundwater	152
MNTW2019 015	Tapwater	20190821	0900	Groundwater	152
MNTW2019 033	Source water	20190827	1310	Groundwater	460
MNTW2019 032	Finished water	20190827	1250	Groundwater	460
MNTW2019 034	Tapwater	20190827	1340	Groundwater	460
MNTW2019 035	Tapwater	20190827	1410	Groundwater	460

Table S2. Method information for compounds analyzed for the U.S. Geological Survey (USGS) Ecosystems Mission Area, Environmental Health Infrastructure Project, Minnesota Tapwater Exposure study, 2019.

[CAS, Chemical Abstract Services; y, year; RL, reporting limit; NA, not available; EPA, U.S. Environmental Protection Agency; RED01, enzyme reduction-diazotization; AAS, auto hydride; GC, gas chromatograph; LC, liquid chromatography; ICP-MS, inductively coupled plasma- mass spectrometry; cICP-MS, cell inductively coupled plasma-mass spectrometry; HPLC/MS-MS, high performance liquid chromatography-tandem mass spectrometry; HCO3, bicarbonate; µS/cm, microsiemens per centimeter; ug/l, micrograms per liter; mg/L, milligrams per liter; ng/L, nanograms per liter; DAI-LC/MS-MS, direct aqueous injection-liquid chromatography/tandem mass spectrometry; ICP-OES, inductively coupled plasma-optical emission spectrometry; IC, ion chromatography; GCM66, ambient purgeable method (GC/MS); GM016, heat purgeable method (GC/MS); GC/MS-MS, gas chromatography, tandem mass spectrometry; LC/MS-MS, liquid chromatography-tandem mass spectrometry; ELISA, enzyme linked immunosorbent assay for algal toxins]

Parameter Name	Common/primary use/group	Analyzing laboratory	Method	CAS number	Reporting limit	Units of measurement	Method citation
1,7-Dimethylxanthine (p-Xanthine)	Pharmaceutical	NWQL	LC/MS-MS	611-59-6	88	ng/L	Furlong and others, 2014
10-Hydroxy-amitriptyline	Pharmaceutical	NWQL	LC/MS-MS	64520-05-4	8.3	ng/L	Furlong and others, 2014
Abacavir	Pharmaceutical	NWQL	LC/MS-MS	136470-78-5	2	ng/L	Furlong and others, 2014
Acetaminophen	Pharmaceutical	NWQL	LC/MS-MS	103-90-2	20	ng/L	Furlong and others, 2014
Acyclovir	Pharmaceutical	NWQL	LC/MS-MS	59277-89-3	22	ng/L	Furlong and others, 2014
Albuterol	Pharmaceutical	NWQL	LC/MS-MS	18559-94-9	6.7	ng/L	Furlong and others, 2014
Alprazolam	Pharmaceutical	NWQL	LC/MS-MS	28981-97-7	21	ng/L	Furlong and others, 2014
Amitriptyline	Pharmaceutical	NWQL	LC/MS-MS	50-48-6	37	ng/L	Furlong and others, 2014
Amphetamine	Pharmaceutical	NWQL	LC/MS-MS	300-62-9	4.4	ng/L	Furlong and others, 2014
Antipyrine	Pharmaceutical	NWQL	LC/MS-MS	60-80-0	50	ng/L	Furlong and others, 2014
Atenolol	Pharmaceutical	NWQL	LC/MS-MS	29122-68-7	13	ng/L	Furlong and others, 2014
Atrazine	Pesticide	NWQL	LC/MS-MS	1912-24-9	20	ng/L	Furlong and others, 2014
Benztropine	Pharmaceutical	NWQL	LC/MS-MS	86-13-5	44	ng/L	Furlong and others, 2014

Parameter Name	Common/primary use/group	Analyzing laboratory	Method	CAS number	Reporting limit	Units of measurement	Method citation
Betamethasone	Pharmaceutical	NWQL	LC/MS-MS	378-44-9	114	ng/L	Furlong and others, 2014
Bupropion	Pharmaceutical	NWQL	LC/MS-MS	34911-55-2	18	ng/L	Furlong and others, 2014
Caffeine	Pharmaceutical	NWQL	LC/MS-MS	58-08-2	91	ng/L	Furlong and others, 2014
Carbamazepine	Pharmaceutical	NWQL	LC/MS-MS	298-46-4	11	ng/L	Furlong and others, 2014
Carisoprodol	Pharmaceutical	NWQL	LC/MS-MS	78-44-4	20	ng/L	Furlong and others, 2014
Chlorpheniramine	Pharmaceutical	NWQL	LC/MS-MS	132-22-9	54	ng/L	Furlong and others, 2014
Cimetidine	Pharmaceutical	NWQL	LC/MS-MS	51481-61-9	140	ng/L	Furlong and others, 2014
Citalopram	Pharmaceutical	NWQL	LC/MS-MS	59729-33-8	6.6	ng/L	Furlong and others, 2014
Clonidine	Pharmaceutical	NWQL	LC/MS-MS	4205-90-7	61	ng/L	Furlong and others, 2014
Codeine	Pharmaceutical	NWQL	LC/MS-MS	76-57-3	32	ng/L	Furlong and others, 2014
Cotinine	Pharmaceutical	NWQL	LC/MS-MS	486-56-6	6.4	ng/L	Furlong and others, 2014
Dehydronifedipine	Pharmaceutical	NWQL	LC/MS-MS	67035-22-7	20	ng/L	Furlong and others, 2014
Desmethyldiltiazem	Pharmaceutical	NWQL	LC/MS-MS	-	70	ng/L	Furlong and others, 2014
Desvenlafaxine	Pharmaceutical	NWQL	LC/MS-MS	93413-62-8	84	ng/L	Furlong and others, 2014
Dextromethorphan	Pharmaceutical	NWQL	LC/MS-MS	125-71-3	8.2	ng/L	Furlong and others, 2014
Diazepam (valium)	Pharmaceutical	NWQL	LC/MS-MS	439-14-5	4.0	ng/L	Furlong and others, 2014
Diltiazem	Pharmaceutical	NWQL	LC/MS-MS	42399-41-7	10	ng/L	Furlong and others, 2014
Diphenhydramine	Pharmaceutical	NWQL	LC/MS-MS	147-24-0	48	ng/L	Furlong and others, 2014

Parameter Name	Common/primary use/group	Analyzing laboratory	Method	CAS number	Reporting limit	Units of measurement	Method citation
Duloxetine	Pharmaceutical	NWQL	LC/MS-MS	116539-59-4	37	ng/L	Furlong and others, 2014
Erythromycin	Pharmaceutical	NWQL	LC/MS-MS	114-07-8	80	ng/L	Furlong and others, 2014
Ezetimibe	Pharmaceutical	NWQL	LC/MS-MS	163222-33-1	205	ng/L	Furlong and others, 2014
Fadrozole	Pharmaceutical	NWQL	LC/MS-MS	102676-47-1	13	ng/L	Furlong and others, 2014
Famotidine	Pharmaceutical	NWQL	LC/MS-MS	76824-35-6	34	ng/L	Furlong and others, 2014
Fenofibrate	Pharmaceutical	NWQL	LC/MS-MS	49562-28-9	6.40 - 14.0	ng/L	Furlong and others, 2014
Fexofenadine	Pharmaceutical	NWQL	LC/MS-MS	83799-24-0	44.0 - 96.0	ng/L	Furlong and others, 2014
Fluconazole	Pharmaceutical	NWQL	LC/MS-MS	86386-73-4	30	ng/L	Furlong and others, 2014
Fluoxetine	Pharmaceutical	NWQL	LC/MS-MS	54910-89-3	26	ng/L	Furlong and others, 2014
Fluticasone propionate	Pharmaceutical	NWQL	LC/MS-MS	80474-14-2	30	ng/L	Furlong and others, 2014
Fluvoxamine	Pharmaceutical	NWQL	LC/MS-MS	54739-18-3	80	ng/L	Furlong and others, 2014
Gabapentin	Pharmaceutical	NWQL	LC/MS-MS	60142-96-3	160	ng/L	Furlong and others, 2014
Glipizide	Pharmaceutical	NWQL	LC/MS-MS	29094-61-9	80	ng/L	Furlong and others, 2014
Glyburide	Pharmaceutical	NWQL	LC/MS-MS	10238-21-8	58	ng/L	Furlong and others, 2014
Guanylurea	Pharmaceutical	NWQL	LC/MS-MS	141-83-3	140 - 400	ng/L	Furlong and others, 2014
Hydrocodone	Pharmaceutical	NWQL	LC/MS-MS	125-29-1	40	ng/L	Furlong and others, 2014
Hydrocortisone	Pharmaceutical	NWQL	LC/MS-MS	50-23-7	147	ng/L	Furlong and others, 2014
Hydroxyzine	Pharmaceutical	NWQL	LC/MS-MS	68-88-2	7.4	ng/L	Furlong and others, 2014

Parameter Name	Common/primary use/group	Analyzing laboratory	Method	CAS number	Reporting limit	Units of measurement	Method citation
Iminostilbene	Pharmaceutical	NWQL	LC/MS-MS	256-96-2	145	ng/L	Furlong and others, 2014
Ketoconazole	Pharmaceutical	NWQL	LC/MS-MS	65277-42-1	113	ng/L	Furlong and others, 2014
Lamivudine	Pharmaceutical	NWQL	LC/MS-MS	134678-17-4	16	ng/L	Furlong and others, 2014
Lidocaine	Pharmaceutical	NWQL	LC/MS-MS	137-58-6	8.00 - 38.0	ng/L	Furlong and others, 2014
Loperamide	Pharmaceutical	NWQL	LC/MS-MS	53179-11-6	80	ng/L	Furlong and others, 2014
Loratadine	Pharmaceutical	NWQL	LC/MS-MS	79794-75-5	7.0	ng/L	Furlong and others, 2014
Lorazepam	Pharmaceutical	NWQL	LC/MS-MS	846-49-1	202	ng/L	Furlong and others, 2014
Meprobamate	Pharmaceutical	NWQL	LC/MS-MS	57-53-4	12.0 - 86.0	ng/L	Furlong and others, 2014
Metaxalone	Pharmaceutical	NWQL	LC/MS-MS	1665-48-1	16	ng/L	Furlong and others, 2014
Metformin	Pharmaceutical	NWQL	LC/MS-MS	657-24-9	13	ng/L	Furlong and others, 2014
Methadone	Pharmaceutical	NWQL	LC/MS-MS	76-99-3	7.6	ng/L	Furlong and others, 2014
methocarbamol	Pharmaceutical	NWQL	LC/MS-MS	532-03-6	11	ng/L	Furlong and others, 2014
Methotrexate	Pharmaceutical	NWQL	LC/MS-MS	59-05-2	52	ng/L	Furlong and others, 2014
methyl-1H-benzotriazole	Pharmaceutical	NWQL	LC/MS-MS	29385-43-1	80	ng/L	Furlong and others, 2014
Metoprolol	Pharmaceutical	NWQL	LC/MS-MS	51384-51-1	10	ng/L	Furlong and others, 2014
Morphine	Pharmaceutical	NWQL	LC/MS-MS	57-27-2	80	ng/L	Furlong and others, 2014
Nadalol	Pharmaceutical	NWQL	LC/MS-MS	42200-33-9	20	ng/L	Furlong and others, 2014
Nevirapine	Pharmaceutical	NWQL	LC/MS-MS	129618-40-2	46	ng/L	Furlong and others, 2014

Parameter Name	Common/primary use/group	Analyzing laboratory	Method	CAS number	Reporting limit	Units of measurement	Method citation
nicotine	Pharmaceutical	NWQL	LC/MS-MS	54-11-5	58	ng/L	Furlong and others, 2014
Nizatidine	Pharmaceutical	NWQL	LC/MS-MS	76963-41-2	80	ng/L	Furlong and others, 2014
Nordiazepam	Pharmaceutical	NWQL	LC/MS-MS	1088-11-5	20	ng/L	Furlong and others, 2014
Norethindrone	Pharmaceutical	NWQL	LC/MS-MS	68-22-4	20	ng/L	Furlong and others, 2014
Norfluoxetine	Pharmaceutical	NWQL	LC/MS-MS	56161-73-0	80	ng/L	Furlong and others, 2014
Norsertraline	Pharmaceutical	NWQL	LC/MS-MS	87857-41-8	80	ng/L	Furlong and others, 2014
Norverapamil	Pharmaceutical	NWQL	LC/MS-MS	67018-85-3	8.6	ng/L	Furlong and others, 2014
Omeprazole + Esomprazole	Pharmaceutical	NWQL	LC/MS-MS	-	16	ng/L	Furlong and others, 2014
Oseltamivir	Pharmaceutical	NWQL	LC/MS-MS	196618-13-0	15	ng/L	Furlong and others, 2014
Oxazepam	Pharmaceutical	NWQL	LC/MS-MS	604-75-1	226	ng/L	Furlong and others, 2014
Oxycodone	Pharmaceutical	NWQL	LC/MS-MS	76-42-6	25	ng/L	Furlong and others, 2014
Paroxetine	Pharmaceutical	NWQL	LC/MS-MS	61869-08-7	72.0 - 264	ng/L	Furlong and others, 2014
Penciclovir	Pharmaceutical	NWQL	LC/MS-MS	39809-25-1	80	ng/L	Furlong and others, 2014
Pentoxifylline	Pharmaceutical	NWQL	LC/MS-MS	6493-05-6	9.4	ng/L	Furlong and others, 2014
Phenazopyridine	Pharmaceutical	NWQL	LC/MS-MS	94-78-0	13	ng/L	Furlong and others, 2014
Phendimetrazine	Pharmaceutical	NWQL	LC/MS-MS	634-03-7	20.0 - 31.0	ng/L	Furlong and others, 2014
Phenytoin	Pharmaceutical	NWQL	LC/MS-MS	57-41-0	188	ng/L	Furlong and others, 2014
Piperonyl butoxide	Pharmaceutical	NWQL	LC/MS-MS	51-03-6	60	ng/L	Furlong and others, 2014

Parameter Name	Common/primary use/group	Analyzing laboratory	Method	CAS number	Reporting limit	Units of measurement	Method citation
Prednisolone	Pharmaceutical	NWQL	LC/MS-MS	50-24-8	150	ng/L	Furlong and others, 2014
Prednisone	Pharmaceutical	NWQL	LC/MS-MS	53-03-2	105 - 168	ng/L	Furlong and others, 2014
Promethazine	Pharmaceutical	NWQL	LC/MS-MS	60-87-7	114	ng/L	Furlong and others, 2014
Propoxyphene	Pharmaceutical	NWQL	LC/MS-MS	469-62-5	28	ng/L	Furlong and others, 2014
Propranolol	Pharmaceutical	NWQL	LC/MS-MS	525-66-6	26	ng/L	Furlong and others, 2014
Pseudoephedrine + Ephedrine	Pharmaceutical	NWQL	LC/MS-MS	-	6	ng/L	Furlong and others, 2014
Quinine	Pharmaceutical	NWQL	LC/MS-MS	130-95-0	80	ng/L	Furlong and others, 2014
Ractopamine	Pharmaceutical	NWQL	LC/MS-MS	97825-25-7	20	ng/L	Furlong and others, 2014
Raloxifene	Pharmaceutical	NWQL	LC/MS-MS	84449-90-1	80	ng/L	Furlong and others, 2014
Ranitidine	Pharmaceutical	NWQL	LC/MS-MS	66357-35-5	192	ng/L	Furlong and others, 2014
Sertraline	Pharmaceutical	NWQL	LC/MS-MS	79617-96-2	16	ng/L	Furlong and others, 2014
Sitagliptin	Pharmaceutical	NWQL	LC/MS-MS	486460-32-6	97	ng/L	Furlong and others, 2014
Sulfadimethoxine	Pharmaceutical	NWQL	LC/MS-MS	122-11-2	30	ng/L	Furlong and others, 2014
Sulfamethizole	Pharmaceutical	NWQL	LC/MS-MS	144-82-1	104	ng/L	Furlong and others, 2014
Sulfamethoxazole	Pharmaceutical	NWQL	LC/MS-MS	723-46-6	20.0 - 26.0	ng/L	Furlong and others, 2014
Tamoxifen	Pharmaceutical	NWQL	LC/MS-MS	10540-29-1	270	ng/L	Furlong and others, 2014
Temazepam	Pharmaceutical	NWQL	LC/MS-MS	846-50-4	18	ng/L	Furlong and others, 2014
Theophylline	Pharmaceutical	NWQL	LC/MS-MS	58-55-9	80	ng/L	Furlong and others, 2014

Parameter Name	Common/primary use/group	Analyzing laboratory	Method	CAS number	Reporting limit	Units of measurement	Method citation
Thiabendazole	Pharmaceutical	NWQL	LC/MS-MS	148-79-8	4.00 - 11.0	ng/L	Furlong and others, 2014
Tiotropium	Pharmaceutical	NWQL	LC/MS-MS	186691-13-4	50.0 - 200	ng/L	Furlong and others, 2014
Tramadol	Pharmaceutical	NWQL	LC/MS-MS	27203-92-5	7.4	ng/L	Furlong and others, 2014
Triamterene	Pharmaceutical	NWQL	LC/MS-MS	396-01-0	5.2	ng/L	Furlong and others, 2014
Trimethoprim	Pharmaceutical	NWQL	LC/MS-MS	738-70-5	20	ng/L	Furlong and others, 2014
Valacyclovir	Pharmaceutical	NWQL	LC/MS-MS	124832-26-4	163	ng/L	Furlong and others, 2014
Venlafaxine	Pharmaceutical	NWQL	LC/MS-MS	93413-69-5	5.2	ng/L	Furlong and others, 2014
Verapamil	Pharmaceutical	NWQL	LC/MS-MS	52-53-9	140	ng/L	Furlong and others, 2014
Warfarin	Pharmaceutical	NWQL	LC/MS-MS	81-81-2	6.0	ng/L	Furlong and others, 2014
1H-1,2,4-Triazole	Pesticide	NWQL	LC/MS-MS	288-88-0	22	ng/L	Sandstrom and others, 2015
2,4-D	Pesticide	NWQL	LC/MS-MS	94-75-7	62	ng/L	Sandstrom and others, 2015
2-(1-Hydroxyethyl)-6-methylaniline	Pesticide	NWQL	LC/MS-MS	196611-19-5	54.0	ng/L	Sandstrom and others, 2015
2-[(2-Ethyl-6-methylphenyl)amino]-1-propanol	Pesticide	NWQL	LC/MS-MS	61520-53-4	5.00	ng/L	Sandstrom and others, 2015
2-Amino-N-isopropylbenzamide	Pesticide	NWQL	LC/MS-MS	30391-89-0	4.0	ng/L	Sandstrom and others, 2015
2-Aminobenzimidazole	Pesticide	NWQL	LC/MS-MS	934-32-7	9	ng/L	Sandstrom and others, 2015
2-Chloro-2',6'-diethylacetanilide	Pesticide	NWQL	LC/MS-MS	6967-29-9	5.00	ng/L	Sandstrom and others, 2015
2-Chloro-4,6-diamino-s-triazine {CAAT, Didealkylatrazine)	Pesticide	NWQL	LC/MS-MS	3397-62-4	24.0	ng/L	Sandstrom and others, 2015

Parameter Name	Common/primary use/group	Analyzing laboratory	Method	CAS number	Reporting limit	Units of measurement	Method citation
2-Chloro-4-isopropylamino-6-amino-s-triazine {CIAT, desethyl atrazine}	Pesticide	NWQL	LC/MS-MS	6190-65-4	11.0	ng/L	Sandstrom and others, 2015
2-Chloro-6-ethylamino-4-amino-s-triazine {CEAT, deisopropylatrazine}	Pesticide	NWQL	LC/MS-MS	1007-28-9	20.0	ng/L	Sandstrom and others, 2015
2-Chloro-N-(2-ethyl-6-methylphenyl)acetamide	Pesticide	NWQL	LC/MS-MS	32428-71-0	5.00	ng/L	Sandstrom and others, 2015
2-Hydroxy-4-isopropylamino-6-amino-s-triazine {OIAT, atrazine-desethyl-2-hydroxy}	Pesticide	NWQL	LC/MS-MS	19988-24-0	4.00	ng/L	Sandstrom and others, 2015
2-Hydroxy-4-isopropylamino-6-ethylamino-s-triazine {OIET, 2-hydroxyatrazine}	Pesticide	NWQL	LC/MS-MS	2163-68-0	8.00	ng/L	Sandstrom and others, 2015
2-Hydroxy-6-ethylamino-4-amino-s-triazine {OEAT, disopropylhydroxyatrazine}	Pesticide	NWQL	LC/MS-MS	7313-54-4	100	ng/L	Sandstrom and others, 2015
2-Isopropyl-6-methyl-4-pyrimidinol	Pesticide	NWQL	LC/MS-MS	2814-20-2	8	ng/L	Sandstrom and others, 2015
3,4-Dichlorophenylurea	Pesticide	NWQL	LC/MS-MS	2327-02-8	108	ng/L	Sandstrom and others, 2015
3-Hydroxycarbofuran	Pesticide	NWQL	LC/MS-MS	16655-82-6	16	ng/L	Sandstrom and others, 2015
3-Phenoxybenzoic acid	Pesticide	NWQL	LC/MS-MS	3739-38-6	61	ng/L	Sandstrom and others, 2015

Parameter Name	Common/primary use/group	Analyzing laboratory	Method	CAS number	Reporting limit	Units of measurement	Method citation
4-(Hydroxymethyl)pendimethalin	Pesticide	NWQL	LC/MS-MS	56750-76-6	114	ng/L	Sandstrom and others, 2015
4-Chlorobenzylmethyl sulfoxide	Pesticide	NWQL	LC/MS-MS	24176-68-9	3.2	ng/L	Sandstrom and others, 2015
4-Hydroxychlorothalonil	Pesticide	NWQL	LC/MS-MS	28343-61-5	98.0	ng/L	Sandstrom and others, 2015
4-Hydroxyhexazinone A	Pesticide	NWQL	LC/MS-MS	72576-13-7	3.00	ng/L	Sandstrom and others, 2015
Acephate	Pesticide	NWQL	LC/MS-MS	30560-19-1	10.0	ng/L	Sandstrom and others, 2015
Acetochlor	Pesticide	NWQL	LC/MS-MS	34256-82-1	10.0	ng/L	Sandstrom and others, 2015
Acetochlor oxanilic acid	Pesticide	NWQL	LC/MS-MS	194992-44-4	65.0	ng/L	Sandstrom and others, 2015
Acetochlor sulfonic acid	Pesticide	NWQL	LC/MS-MS	187022-11-3	320	ng/L	Sandstrom and others, 2015
Acetochlor sulfynilacetic acid	Pesticide	NWQL	LC/MS-MS	618113-86-3	176	ng/L	Sandstrom and others, 2015
Alachlor	Pesticide	NWQL	LC/MS-MS	15972-60-8	27.0	ng/L	Sandstrom and others, 2015
Alachlor oxanilic acid	Pesticide	NWQL	LC/MS-MS	171262-17-2	60.0	ng/L	Sandstrom and others, 2015
Alachlor sulfonic acid	Pesticide	NWQL	LC/MS-MS	142363-53-9	360	ng/L	Sandstrom and others, 2015
Alachlor sulfynilacetic acid	Pesticide	NWQL	LC/MS-MS	494847-39-1	128	ng/L	Sandstrom and others, 2015
Aldicarb	Pesticide	NWQL	LC/MS-MS	116-06-3	8.00	ng/L	Sandstrom and others, 2015
Aldicarb sulfone	Pesticide	NWQL	LC/MS-MS	1646-88-4	20.0	ng/L	Sandstrom and others, 2015
Aldicarb sulfoxide	Pesticide	NWQL	LC/MS-MS	1646-87-3	2.20	ng/L	Sandstrom and others, 2015
Ametryn	Pesticide	NWQL	LC/MS-MS	834-12-8	2.60	ng/L	Sandstrom and others, 2015

Parameter Name	Common/primary use/group	Analyzing laboratory	Method	CAS number	Reporting limit	Units of measurement	Method citation
Asulam	Pesticide	NWQL	LC/MS-MS	3337-71-1	50.0	ng/L	Sandstrom and others, 2015
Atrazine	Pesticide	NWQL	LC/MS-MS	1912-24-9	6.8	ng/L	Sandstrom and others, 2015
Azinphos-methyl	Pesticide	NWQL	LC/MS-MS	86-50-0	8.00	ng/L	Sandstrom and others, 2015
Azinphos-methyl oxon	Pesticide	NWQL	LC/MS-MS	961-22-8	15.0	ng/L	Sandstrom and others, 2015
Azoxystrobin	Pesticide	NWQL	LC/MS-MS	131860-33-8	3.00	ng/L	Sandstrom and others, 2015
Bentazon	Pesticide	NWQL	LC/MS-MS	25057-89-0	9.00	ng/L	Sandstrom and others, 2015
Bifenthrin	Pesticide	NWQL	LC/MS-MS	82657-04-3	19.0	ng/L	Sandstrom and others, 2015
Bromacil	Pesticide	NWQL	LC/MS-MS	314-40-9	5.60	ng/L	Sandstrom and others, 2015
Bromoxynil	Pesticide	NWQL	LC/MS-MS	1689-84-5	60.0	ng/L	Sandstrom and others, 2015
Butralin	Pesticide	NWQL	LC/MS-MS	33629-47-9	5.00	ng/L	Sandstrom and others, 2015
Butylate	Pesticide	NWQL	LC/MS-MS	2008-41-5	10.0	ng/L	Sandstrom and others, 2015
Carbaryl	Pesticide	NWQL	LC/MS-MS	63-25-2	5.60	ng/L	Sandstrom and others, 2015
Carbendazim	Pesticide	NWQL	LC/MS-MS	10605-21-7	10.0	ng/L	Sandstrom and others, 2015
Carbofuran	Pesticide	NWQL	LC/MS-MS	1563-66-2	5.00	ng/L	Sandstrom and others, 2015
Chlorimuron-ethyl	Pesticide	NWQL	LC/MS-MS	90982-32-4	8.80	ng/L	Sandstrom and others, 2015
Chlorpyrifos	Pesticide	NWQL	LC/MS-MS	2921-88-2	3.00	ng/L	Sandstrom and others, 2015
Chlorpyrifos oxon	Pesticide	NWQL	LC/MS-MS	-	4.40	ng/L	Sandstrom and others, 2015
Chlorsulfuron	Pesticide	NWQL	LC/MS-MS	64902-72-3	50.0	ng/L	Sandstrom and others, 2015

Parameter Name	Common/primary use/group	Analyzing laboratory	Method	CAS number	Reporting limit	Units of measurement	Method citation
cis-Bifenthrin acid/cis-Cyhalothrin acid/cis-Tefluthrin acid	Pesticide	NWQL	LC/MS-MS	68127-59-3	105	ng/L	Sandstrom and others, 2015
cis-Permethrin	Pesticide	NWQL	LC/MS-MS	61949-76-6	4.20	ng/L	Sandstrom and others, 2015
Cyanazine	Pesticide	NWQL	LC/MS-MS	21725-46-2	50.0	ng/L	Sandstrom and others, 2015
Dacthal monoacid	Pesticide	NWQL	LC/MS-MS	887-54-7	2700	ng/L	Sandstrom and others, 2015
Dechlorofipronil	Pesticide	NWQL	LC/MS-MS	-	3.80	ng/L	Sandstrom and others, 2015
Dechlorometolachlor	Pesticide	NWQL	LC/MS-MS	126605-22-9	2.00	ng/L	Sandstrom and others, 2015
Deiodo flubendiamide	Pesticide	NWQL	LC/MS-MS	1016160-78-3	10.0	ng/L	Sandstrom and others, 2015
Deisopropyl prometryn	Pesticide	NWQL	LC/MS-MS	4147-57-3	2.80	ng/L	Sandstrom and others, 2015
Demethyl fluometuron	Pesticide	NWQL	LC/MS-MS	3032-40-4	3.6	ng/L	Sandstrom and others, 2015
Demethyl hexazinone B	Pesticide	NWQL	LC/MS-MS	56611-54-2	3.00	ng/L	Sandstrom and others, 2015
Demethyl norflurazon	Pesticide	NWQL	LC/MS-MS	23576-24-1	4.00	ng/L	Sandstrom and others, 2015
Desamino metribuzin	Pesticide	NWQL	LC/MS-MS	35045-02-4	9.00	ng/L	Sandstrom and others, 2015
Desamino-diketo metribuzin	Pesticide	NWQL	LC/MS-MS	52236-30-3	200	ng/L	Sandstrom and others, 2015
Desulfinylfipronil	Pesticide	NWQL	LC/MS-MS	205650-65-3	3.80	ng/L	Sandstrom and others, 2015
Desulfinylfipronil amide	Pesticide	NWQL	LC/MS-MS	1115248-09-3	10.0	ng/L	Sandstrom and others, 2015
Diazinon	Pesticide	NWQL	LC/MS-MS	333-41-5	2.80	ng/L	Sandstrom and others, 2015
Diazinon oxon	Pesticide	NWQL	LC/MS-MS	-	4.00	ng/L	Sandstrom and others, 2015
Dicamba	Pesticide	NWQL	LC/MS-MS	1918-00-9	800	ng/L	Sandstrom and others, 2015

Parameter Name	Common/primary use/group	Analyzing laboratory	Method	CAS number	Reporting limit	Units of measurement	Method citation
Dichlorvos	Pesticide	NWQL	LC/MS-MS	-	52.0	ng/L	Sandstrom and others, 2015
Dicrotophos	Pesticide	NWQL	LC/MS-MS	141-66-2	4.00	ng/L	Sandstrom and others, 2015
Didemethyl hexazinone F	Pesticide	NWQL	LC/MS-MS	56611-55-3	10.0	ng/L	Sandstrom and others, 2015
Diflubenzuron	Pesticide	NWQL	LC/MS-MS	35367-38-5	6.00	ng/L	Sandstrom and others, 2015
Diflufenzopyr	Pesticide	NWQL	LC/MS-MS	109293-97-2	72.0	ng/L	Sandstrom and others, 2015
Diketonitrile-isoxaflutole	Pesticide	NWQL	LC/MS-MS	143701-75-1	24.0	ng/L	Sandstrom and others, 2015
Dimethenamid	Pesticide	NWQL	LC/MS-MS	87674-68-8	3.00	ng/L	Sandstrom and others, 2015
Dimethenamid oxanilic acid	Pesticide	NWQL	LC/MS-MS	380412-59-9	85.0	ng/L	Sandstrom and others, 2015
Dimethenamid SAA	Pesticide	NWQL	LC/MS-MS	-	189	ng/L	Sandstrom and others, 2015
Dimethenamid sulfonic acid	Pesticide	NWQL	LC/MS-MS	205939-58-8	79.0	ng/L	Sandstrom and others, 2015
Dimethoate	Pesticide	NWQL	LC/MS-MS	60-51-5	4.60	ng/L	Sandstrom and others, 2015
Disulfoton	Pesticide	NWQL	LC/MS-MS	298-04-4	11.0	ng/L	Sandstrom and others, 2015
Disulfoton oxon	Pesticide	NWQL	LC/MS-MS	126-75-0	2.00	ng/L	Sandstrom and others, 2015
Disulfoton oxon sulfone	Pesticide	NWQL	LC/MS-MS	2496-91-5	6.00	ng/L	Sandstrom and others, 2015
Disulfoton oxon sulfoxide	Pesticide	NWQL	LC/MS-MS	2496-92-6	6.00	ng/L	Sandstrom and others, 2015
Disulfoton sulfone	Pesticide	NWQL	LC/MS-MS	2497-06-5	9.00	ng/L	Sandstrom and others, 2015
Disulfoton sulfoxide	Pesticide	NWQL	LC/MS-MS	2497-07-6	4.00	ng/L	Sandstrom and others, 2015
Diuron	Pesticide	NWQL	LC/MS-MS	330-54-1	5.00	ng/L	Sandstrom and others, 2015

Parameter Name	Common/primary use/group	Analyzing laboratory	Method	CAS number	Reporting limit	Units of measurement	Method citation
EPTC	Pesticide	NWQL	LC/MS-MS	759-94-4	206	ng/L	Sandstrom and others, 2015
EPTC degradate R248722	Pesticide	NWQL	LC/MS-MS	65109-69-5	4.00	ng/L	Sandstrom and others, 2015
Ethoprophos	Pesticide	NWQL	LC/MS-MS	13194-48-4	5.00	ng/L	Sandstrom and others, 2015
Etoxazole	Pesticide	NWQL	LC/MS-MS	153233-91-1	4.20	ng/L	Sandstrom and others, 2015
Fenamiphos	Pesticide	NWQL	LC/MS-MS	22224-92-6	4.60	ng/L	Sandstrom and others, 2015
Fenamiphos sulfone	Pesticide	NWQL	LC/MS-MS	31972-44-8	5.00	ng/L	Sandstrom and others, 2015
Fenamiphos sulfoxide	Pesticide	NWQL	LC/MS-MS	31972-43-7	5.00	ng/L	Sandstrom and others, 2015
Fenbutatin oxide	Pesticide	NWQL	LC/MS-MS	13356-08-6	120	ng/L	Sandstrom and others, 2015
Fentin	Pesticide	NWQL	LC/MS-MS	668-34-8	30.0	ng/L	Sandstrom and others, 2015
Fipronil	Pesticide	NWQL	LC/MS-MS	120068-37-3	4.00	ng/L	Sandstrom and others, 2015
Fipronil amide	Pesticide	NWQL	LC/MS-MS	205650-69-7	9.20	ng/L	Sandstrom and others, 2015
Fipronil sulfide	Pesticide	NWQL	LC/MS-MS	120067-83-6	4.20	ng/L	Sandstrom and others, 2015
Fipronil sulfonate	Pesticide	NWQL	LC/MS-MS	209248-72-6	96.0	ng/L	Sandstrom and others, 2015
Fipronil sulfone	Pesticide	NWQL	LC/MS-MS	120068-36-2	5.60	ng/L	Sandstrom and others, 2015
Flubendiamide	Pesticide	NWQL	LC/MS-MS	272451-65-7	4.40	ng/L	Sandstrom and others, 2015
Flumetsulam	Pesticide	NWQL	LC/MS-MS	98967-40-9	17.0	ng/L	Sandstrom and others, 2015
Fluometuron	Pesticide	NWQL	LC/MS-MS	2164-17-2	10.0	ng/L	Sandstrom and others, 2015
Fonofos	Pesticide	NWQL	LC/MS-MS	944-22-9	11.0	ng/L	Sandstrom and others, 2015

Parameter Name	Common/primary use/group	Analyzing laboratory	Method	CAS number	Reporting limit	Units of measurement	Method citation
Halosulfuron-methyl	Pesticide	NWQL	LC/MS-MS	100784-20-1	12.0	ng/L	Sandstrom and others, 2015
Hexazinone	Pesticide	NWQL	LC/MS-MS	51235-04-2	3.60	ng/L	Sandstrom and others, 2015
Hexazinone Transformation Product C	Pesticide	NWQL	LC/MS-MS	72585-88-7	2.00	ng/L	Sandstrom and others, 2015
Hexazinone Transformation Product D	Pesticide	NWQL	LC/MS-MS	30243-77-7	294	ng/L	Sandstrom and others, 2015
Hexazinone Transformation Product E	Pesticide	NWQL	LC/MS-MS	72576-14-8	76.0	ng/L	Sandstrom and others, 2015
Hexazinone Transformation Product G	Pesticide	NWQL	LC/MS-MS	-	22.0	ng/L	Sandstrom and others, 2015
Hydroxy didemethyl fluometuron	Pesticide	NWQL	LC/MS-MS	-	50.0	ng/L	Sandstrom and others, 2015
Hydroxy monodemethyl fluometuron	Pesticide	NWQL	LC/MS-MS	-	12.0	ng/L	Sandstrom and others, 2015
Hydroxyacetochlor	Pesticide	NWQL	LC/MS-MS	60090-47-3	20.0	ng/L	Sandstrom and others, 2015
Hydroxyalachlor	Pesticide	NWQL	LC/MS-MS	56681-55-1	6.00	ng/L	Sandstrom and others, 2015
Hydroxydiazinon	Pesticide	NWQL	LC/MS-MS	29820-16-4	11.0	ng/L	Sandstrom and others, 2015
Hydroxymetolachlor	Pesticide	NWQL	LC/MS-MS	131068-72-9	2.40	ng/L	Sandstrom and others, 2015
Hydroxyphthalazinone	Pesticide	NWQL	LC/MS-MS	-	28.0	ng/L	Sandstrom and others, 2015
Hydroxysimazine	Pesticide	NWQL	LC/MS-MS	2599-11-3	120	ng/L	Sandstrom and others, 2015
Imazamox	Pesticide	NWQL	LC/MS-MS	114311-32-9	30.0	ng/L	Sandstrom and others, 2015
Imazaquin	Pesticide	NWQL	LC/MS-MS	81335-37-7	18.0	ng/L	Sandstrom and others, 2015

Parameter Name	Common/primary use/group	Analyzing laboratory	Method	CAS number	Reporting limit	Units of measurement	Method citation
Imazethapyr	Pesticide	NWQL	LC/MS-MS	81335-77-5	8.00	ng/L	Sandstrom and others, 2015
Imidacloprid	Pesticide	NWQL	LC/MS-MS	138261-41-3	16.0	ng/L	Sandstrom and others, 2015
Indoxacarb	Pesticide	NWQL	LC/MS-MS	173584-44-6	5.20	ng/L	Sandstrom and others, 2015
Isoxaflutole	Pesticide	NWQL	LC/MS-MS	141112-29-0	18.0	ng/L	Sandstrom and others, 2015
Isoxaflutole acid metabolite RPA 203328	Pesticide	NWQL	LC/MS-MS	142994-06-7	9.20	ng/L	Sandstrom and others, 2015
Kresoxim-methyl	Pesticide	NWQL	LC/MS-MS	143390-89-0	5.00	ng/L	Sandstrom and others, 2015
Lactofen	Pesticide	NWQL	LC/MS-MS	77501-63-4	10.0	ng/L	Sandstrom and others, 2015
Linuron	Pesticide	NWQL	LC/MS-MS	330-55-2	5.60	ng/L	Sandstrom and others, 2015
Malaoxon	Pesticide	NWQL	LC/MS-MS	1634-78-2	2.40	ng/L	Sandstrom and others, 2015
Malathion	Pesticide	NWQL	LC/MS-MS	121-75-5	5.40	ng/L	Sandstrom and others, 2015
MCPA	Pesticide	NWQL	LC/MS-MS	94-74-6	95.0	ng/L	Sandstrom and others, 2015
Metalaxyl	Pesticide	NWQL	LC/MS-MS	57837-19-1	6.00	ng/L	Sandstrom and others, 2015
Metconazole	Pesticide	NWQL	LC/MS-MS	125116-23-6	5.00	ng/L	Sandstrom and others, 2015
Methamidophos	Pesticide	NWQL	LC/MS-MS	10265-92-6	10.0	ng/L	Sandstrom and others, 2015
Methidathion	Pesticide	NWQL	LC/MS-MS	950-37-8	8.40	ng/L	Sandstrom and others, 2015
Methomyl	Pesticide	NWQL	LC/MS-MS	16752-77-5	3.00	ng/L	Sandstrom and others, 2015
Methomyl oxime	Pesticide	NWQL	LC/MS-MS	13749-94-5	8000	ng/L	Sandstrom and others, 2015
Methoxyfenozide	Pesticide	NWQL	LC/MS-MS	161050-58-4	2.20	ng/L	Sandstrom and others, 2015

Parameter Name	Common/primary use/group	Analyzing laboratory	Method	CAS number	Reporting limit	Units of measurement	Method citation
Methyl paraoxon	Pesticide	NWQL	LC/MS-MS	950-35-6	19.0	ng/L	Sandstrom and others, 2015
Metolachlor	Pesticide	NWQL	LC/MS-MS	51218-45-2	3.20	ng/L	Sandstrom and others, 2015
Metolachlor hydroxy morpholinone	Pesticide	NWQL	LC/MS-MS	61520-54-5	10.0	ng/L	Sandstrom and others, 2015
Metolachlor oxanilic acid	Pesticide	NWQL	LC/MS-MS	152019-73-3	149	ng/L	Sandstrom and others, 2015
Metolachlor sulfonic acid	Pesticide	NWQL	LC/MS-MS	171118-09-5	68.0	ng/L	Sandstrom and others, 2015
Metribuzin	Pesticide	NWQL	LC/MS-MS	21087-64-9	20.0	ng/L	Sandstrom and others, 2015
Metribuzin DK	Pesticide	NWQL	LC/MS-MS	56507-37-0	236	ng/L	Sandstrom and others, 2015
Molinate	Pesticide	NWQL	LC/MS-MS	2212-67-1	28.0	ng/L	Sandstrom and others, 2015
Myclobutanil	Pesticide	NWQL	LC/MS-MS	88671-89-0	7.00	ng/L	Sandstrom and others, 2015
N-(3,4-Dichlorophenyl)-N'-methylurea	Pesticide	NWQL	LC/MS-MS	3567-62-2	5.00	ng/L	Sandstrom and others, 2015
Naled	Pesticide	NWQL	LC/MS-MS	300-76-5	56.0	ng/L	Sandstrom and others, 2015
Nicosulfuron	Pesticide	NWQL	LC/MS-MS	111991-09-4	12.0	ng/L	Sandstrom and others, 2015
Norflurazon	Pesticide	NWQL	LC/MS-MS	27314-13-2	3.40	ng/L	Sandstrom and others, 2015
Novaluron	Pesticide	NWQL	LC/MS-MS	116714-46-6	50.0	ng/L	Sandstrom and others, 2015
O-Ethyl S-methyl S-propyl phosphorodithioate	Pesticide	NWQL	LC/MS-MS	76936-72-6	3.00	ng/L	Sandstrom and others, 2015
O-Ethyl-O-methyl-S-propylphosphorothioate	Pesticide	NWQL	LC/MS-MS	76960-87-7	5.00	ng/L	Sandstrom and others, 2015
O-Ethyl-S-propyl phosphorothioate	Pesticide	NWQL	LC/MS-MS	31110-62-0	64.0	ng/L	Sandstrom and others, 2015

Parameter Name	Common/primary use/group	Analyzing laboratory	Method	CAS number	Reporting limit	Units of measurement	Method citation
Omethoate (Dimethoate oxon)	Pesticide	NWQL	LC/MS-MS	1113-02-6	2.00	ng/L	Sandstrom and others, 2015
Orthosulfamuron	Pesticide	NWQL	LC/MS-MS	213464-77-8	6.00	ng/L	Sandstrom and others, 2015
Oryzalin	Pesticide	NWQL	LC/MS-MS	19044-88-3	12.0	ng/L	Sandstrom and others, 2015
Oxamyl	Pesticide	NWQL	LC/MS-MS	23135-22-0	2.00	ng/L	Sandstrom and others, 2015
Oxamyl oxime	Pesticide	NWQL	LC/MS-MS	30558-43-1	5.00	ng/L	Sandstrom and others, 2015
Oxyfluorfen	Pesticide	NWQL	LC/MS-MS	42874-03-3	500	ng/L	Sandstrom and others, 2015
Paraoxon	Pesticide	NWQL	LC/MS-MS	311-45-5	3.40	ng/L	Sandstrom and others, 2015
Pendimethalin	Pesticide	NWQL	LC/MS-MS	40487-42-1	10.0	ng/L	Sandstrom and others, 2015
Phorate	Pesticide	NWQL	LC/MS-MS	298-02-2	11.0	ng/L	Sandstrom and others, 2015
Phorate oxon	Pesticide	NWQL	LC/MS-MS	2600-69-3	55.0	ng/L	Sandstrom and others, 2015
Phorate oxon sulfone	Pesticide	NWQL	LC/MS-MS	2588-06-9	20.0	ng/L	Sandstrom and others, 2015
Phorate oxon sulfoxide	Pesticide	NWQL	LC/MS-MS	2588-05-8	7.00	ng/L	Sandstrom and others, 2015
Phorate sulfone	Pesticide	NWQL	LC/MS-MS	2588-04-7	36.0	ng/L	Sandstrom and others, 2015
Phorate sulfoxide	Pesticide	NWQL	LC/MS-MS	2588-03-6	4.60	ng/L	Sandstrom and others, 2015
Phthalazinone	Pesticide	NWQL	LC/MS-MS	90004-07-2	15.0	ng/L	Sandstrom and others, 2015
Piperonyl butoxide	Pesticide	NWQL	LC/MS-MS	51-03-6	60.0	ng/L	Sandstrom and others, 2015
Profenofos	Pesticide	NWQL	LC/MS-MS	41198-08-7	3.00	ng/L	Sandstrom and others, 2015
Prometon	Pesticide	NWQL	LC/MS-MS	1610-18-0	4.00	ng/L	Sandstrom and others, 2015

Parameter Name	Common/primary use/group	Analyzing laboratory	Method	CAS number	Reporting limit	Units of measurement	Method citation
Prometryn	Pesticide	NWQL	LC/MS-MS	7287-19-6	4.20	ng/L	Sandstrom and others, 2015
Propanil	Pesticide	NWQL	LC/MS-MS	709-98-8	12.0	ng/L	Sandstrom and others, 2015
Propargite	Pesticide	NWQL	LC/MS-MS	2312-35-8	2.00	ng/L	Sandstrom and others, 2015
Propazine	Pesticide	NWQL	LC/MS-MS	139-40-2	3.20	ng/L	Sandstrom and others, 2015
Propiconazole	Pesticide	NWQL	LC/MS-MS	60207-90-1	6.00	ng/L	Sandstrom and others, 2015
Propoxur	Pesticide	NWQL	LC/MS-MS	114-26-1	3.20	ng/L	Sandstrom and others, 2015
Propyzamide	Pesticide	NWQL	LC/MS-MS	23950-58-5	2.40	ng/L	Sandstrom and others, 2015
Prosulfuron	Pesticide	NWQL	LC/MS-MS	94125-34-5	10.0	ng/L	Sandstrom and others, 2015
Pyraclostrobin	Pesticide	NWQL	LC/MS-MS	175013-18-0	2.40	ng/L	Sandstrom and others, 2015
Pyridaben	Pesticide	NWQL	LC/MS-MS	96489-71-3	2.40	ng/L	Sandstrom and others, 2015
Pyriproxyfen	Pesticide	NWQL	LC/MS-MS	95737-68-1	3.00	ng/L	Sandstrom and others, 2015
sec-Acetochlor oxanilic acid	Pesticide	NWQL	LC/MS-MS	152019-74-4	55.0	ng/L	Sandstrom and others, 2015
Siduron	Pesticide	NWQL	LC/MS-MS	1982-49-6	5.00	ng/L	Sandstrom and others, 2015
Simazine	Pesticide	NWQL	LC/MS-MS	122-34-9	7.20	ng/L	Sandstrom and others, 2015
Sulfentrazone	Pesticide	NWQL	LC/MS-MS	122836-35-5	18.0	ng/L	Sandstrom and others, 2015
Sulfometuron-methyl	Pesticide	NWQL	LC/MS-MS	74222-97-2	4.00	ng/L	Sandstrom and others, 2015
Sulfosulfuron	Pesticide	NWQL	LC/MS-MS	141776-32-1	11.0	ng/L	Sandstrom and others, 2015
Tebuconazole	Pesticide	NWQL	LC/MS-MS	107534-96-3	15.0	ng/L	Sandstrom and others, 2015

Parameter Name	Common/primary use/group	Analyzing laboratory	Method	CAS number	Reporting limit	Units of measurement	Method citation
Tebufenozide	Pesticide	NWQL	LC/MS-MS	112410-23-8	2.00	ng/L	Sandstrom and others, 2015
Tebupirimfos oxon	Pesticide	NWQL	LC/MS-MS	-	2.00	ng/L	Sandstrom and others, 2015
Tebupirimphos	Pesticide	NWQL	LC/MS-MS	96182-53-5	2.00	ng/L	Sandstrom and others, 2015
Tebuthiuron	Pesticide	NWQL	LC/MS-MS	34014-18-1	3.00	ng/L	Sandstrom and others, 2015
Tebuthiuron TP 104	Pesticide	NWQL	LC/MS-MS	59962-53-7	5.60	ng/L	Sandstrom and others, 2015
Tebuthiuron TP 109	Pesticide	NWQL	LC/MS-MS	59962-54-8	11.0	ng/L	Sandstrom and others, 2015
Tebuthiuron TP 109 (OH)	Pesticide	NWQL	LC/MS-MS	139888-73-6	38.0	ng/L	Sandstrom and others, 2015
Tebuthiuron TP 108	Pesticide	NWQL	LC/MS-MS	39222-73-6	10.0	ng/L	Sandstrom and others, 2015
Terbacil	Pesticide	NWQL	LC/MS-MS	5902-51-2	21.0	ng/L	Sandstrom and others, 2015
Terbufos	Pesticide	NWQL	LC/MS-MS	13071-79-9	6.80	ng/L	Sandstrom and others, 2015
Terbufos oxon	Pesticide	NWQL	LC/MS-MS	56070-14-5	4.00	ng/L	Sandstrom and others, 2015
Terbufos oxon sulfone	Pesticide	NWQL	LC/MS-MS	56070-15-6	11.0	ng/L	Sandstrom and others, 2015
Terbufos oxon sulfoxide	Pesticide	NWQL	LC/MS-MS	56165-57-2	4.00	ng/L	Sandstrom and others, 2015
Terbufos sulfone	Pesticide	NWQL	LC/MS-MS	56070-16-7	11.0	ng/L	Sandstrom and others, 2015
Terbufos sulfoxide	Pesticide	NWQL	LC/MS-MS	10548-10-4	3.00	ng/L	Sandstrom and others, 2015
Terbuthylazine	Pesticide	NWQL	LC/MS-MS	5915-41-3	3.60	ng/L	Sandstrom and others, 2015
Tetraconazole	Pesticide	NWQL	LC/MS-MS	112281-77-3	7.00	ng/L	Sandstrom and others, 2015
Thiobencarb	Pesticide	NWQL	LC/MS-MS	28249-77-6	4.20	ng/L	Sandstrom and others, 2015

Parameter Name	Common/primary use/group	Analyzing laboratory	Method	CAS number	Reporting limit	Units of measurement	Method citation
trans-Permethrin	Pesticide	NWQL	LC/MS-MS	61949-77-7	3.80	ng/L	Sandstrom and others, 2015
Triallate	Pesticide	NWQL	LC/MS-MS	-	12.0	ng/L	Sandstrom and others, 2015
Tribufos	Pesticide	NWQL	LC/MS-MS	78-48-8	2.00	ng/L	Sandstrom and others, 2015
Triclopyr	Pesticide	NWQL	LC/MS-MS	55335-06-3	88.0	ng/L	Sandstrom and others, 2015
Trifloxystrobin	Pesticide	NWQL	LC/MS-MS	141517-21-7	2.80	ng/L	Sandstrom and others, 2015
1,1,1-Trichloro-2-propanone	DBP/Propanones	OCRL	GC-MS	918-00-3	0.500	ug/L	Hladik and others, 2014
1,1-Dichloro-2-propanone	DBP/Propanones	OCRL	GC-MS	513-88-2	0.500	ug/L	Hladik and others, 2014
Bromoacetonitrile	DBP/Haloacetonitriles	OCRL	GC-MS	590-17-0	0.100	ug/L	Hladik and others, 2014
Bromochloroacetonitrile	DBP/Haloacetonitriles	OCRL	GC-MS	83463-62-1	0.500	ug/L	Hladik and others, 2014
Bromochloroiodomethane	DBP/Trihalomethanes	OCRL	GC-MS	34970-00-8	0.050	ug/L	Hladik and others, 2014
Bromochloronitromethane	DBP/Halonitromethanes	OCRL	GC-MS	135531-25-8	1.00	ug/L	Hladik and others, 2014
Bromodichloromethane	DBP/Trihalomethanes	OCRL	GC-MS	75-27-4	0.050	ug/L	Hladik and others, 2014
Bromodiiodomethane	DBP/Trihalomethanes	OCRL	GC-MS	557-95-9	0.050	ug/L	Hladik and others, 2014
Bromoform (tribromomethane)	DBP/Trihalomethanes	OCRL	GC-MS	75-25-2	0.050	ug/L	Hladik and others, 2014
Bromonitromethane	DBP/Halonitromethanes	OCRL	GC-MS	563-70-2	1.00	ug/L	Hladik and others, 2014
Bromopicrin (Tribromonitromethane)	DBP/Halonitromethanes	OCRL	GC-MS	464-10-8	1.00	ug/L	Hladik and others, 2014
Chlorodiiodomethane	DBP/Trihalomethanes	OCRL	GC-MS	593-71-5	0.050	ug/L	Hladik and others, 2014
Chloroform (trichloromethane)	DBP/Trihalomethanes	OCRL	GC-MS	67-66-3	0.050	ug/L	Hladik and others, 2014
Chloropicrin (Trichloronitromethane)	DBP/Halonitromethanes	OCRL	GC-MS	76-06-2	1.00	ug/L	Hladik and others, 2014

Parameter Name	Common/primary use/group	Analyzing laboratory	Method	CAS number	Reporting limit	Units of measurement	Method citation
Dibromoacetonitrile	DBP/Haloacetonitriles	OCRL	GC-MS	3252-43-5	1.00	ug/L	Hladik and others, 2014
Dibromochloromethane	DBP/Trihalomethanes	OCRL	GC-MS	124-48-1	0.050	ug/L	Hladik and others, 2014
Dibromoiodomethane	DBP/Trihalomethanes	OCRL	GC-MS	563-94-2	0.050	ug/L	Hladik and others, 2014
Dichloroacetonitrile	DBP/Haloacetonitriles	OCRL	GC-MS	107-14-2	0.100	ug/L	Hladik and others, 2014
Dichloroiodomethane	DBP/Trihalomethanes	OCRL	GC-MS	594-04-7	0.050	ug/L	Hladik and others, 2014
Dichloronitromethane	DBP/Halonitromethanes	OCRL	GC-MS	7119-89-3	0.100	ug/L	Hladik and others, 2014
Iodoform (triiodomethane)	DBP/Trihalomethanes	OCRL	GC-MS	75-47-8	0.100	ug/L	Hladik and others, 2014
Trichloroacetonitrile	DBP/Haloacetonitriles	OCRL	GC-MS	545-06-2	0.100	ug/L	Hladik and others, 2014
pH	Field measurement	Field personnel	Multi-parameter meter	-	0.01	std units	National Field Manual, variously dated
Specific conductance	Field measurement	Field personnel	Multi-parameter meter	-	5.00	µS/cm	National Field Manual, variously dated
Temperature	Field measurement	Field personnel	Multi-parameter meter	-	0.100	degrees C	National Field Manual, variously dated
Alkalinity	Alkalinity	RCL	Titration	-	2	mg/L	Barringer and Johnsson, 1996 and Fishman and Friedman, 1989
Aluminum	Trace element	RCL	ICP-OES	7429-90-5	0.001	mg/L	EPA, 2014
Antimony	Trace element	RCL	ICP-OES	7440-36-0	0.040	mg/L	EPA, 2014
Arsenic	Trace element	RCL	ICP-OES	7440-38-2	1.00	ug/L	EPA, 2014
Barium	Trace element	RCL	ICP-OES	7440-39-3	0.0005	mg/L	EPA, 2014
Beryllium	Trace element	RCL	ICP-OES	7440-41-7	0.0005	mg/L	EPA, 2014
Boron	Trace element	RCL	ICP-OES	7440-42-8	0.006	mg/L	EPA, 2014
Bromide	Trace element	RCL	ICP-OES	24959-67-9	0.010	mg/L	EPA, 2014
Cadmium	Trace element	RCL	ICP-OES	7440-43-9	0.003	mg/L	EPA, 2014
Calcium	Cation	RCL	ICP-OES	7440-70-2	0.020	mg/L	EPA, 2014
Chloride	Anion	RCL	IC	16887-00-6	0.050	mg/L	EPA, 2013
Chromium	Trace element	RCL	ICP-OES	7440-47-3	0.200	ug/L	EPA, 2014
Cobalt	Trace element	RCL	ICP-OES	7440-48-4	0.003	mg/L	EPA, 2014
Copper	Trace element	RCL	ICP-OES	7440-50-8	0.001	mg/L	EPA, 2014
Fluoride	Trace element	RCL	ICP-OES	16984-48-8	0.050	mg/L	EPA, 2014
Hexavalent chromium	Trace element	RCL	Cation exchange	18540-29-9	0.200	ug/L	Ball and McCleskey, 2003
Iron	Trace element	RCL	ICP-OES	7439-89-6	0.001	mg/L	EPA, 2014

Parameter Name	Common/primary use/group	Analyzing laboratory	Method	CAS number	Reporting limit	Units of measurement	Method citation
Lead	Trace element	RCL	ICP-OES	7439-92-1	0.500	ug/L	Hergenreder, 2011
Lithium	Trace element	RCL	ICP-OES	7439-93-2	0.0005	mg/L	EPA, 2014
Magnesium	Cation	RCL	ICP-OES	7439-95-4	0.003	mg/L	EPA, 2014
Manganese	Trace element	RCL	ICP-OES	7439-96-5	0.001	mg/L	EPA, 2014
Molybdemum	Trace element	RCL	ICP-OES	7439-98-7	0.005	mg/L	EPA, 2014
Nickel	Trace element	RCL	ICP-OES	7440-02-0	0.002	mg/L	EPA, 2014
Potassium	Cation	RCL	ICP-OES	7440-09-7	0.003	mg/L	EPA, 2014
Selenium	Trace element	RCL	AAS	7782-49-2	2.00	ug/l	PerkinElmer, 2011
Silica	Anion	RCL	ICP-OES	-	0.010	mg/L	EPA, 2014
Sodium	Cation	RCL	ICP-OES	7440-23-5	0.040	mg/L	EPA, 2014
Strontium	Trace element	RCL	ICP-OES	7440-24-6	0.0005	mg/L	EPA, 2014
Sulfate	Anion	RCL	IC	18785-72-3	0.100	mg/L	EPA, 2013
Uranium	Trace element	RCL	ICP-OES	7440-61-1	0.004	mg/L	EPA, 2014
Vanadium	Trace element	RCL	ICP-OES	7440-62-2	0.001	mg/L	EPA, 2014
Zinc	Trace element	RCL	ICP-OES	7440-66-6	0.001	mg/L	EPA, 2014
Nitrate + Nitrite	Nutrient	NWQL	Enzyme reduction	-	0.04	mg/L as N	Patton and Kryskalla, 2011
Nitrate	Nutrient	RCL	ICP-OES	-	0.050	mg/L	EPA, 2014
Phosphorus	Nutrient	RCL	IC	7723-14-0	0.050	mg/L	EPA, 2013
1H,1H,2H,2H-Perfluorohexane sulfonate (4:2) (4:2 FTS)	PFAS	NWQL	LC/MS-MS	757124-72-4	4.68	ng/L	Kolpin and others, 2021, Supporting Information
1H,1H,2H,2H-Perfluorohexane sulfonate (6:2) (6:2 FTS)	PFAS	NWQL	LC/MS-MS	27619-97-2	9.50	ng/L	Kolpin and others, 2021, Supporting Information
1H,1H,2H,2H-Perfluorohexane sulfonate (8:2) (8:2 FTS)	PFAS	NWQL	LC/MS-MS	39108-34-4	24.0	ng/L	Kolpin and others, 2021, Supporting Information
9-Chlorohexadecafluoro-3-oxanonane-1-sulfonate (9Cl-PF3ONS)	PFAS	NWQL	LC/MS-MS	756426-58-1	25.0	ng/L	Kolpin and others, 2021, Supporting Information

Parameter Name	Common/primary use/group	Analyzing laboratory	Method	CAS number	Reporting limit	Units of measurement	Method citation
Perfluorobutane sulfonamide (FBSA)	PFAS	NWQL	LC/MS-MS	30334-69-1	50.0	ng/L	Kolpin and others, 2021, Supporting Information
Perfluoro-1-hexanesulfonamide (FHx-SA)	PFAS	NWQL	LC/MS-MS	41997-13-1	10.0	ng/L	Kolpin and others, 2021, Supporting Information
Perfluoro-1-octanesulfonamide (FOSA)	PFAS	NWQL	LC/MS-MS	754-91-6	1.00	ng/L	Kolpin and others, 2021, Supporting Information
Hexafluoropropylene oxide dimer acid (HFPO-DA)(GenX)	PFAS	NWQL	LC/MS-MS	13252-13-6	2.50	ng/L	Kolpin and others, 2021, Supporting Information
4,8-Dioxa-3H-perfluorononanoate (NaDONA, ADONA)	PFAS	NWQL	LC/MS-MS	919005-14-4	1.00	ng/L	Kolpin and others, 2021, Supporting Information
N-ethyl perfluorooctanesulfonamidoacetate (N-EtFOSAA)	PFAS	NWQL	LC/MS-MS	2991-50-6	5.00	ng/L	Kolpin and others, 2021, Supporting Information
N-Ethylperfluorooctane-1-sulfonamide (N-EtFOSA-M)	PFAS	NWQL	LC/MS-MS	4151-50-2	10.1	ng/L	Kolpin and others, 2021, Supporting Information
N-methylperfluorooctane sulfonamidoacetate (N-MeFOSAA)	PFAS	NWQL	LC/MS-MS	2355-31-9	10.0	ng/L	Kolpin and others, 2021, Supporting Information
Perfluorobutyrate (PFBA)	PFAS	NWQL	LC/MS-MS	375-22-4	5.00	ng/L	Kolpin and others, 2021, Supporting Information
Perfluoro-1-butanesulfonate (PFBS)	PFAS	NWQL	LC/MS-MS	375-73-5	2.21	ng/L	Kolpin and others, 2021, Supporting Information
Perfluoro-n-decanoate (PFDA)	PFAS	NWQL	LC/MS-MS	83-89-6	5.00	ng/L	Kolpin and others, 2021, Supporting Information
Perfluorododecanoate (PFDoDA)	PFAS	NWQL	LC/MS-MS	307-55-14	25.0	ng/L	Kolpin and others, 2021, Supporting Information

Parameter Name	Common/primary use/group	Analyzing laboratory	Method	CAS number	Reporting limit	Units of measurement	Method citation
Perfluoro-1-decanesulfonate (PFDS)	PFAS	NWQL	LC/MS-MS	335-77-3	9.65	ng/L	Kolpin and others, 2021, Supporting Information
Perfluoro-n-heptanoate (PFHpA)	PFAS	NWQL	LC/MS-MS	375-85-9	5.00	ng/L	Kolpin and others, 2021, Supporting Information
Perfluoro-1-heptanesulfonate (PFHpS)	PFAS	NWQL	LC/MS-MS	375-92-8	4.75	ng/L	Kolpin and others, 2021, Supporting Information
Perfluoro-n-hexanoate (PFHxA)	PFAS	NWQL	LC/MS-MS	307-24-4	10.0	ng/L	Kolpin and others, 2021, Supporting Information
Perfluoro-1-hexanesulfonate (PFHxS)(linear)	PFAS	NWQL	LC/MS-MS	355-46-4	1.85-4.40	ng/L	Kolpin and others, 2021, Supporting Information
Perfluoro-1-hexanesulfonate (PFHxS)(branched)	PFAS	NWQL	LC/MS-MS	355-46-4	2.28-4.67	ng/L	Kolpin and others, 2021, Supporting Information
Perfluoro-n-nonanoate (PFNA)	PFAS	NWQL	LC/MS-MS	375-95-1	5.00-8.00	ng/L	Kolpin and others, 2021, Supporting Information
Perfluoro-1-nonanesulfonate (PFNS)	PFAS	NWQL	LC/MS-MS	68259-12-1	2.40-2.70	ng/L	Kolpin and others, 2021, Supporting Information
Perfluoro-n-octanoate (PFOA)	PFAS	NWQL	LC/MS-MS	335-67-1	2.50	ng/L	Kolpin and others, 2021, Supporting Information
Perfluorooctanesulfonate (PFOS)(linear)	PFAS	NWQL	LC/MS-MS	1763-23-1	18.3	ng/L	Kolpin and others, 2021, Supporting Information
Perfluorooctanesulfonate (PFOS)(branched)	PFAS	NWQL	LC/MS-MS	1763-23-1	9.26	ng/L	Kolpin and others, 2021, Supporting Information
Perfluoro-n-pentanoate (PFPeA)	PFAS	NWQL	LC/MS-MS	2706-90-3	10.0	ng/L	Kolpin and others, 2021, Supporting Information
Perfluoro-1-pentanesulfonate (PFPeS)	PFAS	NWQL	LC/MS-MS	2706-91-4	2.35	ng/L	Kolpin and others, 2021, Supporting Information
Perfluoro-n-tetradecanoate (PFTeDA)	PFAS	NWQL	LC/MS-MS	376-06-7	10.1	ng/L	Kolpin and others, 2021, Supporting Information

Parameter Name	Common/primary use/group	Analyzing laboratory	Method	CAS number	Reporting limit	Units of measurement	Method citation
Perfluoro-n-tridecanoate (PFTrDA)	PFAS	NWQL	LC/MS-MS	72629-94-8	10.1	ng/L	Kolpin and others, 2021, Supporting Information
Perfluoro-n-undecanoate (PFUnDA)	PFAS	NWQL	LC/MS-MS	2058-94-8	10.0	ng/L	Kolpin and others, 2021, Supporting Information
1,1,1,2-Tetrachloroethane	Volatile organic compound	NWQL	GC/MS	630-20-6	0.04	ug/L	Rose and others, 2016
1,1,1-Trichloroethane	Volatile organic compound	NWQL	GC/MS	71-55-6	0.03	ug/L	Rose and others, 2016
1,1,2-Trichloroethane	Volatile organic compound	NWQL	GC/MS	79-00-5	0.046	ug/L	Rose and others, 2016
1,1-Dichloroethane	Volatile organic compound	NWQL	GC/MS	75-34-3	0.044	ug/L	Rose and others, 2016
1,1-Dichloroethene	Volatile organic compound	NWQL	GC/MS	75-35-4	0.025	ug/L	Rose and others, 2016
1,1-Difluoroethane	Volatile organic compound	NWQL	GC/MS	75-37-6	0.025	ug/L	Rose and others, 2016
1,2,3,4-Tetrahydronapthalene	Volatile organic compound	NWQL	GC/MS	119-64-2	0.08	ug/L	Rose and others, 2016
1,2,4-Trichlorobenzene	Volatile organic compound	NWQL	GC/MS	120-82-1	0.08	ug/L	Rose and others, 2016
1,2,4-Trimethylbenzene	Volatile organic compound	NWQL	GC/MS	95-63-6	0.032	ug/L	Rose and others, 2016
1,2-Dichloro-1,1,2,2-tetrafluoroethane	Volatile organic compound	NWQL	GC/MS	76-14-2	0.03	ug/L	Rose and others, 2016
1,2-Dichlorobenzene	Volatile organic compound	NWQL	GC/MS	95-50-1	0.028	ug/L	Rose and others, 2016
1,2-Dichloroethane	Volatile organic compound	NWQL	GC/MS	107-06-2	0.08	ug/L	Rose and others, 2016
1,3-Butadiene	Volatile organic compound	NWQL	GC/MS	106-99-0	0.08	ug/L	Rose and others, 2016
1,4-Dichlorobenzene	Volatile organic compound	NWQL	GC/MS	106-46-7	0.026	ug/L	Rose and others, 2016
1-Chloro-1,1-difluoroethane	Volatile organic compound	NWQL	GC/MS	75-68-3	0.08	ug/L	Rose and others, 2016

Parameter Name	Common/primary use/group	Analyzing laboratory	Method	CAS number	Reporting limit	Units of measurement	Method citation
2,2-Dichloro-1,1,1-trifluoroethane	Volatile organic compound	NWQL	GC/MS	306-83-2	0.025	ug/L	Rose and others, 2016
Benzene	Volatile organic compound	NWQL	GC/MS	71-43-2	0.026	ug/L	Rose and others, 2016
Bromochloromethane	Volatile organic compound	NWQL	GC/MS	74-97-5	0.06	ug/L	Rose and others, 2016
Bromodichloromethane	Volatile organic compound	NWQL	GC/MS	75-27-4	0.034	ug/L	Rose and others, 2016
Bromomethane	Volatile organic compound	NWQL	GC/MS	74-83-9	0.2	ug/L	Rose and others, 2016
Butane	Volatile organic compound	NWQL	GC/MS	106-97-8	0.08	ug/L	Rose and others, 2016
Carbon disulfide	Volatile organic compound	NWQL	GC/MS	75-15-0	0.1	ug/L	Rose and others, 2016
Chlorobenzene	Volatile organic compound	NWQL	GC/MS	108-90-7	0.026	ug/L	Rose and others, 2016
Chlorodifluoromethane	Volatile organic compound	NWQL	GC/MS	75-45-6	0.04	ug/L	Rose and others, 2016
Chloromethane	Volatile organic compound	NWQL	GC/MS	74-87-3	0.2	ug/L	Rose and others, 2016
cis-1,2-Dichloroethene	Volatile organic compound	NWQL	GC/MS	156-59-2	0.025	ug/L	Rose and others, 2016
cis-1,3-Dichloropropene	Volatile organic compound	NWQL	GC/MS	10061-01-5	0.1	ug/L	Rose and others, 2016
Dibromochloromethane	Volatile organic compound	NWQL	GC/MS	124-48-1	0.12	ug/L	Rose and others, 2016
Dichlorofluoromethane	Volatile organic compound	NWQL	GC/MS	75-43-4	0.05	ug/L	Rose and others, 2016
Dichloromethane	Volatile organic compound	NWQL	GC/MS	75-09-2	0.04	ug/L	Rose and others, 2016
Ethylbenzene	Volatile organic compound	NWQL	GC/MS	100-41-4	0.036	ug/L	Rose and others, 2016
Hexane	Volatile organic compound	NWQL	GC/MS	110-54-3	0.068	ug/L	Rose and others, 2016
m- and p-Xylene	Volatile organic compound	NWQL	GC/MS	179601-23-1	0.08	ug/L	Rose and others, 2016

Parameter Name	Common/primary use/group	Analyzing laboratory	Method	CAS number	Reporting limit	Units of measurement	Method citation
Methyl tert-butyl ether	Volatile organic compound	NWQL	GC/MS	1634-04-4	0.1	ug/L	Rose and others, 2016
n-Pentane	Volatile organic compound	NWQL	GC/MS	109-66-0	0.066	ug/L	Rose and others, 2016
n-Propylbenzene	Volatile organic compound	NWQL	GC/MS	103-65-1	0.036	ug/L	Rose and others, 2016
Naphthalene	Volatile organic compound	NWQL	GC/MS	91-20-3	0.26	ug/L	Rose and others, 2016
o-Xylene	Volatile organic compound	NWQL	GC/MS	95-47-6	0.032	ug/L	Rose and others, 2016
sec-Butylbenzene	Volatile organic compound	NWQL	GC/MS	135-98-8	0.034	ug/L	Rose and others, 2016
Styrene	Volatile organic compound	NWQL	GC/MS	100-42-5	0.042	ug/L	Rose and others, 2016
Tetrachloroethylene	Volatile organic compound	NWQL	GC/MS	127-18-4	0.058	ug/L	Rose and others, 2016
Tetrachloromethane	Volatile organic compound	NWQL	GC/MS	56-23-5	0.06	ug/L	Rose and others, 2016
Toluene	Volatile organic compound	NWQL	GC/MS	108-88-3	0.2	ug/L	Rose and others, 2016
trans-1,2-Dichloroethylene	Volatile organic compound	NWQL	GC/MS	156-60-5	0.025	ug/L	Rose and others, 2016
trans-1,3-Dichloropropene	Volatile organic compound	NWQL	GC/MS	10061-02-6	0.1	ug/L	Rose and others, 2016
Tribromomethane	Volatile organic compound	NWQL	GC/MS	75-25-2	0.14	ug/L	Rose and others, 2016
Trichloroethylene	Volatile organic compound	NWQL	GC/MS	79-01-6	0.025	ug/L	Rose and others, 2016
Trichloromethane	Volatile organic compound	NWQL	GC/MS	67-66-3	0.03	ug/L	Rose and others, 2016
Vinyl chloride	Volatile organic compound	NWQL	GC/MS	75-01-4	0.06	ug/L	Rose and others, 2016
1,1-Dichloro-2-propanone	Volatile organic compound	NWQL	GC/MS	513-88-2	0.24	ug/L	Rose and others, 2016
1,2,3-Trichloropropane	Volatile organic compound	NWQL	GC/MS	96-18-4	0.006	ug/L	Rose and others, 2016

Parameter Name	Common/primary use/group	Analyzing laboratory	Method	CAS number	Reporting limit	Units of measurement	Method citation
1,2-Dibromo-3-chloropropane	Volatile organic compound	NWQL	GC/MS	96-12-8	0.020	ug/L	Rose and others, 2016
1,2-Dibromoethane	Volatile organic compound	NWQL	GC/MS	106-93-4	0.004	ug/L	Rose and others, 2016
1,2-Dichloropropane	Volatile organic compound	NWQL	GC/MS	78-87-5	0.004	ug/L	Rose and others, 2016
1,3-Dioxolane	Volatile organic compound	NWQL	GC/MS	646-06-0	0.38	ug/L	Rose and others, 2016
1,4-Dioxane	Volatile organic compound	NWQL	GC/MS	123-91-1	0.2	ug/L	Rose and others, 2016
1-octanol	Volatile organic compound	NWQL	GC/MS	111-87-5	1.8	ug/L	Rose and others, 2016
1Methoxy-4(2-propenyl)benzene	Volatile organic compound	NWQL	GC/MS	140-67-0	0.92	ug/L	Rose and others, 2016
2-ethoxyethyl acetate	Volatile organic compound	NWQL	GC/MS	111-15-9	5.4	ug/L	Rose and others, 2016
2-hexanone, 5-methyl-	Volatile organic compound	NWQL	GC/MS	110-12-3	0.022	ug/L	Rose and others, 2016
2-Nitropropane	Volatile organic compound	NWQL	GC/MS	79-46-9	0.12	ug/L	Rose and others, 2016
2-pentanol, 4-methyl-	Volatile organic compound	NWQL	GC/MS	108-11-2	0.2	ug/L	Rose and others, 2016
2-Propen-1-ol	Volatile organic compound	NWQL	GC/MS	107-18-6	7.8	ug/L	Rose and others, 2016
4-heptanone, 2,6-dimethyl-	Volatile organic compound	NWQL	GC/MS	108-83-8	0.032	ug/L	Rose and others, 2016
acetate, ethyl	Volatile organic compound	NWQL	GC/MS	141-78-6	0.06	ug/L	Rose and others, 2016
acetate, isobutyl	Volatile organic compound	NWQL	GC/MS	110-19-0	0.028	ug/L	Rose and others, 2016
acetate, isopropyl	Volatile organic compound	NWQL	GC/MS	108-21-4	0.01	ug/L	Rose and others, 2016
acetate, propyl	Volatile organic compound	NWQL	GC/MS	109-60-4	0.032	ug/L	Rose and others, 2016
acetonitrile	Volatile organic compound	NWQL	GC/MS	75-05-8	0.8	ug/L	Rose and others, 2016

Parameter Name	Common/primary use/group	Analyzing laboratory	Method	CAS number	Reporting limit	Units of measurement	Method citation
alpha-Terpineol	Volatile organic compound	NWQL	GC/MS	98-55-5	2.2	ug/L	Rose and others, 2016
Butanal	Volatile organic compound	NWQL	GC/MS	123-72-8	0.38	ug/L	Rose and others, 2016
butanol	Volatile organic compound	NWQL	GC/MS	71-36-3	0.8	ug/L	Rose and others, 2016
Chloropicrin	Volatile organic compound	NWQL	GC/MS	76-06-2	0.14	ug/L	Rose and others, 2016
cyclohexanone	Volatile organic compound	NWQL	GC/MS	108-94-1	1.2	ug/L	Rose and others, 2016
diethylamine, n-nitroso-	Volatile organic compound	NWQL	GC/MS	55-18-5	6.2	ug/L	Rose and others, 2016
Dimethoxymethane	Volatile organic compound	NWQL	GC/MS	109-87-5	0.044	ug/L	Rose and others, 2016
Hexachlorocyclopentadiene	Volatile organic compound	NWQL	GC/MS	77-47-4	10	ug/L	Rose and others, 2016
hexanol, 2-ethyl-	Volatile organic compound	NWQL	GC/MS	104-76-7	4.4	ug/L	Rose and others, 2016
Isophorone	Volatile organic compound	NWQL	GC/MS	78-59-1	2.4	ug/L	Rose and others, 2016
isopropyl alcohol	Volatile organic compound	NWQL	GC/MS	67-63-0	0.6	ug/L	Rose and others, 2016
Methyl acetate	Volatile organic compound	NWQL	GC/MS	79-20-9	0.14	ug/L	Rose and others, 2016
Methyl tert-butyl ether	Volatile organic compound	NWQL	GC/MS	1634-04-4	0.012	ug/L	Rose and others, 2016
n-Pentanal	Volatile organic compound	NWQL	GC/MS	110-62-3	0.054	ug/L	Rose and others, 2016
Nitrobenzene	Volatile organic compound	NWQL	GC/MS	98-95-3	1.4	ug/L	Rose and others, 2016
tert-Butyl alcohol	Volatile organic compound	NWQL	GC/MS	75-65-0	0.24	ug/L	Rose and others, 2016
trans-Crotonaldehyde	Volatile organic compound	NWQL	GC/MS	123-73-9	2.6	ug/L	Rose and others, 2016
Microcystins	Cyanotoxins	OGRL	ELISA	101043-37-2	0.100	ug/L	Loftin and others, 2016
Saxitoxins	Cyanotoxins	OGRL	ELISA	35523-89-8	0.02	ug/L	Loftin and others, 2016
Cylindrospermopsin	Cyanotoxins	OGRL	ELISA	143545-90-8	0.05	ug/L	Loftin and others, 2016

Parameter Name	Common/primary use/group	Analyzing laboratory	Method	CAS number	Reporting limit	Units of measurement	Method citation
Anatoxin-A	Cyanotoxins	OGRL	ELISA	64285-06-9	0.15	ug/L	Loftin and others, 2016
17B-Estradiol (E2)	Estrogen receptor agonists	EPA, ORD	T47KBlu	-	0.028	ng/L (equivalent)	Conley and others, 2017
4,5a-Dihydrotestosterone (DHT)	Androgen receptor agonists	EPA, ORD	CV1-chAR	-	0.74	ng/L (equivalent)	Conley and others, 2017
Dexamethasone (DEX)	Glucocorticoid receptor agonists	EPA, ORD	CV1-hGR	-	6.59	ng/L (equivalent)	Conley and others, 2017

Table S3. Summary data for detected organic compounds, in micrograms per liter, analyzed in source water, finished water, and service-area tapwater samples collected for the U.S. Geological Survey (USGS) Ecosystems Mission Area Environmental Health Infrastructure Project, Minnesota Tapwater Exposure Study, 2019. Also included are any exceedances of health advisories. For data on all organic compounds detected please see Romanok et al., 2023 (https://doi.org/10.5066/P9YQ24QW)

[MCL, Maximum Contaminant Level set by the U.S. Environmental Protection Agency; MCLG, Maximum Contaminant Level Goal, maximum level of a contaminant in drinking water at which no known or anticipated adverse effect on the health of persons would occur, allowing an adequate margin of safety. MCLG are non-enforceable public health goals. MCL and MCLG are available at https://www.epa.gov/dwregdev/how-epa-regulates-drinking-water-contaminants. WHO, World Health Organization drinking water guidelines. HA exceedances, number of exceedances of health advisories, including MCLG.]

Site code	Site type	Source of water	SHA exceedances (MCL)	SHA exceedances (MCLG)	Sum Organics	Count Organics	Sum DBP	Count DBP	Sum Pest	Count Pest	Sum Pharm	Count Pharm	Sum VOC	Count VOC	Sum PFAS	Count PFAS
MNTW2019 023	Source	Surface water	**0**	**1**	1.9	14	1.6	3	0.013	3	0.003	1	0.183	3	0.041	3
MNTW2019 022	Finished	Surface water	**0**	**2**	13.7	8	13.7	7	0	0	0	0	0	0	0.015	1
MNTW2019 025	Tap	Surface water	**0**	**2**	15.4	9	14.8	6	0	0	0	0	0.627	2	0.014	1
MNTW2019 024	Tap	Surface water	**0**	**2**	33.9	18	31.6	8	0.020	1	0	0	2.28	8	0.014	1
MNTW2019 010	Source	Groundwater	**0**	**1**	0.2	5	0	0	0	0	0	0	0.138	3	0.021	2
MNTW2019 009	Finished	Groundwater	**0**	**3**	15.6	12	15.5	8	0	0	0	0	0.107	3	0.018	1
MNTW2019 007	Tap	Groundwater	**0**	**3**	4.7	20	3.0	8	0.033	3	0.004	1	1.66	7	0.011	1
MNTW2019 008	Tap	Groundwater	**0**	**3**	4.0	14	3.2	7	0	0	0.033	1	0.743	5	0.013	1
MNTW2019 027	Source	Groundwater	**0**	**1**	2.7	23	0.10	1	1.99	14	0	0	0.170	3	0.461	5
MNTW2019 026	Finished	Groundwater	**0**	**3**	4.7	25	2.4	4	1.76	14	0	0	0.175	3	0.347	4

Site code	Site type	Source of water	SHA exceedances (MCL)	SHA exceedances (MCLG)	Sum Organics	Count Organics	Sum DBP	Count DBP	Sum Pest	Count Pest	Sum Pharm	Count Pharm	Sum VOC	Count VOC	Sum PFAS	Count PFAS
MNTW2019 030	Tap	Groundwater	**0**	**4**	5.0	25	3.8	5	0.839	11	0.014	1	0.156	5	0.239	3
MNTW2019 028	Source	Groundwater	**0**	**1**	2.1	24	0	0	1.88	18	0.012	2	0.113	2	0.145	2
MNTW2019 029	Finished	Groundwater	**0**	**1**	2.25	22	0	0	1.94	15	0.005	2	0.140	3	0.167	2
MNTW2019 031	Tap	Groundwater	**0**	**3**	2.93	24	0.400	4	2.29	14	0.004	2	0.073	1	0.166	3
MNTW2019 037	Source	Groundwater	**0**	**0**	2.34	6	0	0	0	0	0	0	2.33	5	0.013	1
MNTW2019 036	Finished	Groundwater	**0**	**3**	12.0	19	11.4	8	0.023	3	0	0	0.576	7	0.016	1
MNTW2019 038	Tap	Groundwater	**0**	**3**	19.5	17	18.6	10	0.011	2	0	0	0.882	3	0.016	2
MNTW2019 039	Tap	Groundwater	**0**	**5**	19.0	18	18.4	10	0.005	2	0	0	0.570	5	0.014	1
MNTW2019 011	Source	Surface water	**0**	**1**	97.0	33	0	0	0.463	14	0.175	9	95.8	8	0.011	1
MNTW2019 012	Finished	Surface water	**0**	**1**	27.3	12	27.0	7	0.099	2	0	0	0.200	2	0.009	1
MNTW2019 013	Tap	Surface water	**0**	**2**	22.7	14	22.4	8	0.013	1	0	0	0.222	4	0.009	1
MNTW2019 014	Tap	Surface water	**0**	**1**	21.8	14	21.6	8	0.028	2	0	0	0.181	3	0.008	1
MNTW2019 020	Source	Groundwater	**0**	**0**	1.14	10	0.100	1	0.993	6	0	0	0.030	1	0.014	2
MNTW2019 019	Finished	Groundwater	**0**	**2**	9.53	13	8.30	5	1.216	7	0	0	0	0	0.013	1
MNTW2019 006	Tap	Groundwater	**0**	**2**	9.61	16	8.72	6	0.768	6	0.031	1	0.078	2	0.010	1
MNTW2019 018	Source	Groundwater	**0**	**0**	0.826	9	0	0	0.705	7	0	0	0.107	1	0.0145	1
MNTW2019 017	Finished	Groundwater	**0**	**2**	7.90	16	7.10	4	0.678	8	0	0	0.105	2	0.0145	2
MNTW2019 021	Tap	Groundwater	**0**	**2**	14.6	21	13.2	7	1.021	8	0	0	0.331	4	0.0122	2
MNTW2019 002	Source	Groundwater	**0**	**1**	0.245	11	0	0	0.071	5	0	0	0.030	2	0.1438	4
MNTW2019 005	Tap	Groundwater	**0**	**0**	0.094	3	0	0	0	0	0	0	0.068	1	0.0261	2
MNTW2019 004	Source	Surface water	**0**	**0**	3.15	17	0	0	0.307	12	0	0	1.33	3	0.0174	1
MNTW2019 003	Finished	Surface water	**0**	**2**	37.0	24	36.7	9	0.213	9	0	0	0.112	3	0.0230	3
MNTW2019 001	Tap	Surface water	**0**	**2**	36.8	25	36.4	9	0.350	13	0	0	0	0	0.0266	3
MNTW2019 016	Source	Groundwater	**0**	**0**	0.180	3	0	0	0.017	1	0.009	1	0	0	0.1547	1
MNTW2019 015	Tap	Groundwater	**0**	**0**	0.164	2	0	0	0	0	0.010	1	0	0	0.1540	1
MNTW2019 033	Source	Groundwater	**0**	**0**	0.639	12	0	0	0.385	9	0	0	0	0	0.2537	3
MNTW2019 032	Finished	Groundwater	**0**	**0**	1.70	15	0	0	0.866	9	0	0	0.600	1	0.2312	5
MNTW2019 034	Tap	Groundwater	**0**	**2**	10.0	27	8.66	8	0.304	10	0	0	0.773	5	0.2370	4
MNTW2019 035	Tap	Groundwater	**0**	**2**	10.1	22	9.07	7	0.642	9	0	0	0.185	4	0.2018	2

Table S4. Subset of detected trace element and ion concentrations (in units shown) in source water, finished water, and service-area tapwater samples collected for the U.S. Geological Survey (USGS) Ecosystems Mission Area, Environmental Health Infrastructure Project, Minnesota Tapwater Exposure Study, 2019. See Table S2 for additional analytical details. For data on all organic compounds detected please see Romanok et al., 2023 (https://doi.org/10.5066/P9YQ24QW)

[MCL shown in red, Maximum Contaminant Level set by the U.S. Environmental Protection Agency; MCLG, Maximum Contaminant Level Goal, maximum level of a contaminant in drinking water at which no known or anticipated adverse effect on the health of persons would occur, allowing an adequate margin of safety. MCLG shown in orange are non-enforceable public health goals. MCL and MCLG are available at https://www.epa.gov/dwregdev/how-epa-regulates-drinking-water-contaminants. WHO, World Health Organization drinking water guidelines. HA exceedances, number of exceedances of health advisories, including MCLG.]

Site code	Site type	Source of water	Chloride (mg/L)	Fluoride (mg/L)	Nitrate-N (NO3-N; mg/L)	Phosphorus-P (mg/L)	Magnesium (mg/L)	Potassium (mg/L)	Aluminum (µg/L)	Arsenic (µg/L)	Barium (µg/L)	Boron (µg/L)	Cadmium (µg/L)	Chromium (µg/L)	Chromium (Hexavalent; µg/L)	Cobalt (µg/L)	Copper (µg/L)	Iron (µg/L)	Lead (µg/L)	Lithium (µg/L)	Manganese (µg/L)	Nickel (µg/L)	Selenium (µg/L)	Strontium (µg/L)	Uranium (µg/L)	Zinc (µg/L)	SHA exceedances (MCL)	SHA exceedances (MCLG)
MNTW2019 023	Source	SW	181	0.1	1.07	nd	50.7	3.27	9	nd	140	31	nd	nd	nd	nd	6	20.6	nd	9.9	5	nd	nd	182	nd	12	0	0
MNTW2019 022	Finished	SW	78	0.6	0.37	0.7	34.9	3.95	9	nd	189	70	nd	nd	nd	nd	2	4	nd	11	17	nd	nd	303	4	7	0	1
MNTW2019 025	Tap	SW	60	0.58	0.24	0.8	32.7	3.94	9	nd	186	73	nd	nd	nd	nd	195	7	0.6	10	12	nd	nd	305	nd	29	0	1
MNTW2019 024	Tap	SW	71	0.59	0.32	0.8	33.3	3.9	9	nd	190	73	nd	nd	nd	nd	504	14.9	0.8	11	8	nd	nd	305	nd	17	0	1
MNTW2019 010	Source	GW	81	0.16	nd	nd	38.8	1.96	8	nd	228	27	nd	0.8	0.6	nd	nd	761	nd	5.5	59	nd	nd	207	nd	12	0	0
MNTW2019 009	Finished	GW	84	0.23	0.03	0.3	38.7	1.99	7	nd	227	28	nd	nd	nd	nd	20	1280	0.8	5.4	67	nd	nd	209	5	48	0	2
MNTW2019 007	Tap	GW	46	0.62	0.04	0.7	34.5	1.91	8	nd	152	31	nd	0.3	0.3	nd	82	16.7	nd	5.3	16	nd	nd	178	nd	13	0	0
MNTW2019 008	Tap	GW	83	0.6	0.04	0.7	38.5	2.02	8	nd	198	24	nd	nd	nd	nd	327	116	0.6	5.7	58	nd	nd	201	nd	11	0	1
MNTW2019 027	Source	GW	73	0.08	8.03	nd	37.7	1.2	8	nd	53	10	nd	2.4	0.7	nd	32	nd	nd	2.5	nd	nd	nd	109	nd	110	0	0
MNTW2019 026	Finished	GW	114	0.13	NR	nd	37.4	1.19	6	nd	47	10	nd	nd	nd	nd	nd	nd	nd	2.4	nd	nd	nd	103	nd	5	0	0
MNTW2019 030	Tap	GW	72	0.08	5.28	nd	30.7	1.48	7	nd	46	16	nd	0.2	0.2	nd	80	nd	nd	3.1	nd	nd	nd	100	nd	35	0	0
MNTW2019 028	Source	GW	38	0.07	5.93	nd	27.4	1.76	7	nd	48	21	nd	0.4	0.4	nd	6	32.5	nd	3.6	1	nd	nd	97	nd	28	0	0
MNTW2019 029	Finished	GW	38	0.1	5.84	nd	27.2	1.77	7	nd	49	23	nd	0.4	0.4	nd	47	20.7	2.7	3.6	nd	nd	nd	97	nd	21	0	1
MNTW2019 031	Tap	GW	37	0.08	5.19	nd	27.1	1.76	9	nd	48	22	nd	nd	nd	nd	94	149	2.9	3.8	8	nd	nd	97	nd	356	0	1
MNTW2019 037	Source	GW	38	0.16	nd	nd	49.8	2.75	8	nd	111	32	nd	nd	nd	nd	7	12.5	1.3	7.2	598	nd	nd	224	nd	11	0	1
MNTW2019 036	Finished	GW	85	0.27	0.13	0.1	45.3	2.99	9	nd	138	41	nd	nd	nd	nd	9	nd	0.6	7.9	3	15	nd	208	nd	128	0	1
MNTW2019 038	Tap	GW	87	0.65	0.08	0.1	43.6	3.1	8	nd	140	41	nd	nd	nd	nd	12	6	nd	8.3	27	nd	nd	212	nd	138	0	0
MNTW2019 039	Tap	GW	90	0.6	0.07	0.2	44.1	3.18	8	nd	136	40	nd	nd	nd	nd	1040	45.8	nd	8.7	8	nd	nd	213	5	124	0	1
MNTW2019 011	Source	SW	27	0.08	nd	0.1	14.8	2	183	nd	58	25	nd	0.8	0.4	nd	5	985	nd	4.2	382	nd	nd	92	4	9	0	1
MNTW2019 012	Finished	SW	30	0.8	0.38	0.3	2.06	2.07	23	nd	14	19	nd	0.5	0.2	nd	2	2	nd	4.2	nd	nd	nd	70	nd	4	0	0
MNTW2019 013	Tap	SW	28	0.81	0.60	0.3	1.94	2.05	34	nd	13	18	nd	0.5	0.5	nd	45	17	1.3	4.5	nd	nd	nd	77	nd	8	0	1
MNTW2019 014	Tap	SW	27	0.79	0.62	0.3	2.13	2.03	29	nd	13	19	nd	0.3	nd	nd	43	24.5	0.7	4.5	nd	nd	nd	77	nd	9	0	1
MNTW2019 020	Source	GW	43	0.13	5.51	nd	37	3.34	8	nd	225	37	nd	0.6	0.4	nd	36	nd	4.5	13	26	nd	nd	278	nd	15	0	1
MNTW2019 019	Finished	GW	48	0.14	5.61	nd	37	3.3	10	nd	242	35	nd	0.4	0.3	nd	47	nd	8.4	13	354	nd	nd	277	nd	26	0	1
MNTW2019 006	Tap	GW	54	0.66	4.31	nd	38.8	3.32	8	nd	126	37	nd	0.4	0.3	nd	187	nd	1.6	13	1	nd	nd	280	nd	138	0	1
MNTW2019 018	Source	GW	81	0.1	4.65	nd	32.9	3.46	7	nd	213	26	nd	0.5	nd	nd	5	nd	nd	8.9	35	6	nd	182	nd	20	0	0
MNTW2019 017	Finished	GW	80	0.65	4.74	nd	32.3	3.44	9	nd	215	26	nd	0.4	nd	nd	128	6	78.1	8.8	35	nd	nd	179	4	27	1	2
MNTW2019 021	Tap	GW	49	0.6	3.89	nd	35	3.31	9	nd	80	43	nd	0.5	0.6	nd	24	2	nd	9.8	nd	nd	nd	231	nd	23	0	0
MNTW2019 002	Source	GW	90	1.14	3.45	nd	36.6	2.11	8	nd	69	24	nd	1.8	0.7	nd	8	nd	nd	5.7	5	nd	nd	148	nd	37	0	0
MNTW2019 005	Tap	GW	8.9	0.26	nd	nd	0.05	0.48	nd	nd	nd	29	nd	nd	nd	nd	22	12.1	nd	1.3	nd	nd	nd	nd	nd	7	0	0
MNTW2019 004	Source	SW	33	0.11	nd	nd	12.4	2.14	6	nd	41	19	nd	nd	nd	nd	194	51.3	nd	3	46	nd	nd	70	nd	5	0	0
MNTW2019 003	Finished	SW	45	0.68	0.17	nd	1.7	2.27	16	nd	13	19	nd	nd	nd	nd	5	30.5	11.6	3.1	nd	7	nd	69	nd	13	0	1
MNTW2019 001	Tap	SW	41	0.66	0.21	nd	1.63	2.27	18	nd	13	17	nd	nd	nd	nd	5	11.1	3	3.2	nd	nd	nd	68	nd	15	0	1
MNTW2019 016	Source	GW	76	0.08	0.03	nd	37.7	2.32	9	2	255	28	nd	nd	nd	nd	1	1840	0.6	8	608	nd	nd	186	4	12	0	3
MNTW2019 015	Tap	GW	76	0.07	nd	nd	37.5	2.33	8	nd	260	25	nd	nd	nd	nd	3	715	nd	8	577	nd	nd	190	4	641	0	1
MNTW2019 033	Source	GW	39	0.2	0.79	0.4	23.8	2.4	7	nd	64	9	nd	nd	nd	nd	18	3	0.9	2.3	43	4	3	72	4	20	0	2
MNTW2019 032	Finished	GW	39	5.52	0.79	0.1	23.9	2.37	9	nd	69	10	3	nd	nd	4	48	403	23.2	2.5	211	16	3	72	nd	1110	2	2
MNTW2019 034	Tap	GW	41	0.79	0.77	0.2	0.1	0.4	nd	nd	nd	9	nd	nd	nd	nd	459	5	0.9	nd	5	5	3	nd	nd	29	0	1
MNTW2019 035	Tap	GW	44	0.79	0.58	0.1	26.8	1.56	7	nd	25	8	nd	nd	nd	nd	404	21.3	2.3	3.3	9	7	nd	79	6	63	0	2

Table S5. Quality assurance organic and inorganic analyte results (units listed) laboratory blanks analyzed at the U.S. Geological Survey, Redox Chemistry laboratory, Boulder, Colorado in bottled water samples collected for the USGS Ecosystems Mission Area, Environmental Health Infrastructure Project, Minnesota Tapwater Exposure Study, 2019. See Table S2 for analytical details and detection limits.

[mg/L; milligram per liter; nd, not detected; µg/L, microgram per liter]
[Inorganic and organic analyte results were censored to the corresponding maximum concentration detected in laboratory blanks, above.]
[No inorganic analyte results were censored because detections were higher than twice the concentration detected in laboratory blanks. Organic analyte results for 2-i-Pr-6-Me-4-pyrimidinol were censored to the concentration of the blank and it resulted in removal from the dataset.]

**Blank number**	**Blank Type**	**Potassium (mg/L)**	**Sodium (mg/L)**	**2-i-Pr-6-Me-4-pyrimidinol (µg/L)**
1	Field	nd	nd	nd
2	Field	nd	nd	0.0053
3	Field	nd	nd	0.0039
4	Field	nd	nd	0.0040
5	Laboratory	0.006	nd	--
6	Laboratory	nd	0.054	--

Table S6. Quality assurance surrogate recovery or internal dilution standard summary statistics (percent) for the U.S. Geological Survey (USGS) Ecosystems Mission Area, Environmental Health Infrastructure Project, Minnesota Tapwater Exposure Study, 2019.

[NWQL; USGS National Water Quality Laboratory, OCRL; USGS Organic Chemistry Research Laboratory]

**Analyzing Agency**	**Surrogate compound name**	**Minimum (%)**	**Median (%)**	**Maximum (%)**
OCRL	Bromofluorobenzene	101	104	107
OCRL	1,2-Dichlorobenzene-d4	95.6	101	104
NWQL	62835 Isobutylalcohol-d6	87.5	113	122
NWQL	90359 Lorazepam-d4	73.3	100	123
NWQL	90360 Propoxyphene-d11	116	150	196
NWQL	90361 Ketoconazole-d4	8.3	40.1	178
NWQL	90362 Oxazepam-d5	75.1	93.5	121
NWQL	90363 Carisoprodol-d7	83.4	96.4	107
NWQL	90364 Amitriptyline-d3rog	111	175	241

Analyzing Agency	Surrogate compound name	Minimum (%)	Median (%)	Maximum (%)
NWQL	90365 Erythromycin-13C,d3	55	139	216
NWQL	90366 Ezetimibe-d4	50.7	110	226
NWQL	90367 Fenofibrate-d6	17.4	51.3	101
NWQL	90368 Fexofenadine-d10	85.4	109	122
NWQL	90369 Fluvoxamine-d4	51	125	178
NWQL	90370 Loperamide-d6	46.7	126	218
NWQL	90371 Loratadine-d4	32.2	61.7	90.4
NWQL	90372 Desmethyldiltiazem-d4	91.3	141	190
NWQL	90373 Promethazine-d6	0	139	874
NWQL	90374 Raloxifene-d10	43.6	120	404
NWQL	90375 Ranitidine-d6	0.6	147	747
NWQL	90376 Tiotropium-d3	111	200	324
NWQL	90377 Verapamil-d6	49.7	162	290
NWQL	90378 Tamoxifen-d5	31.9	98.6	413
NWQL	90379 Hydrocortisone-13C3	66.4	105	147
NWQL	90395 Metformin-d6	14.8	72	83.8
NWQL	90516 3-Phenoxybenzoic acid-13C6	99.4	109	115
NWQL	90517 Acetochlor-d11	89.4	109	132
NWQL	90518 Alachlor-d13	92.3	105	114
NWQL	90519 Carbaryl-d7	97.6	110	123
NWQL	90520 Carbendazim-d4	79.6	101	116
NWQL	90521 Carbofuran-d3	103	111	123
NWQL	90522 Deethylatrazine-d6	76.1	95.3	110
NWQL	90523 Diazinon-d10	91	109	145
NWQL	90524 Diflubenzuron-d4	91.7	106	129
NWQL	90527 Hexazinone-d6	93.2	103	116
NWQL	90529 Linuron-d6	97.4	103	119
NWQL	90552 Malathion-d10	94	114	129
NWQL	90553 Metolachlor-d6	92.3	101	114
NWQL	90554 Nicosulfuron-d6	7.78	101	131
NWQL	90555 Tebuconazole-d6	58.3	95.2	113
NWQL	90556 Thiobencarb-d10	91.6	105	114
NWQL	90558 cis-Permethrin-13C6/is	42.4	76	120
NWQL	90576 1-Bromo-3-chloropropane-d6	72.1	82.8	89.2
NWQL	90607 Tetrahydrofuran-d8	43.5	77.6	113
NWQL	90624 Butachlor SA	92	109	117
NWQL	90625 Dimethachlor SA	96.6	112	123
NWQL	90695 1,4-BrFbenzeneamb P&T	81.7	86	90.2
NWQL	90696 Toluene-d8amb P&T	91.2	93.1	95.9
NWQL	90701 1,4-BrFbenzeneheat P&T	93.5	101	109

Analyzing Agency	Surrogate compound name	Minimum (%)	Median (%)	Maximum (%)
NWQL	90703 Toluene-d8heat P&T	93.2	96.8	102
NWQL	90808 Diuron-d6	91.3	97.4	117
NWQL	91769 Thiabendazole-d4	61.1	84.8	92.1
NWQL	91772 Albuterol-d9	103	124	144
NWQL	91773 Diltiazem-d3	96.9	150	201
NWQL	91774 Trimethoprim-d9	77.1	91.6	100
NWQL	91775 Acetaminophen-d3	63.7	111	249
NWQL	91776 Norfluoxetine-d6	65	92.5	105
NWQL	91777 Methadone-d9	113	177	223
NWQL	91778 Oxycodone-d3	95.7	119	137
NWQL	91779 Hydrocodone-d3	103	111	121
NWQL	91780 Temazepam-d5	83.1	94	104
NWQL	91781 Caffeine-(trimethyl-13C3)	82.3	98.8	118
NWQL	91782 Sulfamethoxazole-(phenyl13C6)	88.2	101	172
NWQL	91783 Cotinine-d3rog	79.1	89.7	96.7
NWQL	91784 Amphetamine-d6	80.4	104	112
NWQL	91786 Codeine-d6	81.1	112	134
NWQL	91787 Pseudoephedrine-d3	80.1	101	109
NWQL	91788 Diphenhydramine-d3	115	150	178
NWQL	91789 Fluoxetine-d6	67.7	133	182
NWQL	91790 Diazepam-d5	84.7	93	99.8
NWQL	91986 2,4-D-d3	83.3	93.2	101
NWQL	99832 1,2-Dichloroethane-d4	97.8	104	112

Table S7. Mammalian Bioactivities in ng equivalents per liter (ng L-1) of standard steroid hormone (E2, DHT, DEX) analyzed in source water, finished water, and service-area tapwater samples collected in Minnesota, 2019. Quantifiable endocrine activities above and below MDC are shown in Bold and Italics fonts, respectively. Blue Font and "FB" Site ID suffix indicate a Field Blank. Quantifiable endocrine activity below MDC is italicized.

[DEX, dexamethasone; DHT, 4,5a-dihydrotestosterone; E2, 17b-estradiol; EC50, half maximum effect concentration in ng/L; MDC, Minimum Detectable Concentration in ng/L; NA, not applicable; ND, no endocrine activity detected compared to vehicle control treated cells.]

Site Code	Site type	Source of water	E2	DHT	DEX
MNTW2019 023	Source	Surface water	0.868	*0.0665*	ND
MNTW2019 022	Finished	Surface water	ND	ND	ND
MNTW2019 024	Tap	Surface water	ND	ND	ND
MNTW2019 025	Tap	Surface water	ND	ND	ND
MNTW2019 010	Source	Groundwater	ND	ND	ND
MNTW2019 009	Finished	Groundwater	ND	ND	ND
MNTW2019 007	Tap	Groundwater	ND	ND	ND
MNTW2019 008	Tap	Groundwater	ND	ND	ND
MNTW2019 027	Source	Groundwater	ND	ND	ND
MNTW2019 026	Finished	Groundwater	ND	ND	ND
MNTW2019 030	Tap	Groundwater	ND	ND	ND
MNTW2019 028	Source	Groundwater	ND	ND	ND
MNTW2019 029	Finished	Groundwater	ND	ND	ND
MNTW2019 031	Tap	Groundwater	ND	ND	ND
MNTW2019 037	Source	Groundwater	ND	ND	ND
MNTW2019 036	Finished	Groundwater	ND	ND	ND
MNTW2019 038	Tap	Groundwater	ND	ND	ND
MNTW2019 039	Tap	Groundwater	ND	ND	ND
**MNTW2019 039 FB**	**Blank**	**NA**	**ND**	**ND**	**ND**
MNTW2019 011	Source	Surface water	0.112	ND	ND
MNTW2019 012	Finished	Surface water	0.210	ND	ND
MNTW2019 014	Tap	Surface water	ND	ND	ND
MNTW2019 013	Tap	Surface water	ND	ND	ND
MNTW2019 020	Source	Groundwater	ND	ND	ND
MNTW2019 019	Finished	Groundwater	ND	ND	ND
MNTW2019 006	Tap	Groundwater	ND	ND	ND
MNTW2019 018	Source	Groundwater	ND	ND	ND
MNTW2019 017	Finished	Groundwater	ND	ND	ND
MNTW2019 021	Tap	Groundwater	ND	ND	ND
MNTW2019 002	Source	Groundwater	ND	ND	ND
MNTW2019 005	Tap	Groundwater	ND	ND	ND
**MNTW2019 005 FB**	**Blank**	**NA**	**ND**	**ND**	**ND**
MNTW2019 004	Source	Surface water	0.032	ND	ND
MNTW2019 003	Finished	Surface water	ND	ND	ND
MNTW2019 001	Tap	Surface water	ND	ND	ND

Site Code	Site type	Source of water	E2	DHT	DEX
MNTW2019 016	Source	Groundwater	ND	ND	ND
**MNTW2019 016**	**Blank**	**NA**	**ND**	**ND**	**ND**
MNTW2019 015	Tap	Groundwater	*0.028*	ND	ND
MNTW2019 033	Source	Groundwater	*0.028*	ND	ND
MNTW2019 032	Finished	Groundwater	ND	ND	ND
MNTW2019 034	Tap	Groundwater	ND	ND	ND
MNTW2019 035	Tap	Groundwater	ND	ND	ND
**MNTW2019 035 FB**	**Blank**	**NA**	**ND**	**ND**	**ND**
**MDC**			0.0280	0.7400	6.5900
**Standard EC_{50}**			0.4620	4.9500	72.1000
**# greater than MDC**			6	0	0

Table S8. Compound: endpoint combinations excluded from ToxCast evaluation due to unreliable concentration-response relationship and resulting lack of confidence in activity concentration at cutoff (ACC).

[CAS number, Chemical Abstract Services Registry Number; endpoint, ToxCast assay endpoint name (https://www.epa.gov/chemical-research/toxicity-forecasting)]

CAS number	endPoint
84852-15-3	NVS_ENZ_hPTEN
84-66-2	NVS_ENZ_rMAOBP
486-56-6	TOX21_p53_BLA_p3_viability
51218-45-2	TOX21_p53_BLA_p5_ratio
86−74−8	TOX21_p53_BLA_p3_ratio
115−86−6	TOX21_p53_BLA_p3_ratio
115−96−8	TOX21_p53_BLA_p3_ratio
206−44−0	TOX21_p53_BLA_p3_ratio
115-86-6	NVS_NR_hPPARg
80-05-7	CLD_CYP1A1_6hr
80-05-7	CLD_CYP1A2_6hr
82657-04-3	BSK_3C_Vis_down
82657-04-3	TOX21_NFkB_BLA_agonist_ch1
82657-04-3	NVS_NR_hPR
120068-37-3	NVS_NR_hCAR_Antagonist
60-51-5	NVS_NR_bER
13194-48-4	NCCT_QuantiLum_inhib_dn
161050-58-4	ATG_Myb_CIS_up
173584-44-6	ATG_HSE_CIS_up
30560-19-1	BSK_3C_SRB_up
30560-19-1	ATG_M_19_TRANS_dn

CAS number	endPoint
61949-77-7	ATG_CMV_CIS_dn
94-74-6	NVS_NR_hCAR_Antagonist
94-74-6	NVS_NR_hPPARg
51218-45-2	TOX21_p53_BLA_p5_ch2
759-94-4	ATG_Xbp1_CIS_dn
759-94-4	ATG_MRE_CIS_dn
759-94-4	ATG_HNF6_CIS_dn
759-94-4	ATG_GATA_CIS_dn
759-94-4	ATG_Myb_CIS_dn
759-94-4	ATG_PBREM_CIS_dn
759-94-4	TOX21_p53_BLA_p3_viability
834-12-8	TOX21_p53_BLA_p4_ratio
122836-35-5	ATG_TA_CIS_dn
87674-68-8	TOX21_p53_BLA_p2_ch2
87674-68-8	TOX21_p53_BLA_p2_ratio
111991-09-4	TOX21_AutoFluor_HEK293_Media_blue
51235-04-2	TOX21_p53_BLA_p5_ratio
51235-04-2	TOX21_p53_BLA_p5_ch1
51235-04-2	TOX21_p53_BLA_p5_viability
51218-45-2	TOX21_AR_LUC_MDAKB2_Agonist
51218-45-2	TOX21_AR_LUC_MDAKB2_Agonist_Counterscreen
51218-45-2	TOX21_p53_BLA_p5_ratio
75-05-8	TOX21_TSHR_Agonist_ratio
75-05-8	TOX21_TSHR_wt_ratio
75-05-8	TOX21_TSHR_HTRF_Agonist_ch1
1912-24-9	BSK_KF3CT_IP10_up
75-25-2	TOX21_AR_LUC_MDAKB2_Agonist_Counterscreen
122-34-9	APR_HepG2_CellCycleArrest_1h_up
124-48-1	TOX21_RT_HEK293_FLO_32hr_viability

Table S9. Site-specific Exposure Activity Ratios (EAR) for those compounds with exact Chemical Abstract Service (CAS) number matches and with reliable concentration-response relationship and ACC data in ToxCast, Minnesota Tapwater Exposure Study 2019.

[blank cells indicate chemical not detected or that EAR (or SEAR) is <0.00001). Of 88 detected organics, 44 had reliable ACC data in ToxCast and EAR ≥0.00001. Red and orange fonts indicate exceedance of presumptive effects threshold (1) and precautionary screening level of concern (0.001), respectively.]

Site Name	Site Type	Source of Water	(E)-1,2-Dichloroethylene	1,1-Dichloroethane	2,4-Dichlorophenoxyacetic acid	2-Ethyl-1-hexanol	Atrazine	Azoxystrobin	Bentazone	Bromacil	Bromochloromethane	Butanal	Caffeine	Carbamazepine	Carbon tetrachloride	Chloroacetonitrile
MNTW2019 023	Source	SW	-	-	-	-	-	0.00026	-	0.00007	-	-	-	-	-	-
MNTW2019 022	Finished	SW	-	-	-	-	-	-	-	-	-	-	-	-	-	0.00011
MNTW2019 025	Tap	SW	-	-	-	-	-	-	-	-	-	-	-	-	-	-
MNTW2019 024	Tap	SW	-	0.00343	-	-	-	-	-	-	-	-	-	-	-	0.00034
MNTW2019 010	Source	GW	0.00007	-	-	-	-	-	-	-	-	-	-	-	-	-
MNTW2019 009	Finished	GW	0.00006	-	-	-	-	-	-	-	-	-	-	-	-	-
MNTW2019 007	Tap	GW	0.00007	0.02212	-	-	-	-	-	0.00043	-	-	-	-	-	0.00023
MNTW2019 008	Tap	GW	0.00004	-	-	-	-	-	-	-	-	-	-	-	-	0.00011
MNTW2019 027	Source	GW	-	-	-	-	0.00088	-	-	-	-	-	-	-	-	-
MNTW2019 026	Finished	GW	-	-	-	-	0.00086	-	-	-	-	-	-	-	-	-
MNTW2019 030	Tap	GW	-	-	-	-	0.00084	-	-	-	-	-	0.00112	-	-	-
MNTW2019 028	Source	GW	-	-	-	-	0.00097	-	0.00001	-	-	-	-	0.00085	-	-
MNTW2019 029	Finished	GW	-	-	-	-	0.00088	-	0.00001	-	-	0.00108	-	0.00075	-	-
MNTW2019 031	Tap	GW	-	-	-	-	0.00093	-	0.00001	-	-	-	-	0.00070	-	-
MNTW2019 037	Source	GW	-	-	-	-	-	-	-	-	-	-	-	-	-	-
MNTW2019 036	Finished	GW	-	-	-	-	0.00008	-	-	-	-	-	-	-	-	-
MNTW2019 038	Tap	GW	-	-	-	-	-	-	-	-	-	-	-	-	-	0.00011
MNTW2019 039	Tap	GW	-	0.00343	-	-	-	-	-	-	0.00407	-	-	-	-	-
MNTW2019 011	Source	SW	-	-	0.00629	0.01867	0.00059	0.00400	0.00001	0.00042	-	0.01579	0.00152	0.00109	-	-
MNTW2019 012	Finished	SW	-	-	-	-	-	-	-	-	-	-	-	-	-	-
MNTW2019 013	Tap	SW	-	-	-	-	-	-	-	-	-	-	-	-	-	-
MNTW2019 014	Tap	SW	-	-	-	-	-	-	-	-	-	-	-	-	-	-
MNTW2019 020	Source	GW	-	-	-	-	0.00043	-	-	-	-	-	-	-	-	-
MNTW2019 019	Finished	GW	-	-	-	-	0.00045	-	-	-	-	-	-	-	-	0.00011
MNTW2019 006	Tap	GW	-	-	-	-	0.00038	-	-	-	-	-	-	-	-	-
MNTW2019 018	Source	GW	-	-	-	-	0.00055	-	-	0.00257	-	-	-	-	-	-
MNTW2019 017	Finished	GW	-	-	-	-	0.00051	-	-	0.00037	-	-	-	-	-	-
MNTW2019 021	Tap	GW	-	-	-	-	0.00055	-	-	-	-	-	-	-	-	0.00011
MNTW2019 002	Source	GW	-	-	-	-	0.00015	-	-	-	-	-	-	-	-	-
MNTW2019 005	Tap	GW	-	-	-	-	-	-	-	-	-	-	-	-	-	-
MNTW2019 004	Source	SW	-	-	0.00841	-	0.00100	0.00097	-	-	-	0.00324	-	-	-	-
MNTW2019 003	Finished	SW	-	-	-	-	0.00096	-	-	-	-	-	-	-	0.00006	0.00034
MNTW2019 001	Tap	SW	-	-	0.00474	-	0.00089	-	-	-	-	-	-	-	0.00006	0.00023
MNTW2019 016	Source	GW	-	-	-	-	-	-	-	0.00024	-	-	-	-	-	-
MNTW2019 015	Tap	GW	-	-	-	-	-	-	-	-	-	-	-	-	-	-
MNTW2019 033	Source	GW	-	-	-	-	0.00039	-	-	-	-	-	-	-	-	-
MNTW2019 032	Finished	GW	-	-	-	-	0.00043	-	-	-	-	-	-	-	-	-
MNTW2019 034	Tap	GW	-	-	-	-	0.00035	-	-	-	-	-	-	-	-	0.00045
MNTW2019 035	Tap	GW	-	-	-	-	0.00027	-	-	-	-	-	-	-	-	0.00011

Site Name	Cotinine	Cyclohexanone	Deethylatrazine	Deisopropylatrazine	Dibromochloromethane	Dichloromethane	Dimethenamid	Ethyl acetate	Isopropyl alcohol	Lidocaine	Metalaxyl	Methocarbamol	Methyl-1H-benzotriazole	Metolachlor	Metolachlor ESA
MNTW2019 023	-	-	-	-	-	-	-	-	-	-	-	-	-	-	-
MNTW2019 022	-	-	-	-	0.98287	-	-	-	-	-	-	-	-	-	-
MNTW2019 025	-	-	-	-	1.02382	-	-	-	0.00090	-	-	-	-	-	-
MNTW2019 024	-	-	-	-	1.63811	-	-	0.00007	0.00271	-	-	-	-	-	-
MNTW2019 010	-	-	-	-	-	-	-	-	-	-	-	-	-	-	-
MNTW2019 009	-	-	-	-	0.08191	-	-	-	-	-	-	-	-	-	-
MNTW2019 007	-	-	-	-	0.04095	-	-	-	-	-	-	-	-	-	-
MNTW2019 008	-	-	-	-	0.14333	-	-	-	0.00090	-	-	-	-	-	-
MNTW2019 027	-	-	0.00003	0.00028	-	-	-	-	-	-	-	-	-	-	0.00006
MNTW2019 026	-	-	0.00003	-	0.20476	-	-	-	-	-	0.00014	-	-	-	0.00010
MNTW2019 030	-	-	0.00004	0.00038	0.28667	-	-	0.00004	-	-	-	-	-	-	0.00014
MNTW2019 028	-	-	0.00004	0.00031	-	-	-	0.00004	-	-	0.00011	-	-	-	0.00021
MNTW2019 029	-	-	0.00004	0.00048	-	-	-	-	-	-	-	-	-	-	0.00021
MNTW2019 031	-	-	0.00004	0.00038	0.02048	-	-	-	-	-	-	-	-	-	0.00020
MNTW2019 037	-	0.00024	-	-	-	-	-	-	0.00135	-	-	-	-	-	-
MNTW2019 036	-	-	-	-	0.90096	-	-	0.00007	-	-	-	-	-	-	-
MNTW2019 038	-	-	-	-	1.14668	-	-	-	0.00090	-	-	-	-	-	-
MNTW2019 039	-	-	-	-	1.14668	0.01392	-	-	0.00060	-	-	-	-	-	-
MNTW2019 011	0.00012	0.00184	0.00001	0.00031	-	-	0.00004	-	0.03326	0.00191	-	0.00058	0.0003	0.00142	0.00010
MNTW2019 012	-	-	-	-	0.08191	-	-	-	-	-	-	-	-	-	0.00004
MNTW2019 013	-	-	-	-	0.08191	-	-	-	-	-	-	-	-	-	-
MNTW2019 014	-	-	-	-	0.06143	-	-	-	-	-	-	-	-	-	-
MNTW2019 020	-	-	0.00002	0.00114	-	-	-	0.00004	-	-	-	-	-	-	0.00007
MNTW2019 019	-	-	0.00002	0.00106	0.45048	-	-	-	-	-	-	-	-	-	0.00006
MNTW2019 006	-	-	0.00002	0.00061	0.57334	-	-	0.00005	-	-	-	-	-	-	0.00003
MNTW2019 018	-	-	0.00002	0.00094	-	-	-	-	-	-	-	-	-	-	0.00002
MNTW2019 017	-	-	0.00002	0.00070	0.51191	-	-	-	-	-	-	-	-	-	0.00003
MNTW2019 021	-	-	0.00002	0.00057	0.71667	-	-	-	-	-	-	-	-	-	0.00003
MNTW2019 002	-	-	-	-	-	-	-	-	-	-	0.00007	-	-	-	-
MNTW2019 005	-	-	-	-	-	-	-	-	-	-	-	-	-	-	-
MNTW2019 004	-	-	0.00002	-	-	-	0.00007	-	0.00120	-	-	-	-	0.00151	0.00003
MNTW2019 003	-	-	0.00002	-	0.08191	-	-	-	-	-	-	-	-	0.00093	-
MNTW2019 001	-	-	0.00002	0.00032	0.06143	-	-	-	-	-	-	-	-	0.00101	0.00003
MNTW2019 016	-	-	-	-	-	-	-	-	-	-	-	-	-	-	-
MNTW2019 015	-	-	-	-	-	-	-	-	-	-	-	-	-	-	-
MNTW2019 033	-	-	0.00002	0.00033	-	-	-	-	-	-	-	-	-	-	0.00007
MNTW2019 032	-	-	0.00003	-	-	-	-	-	0.00090	-	-	-	-	-	0.00007
MNTW2019 034	-	-	0.00002	0.00022	0.20476	-	-	0.00005	0.00090	-	-	-	-	-	0.00005
MNTW2019 035	-	-	0.00001	-	0.49143	-	-	-	-	-	-	-	-	-	0.00004

Site Name	Nicotine	o-Xylene	Pentanal	Pentane	Perfluorobutanoic acid (PFBA)	Perfluorononanoic acid (PFNA)	Perfluorooctanoic acid (PFOA)	Piperonyl butoxide	Prometon	Propazine	Simazine	Terbutylazine	Tribromomethane (bromoform)	Trichloroethylene	Trichloromethane	∑EAR
MNTW2019 023	-	-	0.00007	-	-	-	-	0.00055	0.00546	-	-	-	-	-	0.00082	0.00722
MNTW2019 022	-	-	-	-	-	-	-	-	-	-	-	-	0.00194	-	0.00145	0.98636
MNTW2019 025	-	-	0.00004	-	-	-	-	-	-	-	-	-	0.00183	-	0.00176	1.02835
MNTW2019 024	-	0.00821	0.00016	-	-	-	-	-	-	-	-	-	0.00258	-	0.00604	1.66164
MNTW2019 010	-	-	-	-	-	-	0.00007	-	-	-	-	-	-	0.05854	-	0.05868
MNTW2019 009	-	-	-	-	-	-	-	-	-	-	-	-	0.00011	0.04154	0.00050	0.12412
MNTW2019 007	-	-	-	-	-	-	-	-	0.00213	-	-	-	-	0.03777	0.00082	0.10451
MNTW2019 008	0.0010	-	-	-	-	-	-	-	-	-	-	-	0.00022	0.02833	0.00057	0.17453
MNTW2019 027	-	-	-	-	0.00004	-	0.00011	-	0.00579	-	0.00013	-	-	-	0.00006	0.00738
MNTW2019 026	-	-	-	-	0.00003	-	0.00010	-	0.00662	-	-	0.00037	0.00075	-	0.00013	0.21389
MNTW2019 030	-	-	0.00004	-	0.00002	-	0.00008	-	0.00407	-	-	-	0.00151	0.07176	0.00013	0.36682
MNTW2019 028	-	-	-	-	0.00001	-	0.00009	-	0.00509	0.00007	-	-	-	0.15673	-	0.16454
MNTW2019 029	-	-	0.00003	-	0.00002	-	-	-	0.00404	0.00006	-	-	-	0.12652	-	0.13413
MNTW2019 031	-	-	-	-	0.00002	-	0.00008	-	0.00460	-	-	-	0.00011	0.13785	0.00006	0.16544
MNTW2019 037	-	-	-	-	-	-	-	-	-	-	-	-	-	-	-	0.00159
MNTW2019 036	-	-	-	-	-	-	-	-	-	-	-	-	0.00258	0.42299	0.00069	1.32737
MNTW2019 038	-	-	0.00005	-	-	-	-	-	-	-	-	-	0.00355	0.46454	0.00189	1.61772
MNTW2019 039	-	-	-	-	-	-	-	-	-	-	-	-	0.00258	0.07176	0.00277	1.24580
MNTW2019 011	0.0013	-	0.00043	-	-	-	-	-	-	-	-	-	-	0.04343	-	0.13338
MNTW2019 012	-	-	-	-	-	-	-	-	-	-	-	-	-	-	0.01309	0.09503
MNTW2019 013	-	0.00966	-	-	-	-	-	-	-	-	-	-	-	-	0.01076	0.10232
MNTW2019 014	-	-	-	0.00002	-	-	-	-	-	-	-	-	-	-	0.01051	0.07196
MNTW2019 020	-	-	-	-	-	-	-	-	-	-	-	-	-	-	0.00006	0.00176
MNTW2019 019	-	-	-	-	-	-	-	-	-	-	-	-	0.00065	-	0.00145	0.45427
MNTW2019 006	0.0010	-	0.00005	-	-	-	-	-	-	-	-	-	0.00097	-	0.00113	0.57758
MNTW2019 018	-	-	-	-	-	-	-	-	-	-	-	-	-	-	-	0.00410
MNTW2019 017	-	-	0.00002	-	-	-	0.00003	-	-	-	-	-	0.00097	-	0.00082	0.51537
MNTW2019 021	-	0.01690	0.00011	-	-	-	-	-	0.00202	-	-	-	0.00118	-	0.00208	0.74025
MNTW2019 002	-	-	-	-	0.00001	-	0.00006	-	0.00114	-	-	-	-	-	-	0.00143
MNTW2019 005	-	-	-	-	-	-	0.00004	-	-	-	-	-	-	-	-	0.00004
MNTW2019 004	-	-	-	-	-	-	-	-	0.00103	-	-	-	-	-	-	0.01749
MNTW2019 003	-	0.00338	-	-	-	-	0.00005	-	0.00114	-	-	-	-	-	0.01806	0.10684
MNTW2019 001	-	-	-	-	-	-	0.00005	-	0.00109	0.00003	-	-	-	-	0.01813	0.08802
MNTW2019 016	-	-	-	-	0.00002	-	-	-	-	-	-	-	-	-	-	0.00026
MNTW2019 015	-	-	-	-	0.00002	-	-	-	-	-	-	-	-	-	-	0.00002
MNTW2019 033	-	-	-	-	0.00003	-	0.00009	-	-	-	-	-	-	-	-	0.00093
MNTW2019 032	-	-	-	-	0.00002	0.00013	0.00006	-	-	-	-	-	-	-	-	0.00165
MNTW2019 034	-	0.00724	0.00005	-	0.00002	-	0.00012	-	-	-	-	-	0.00032	-	0.00283	0.21739
MNTW2019 035	-	0.00579	0.00008	-	0.00002	-	-	-	-	-	-	-	0.00086	-	0.00151	0.50013

Table S10. Site-specific Exposure Activity Ratios (EAR) for all bioassay endpoints within each class shown. Data are for those compounds with exact Chemical Abstract Service (CAS) number matches and with reliable concentration-response relationship and ACC data in ToxCast, Minnesota Tapwater Exposure Study 2019.

[blank cells indicate chemical not detected or EAR (or SEAR) < 0.00001. Of 88 detected organics, 44 had reliable ACC data in ToxCast and EAR ≥0.00001. Red and orange fonts indicate exceedance of presumptive effects threshold (1) and precautionary screening level of concern (0.001), respectively.]

Site Name	Site Type	Source of Water	Cell Cycle	Nuclear Receptor	DNA Binding	Lyase	Ion Channel	GPCR	CYP	Phosphatase	Esterase	Oxidoreductase	Steroid Hormone	Zebrafish	Malformation	Cell Morphology	Transporter	Kinase	Protease	∑EAR
MNTW2019 023	Source	SW	1.65316	0.00127	0.00385	0.00336	-	-	-	-	-	-	-	-	-	-	-	-	-	1.66164
MNTW2019 022	Finished	SW	1.15540	0.08690	0.00222	0.00128	-	-	-	-	-	-	-	-	-	-	-	-	-	1.24581
MNTW2019 025	Tap	SW	1.14777	0.46624	0.00264	0.00107	-	-	-	-	-	-	-	-	-	-	-	-	-	1.61772
MNTW2019 024	Tap	SW	1.02483	0.00088	0.00163	0.00102	-	-	-	-	-	-	-	-	-	-	-	-	-	1.02835
MNTW2019 010	Source	GW	0.98341	0.00092	0.00148	0.00055	-	-	-	-	-	-	-	-	-	-	-	-	-	0.98636
MNTW2019 009	Finished	GW	0.90122	0.42422	0.00160	0.00028	-	-	-	-	-	0.00003	0.00002	-	-	-	-	-	-	1.32737
MNTW2019 007	Tap	GW	0.73628	0.00060	0.00149	0.00091	-	-	0.00056	-	-	0.00019	0.00015	0.00006	-	-	-	-	-	0.74024
MNTW2019 008	Tap	GW	0.57375	0.00048	0.00099	0.00051	-	0.00097	0.00060	-	-	0.00013	0.00011	0.00002	-	-	-	-	-	0.57757
MNTW2019 027	Source	GW	0.51220	0.00047	0.00090	0.00063	-	-	0.00070	0.00012	0.00001	0.00018	0.00014	0.00003	-	-	-	-	-	0.51537
MNTW2019 026	Finished	GW	0.49779	0.00043	0.00109	0.00063	-	-	0.00002	-	-	0.00009	0.00006	0.00001	-	-	-	-	-	0.50013
MNTW2019 030	Tap	GW	0.45103	0.00032	0.00097	0.00063	-	-	0.00100	-	-	0.00016	0.00013	0.00002	-	-	-	-	-	0.45426
MNTW2019 028	Source	GW	0.29097	0.07250	0.00127	0.00095	-	-	0.00045	0.00002	0.00002	0.00029	0.00022	0.00011	-	-	-	-	-	0.36680
MNTW2019 029	Finished	GW	0.21356	0.00017	0.00165	0.00148	-	-	0.00023	0.00002	0.00004	0.00012	0.00009	0.00002	-	-	-	-	0.00001	0.21739
MNTW2019 031	Tap	GW	0.21158	0.00036	0.00097	0.00016	-	-	0.00008	0.00003	0.00003	0.00029	0.00022	0.00015	-	-	-	-	0.00001	0.21390
MNTW2019 037	Source	GW	0.14396	0.02845	0.00053	0.00057	-	0.00101	-	-	-	-	-	-	-	-	-	-	-	0.17452
MNTW2019 036	Finished	GW	0.09532	-	0.00291	0.00409	-	-	-	-	-	-	-	-	-	-	-	-	-	0.10233
MNTW2019 038	Tap	GW	0.09293	-	0.00604	0.00703	-	-	0.00008	0.00011	0.00001	0.00032	0.00023	0.00007	-	0.00001	-	-	-	0.10684
MNTW2019 039	Tap	GW	0.08650	-	0.00356	0.00498	-	-	-	-	-	-	-	-	-	-	-	-	-	0.09503
MNTW2019 011	Source	SW	0.08208	0.04162	0.00019	0.00022	-	-	-	-	-	-	-	-	-	-	-	-	-	0.12412
MNTW2019 012	Finished	SW	0.06902	0.00002	0.00919	0.00786	0.00076	-	0.00037	0.00016	0.00002	0.00031	0.00022	0.00007	-	0.00001	0.00003	-	-	0.08803
MNTW2019 013	Tap	SW	0.06551	0.03780	0.00037	0.00065	-	-	0.00002	0.00012	-	-	-	0.00003	-	-	-	-	-	0.10451
MNTW2019 014	Tap	SW	0.06514	-	0.00283	0.00400	-	-	-	-	-	-	-	-	-	-	-	-	-	0.07197
MNTW2019 020	Source	GW	0.02498	0.13791	0.00122	0.00015	-	-	0.00043	0.00002	0.00003	0.00032	0.00024	0.00013	0.00001	-	-	-	0.00001	0.16544
MNTW2019 019	Finished	GW	0.02107	0.04362	0.03357	0.03084	0.00100	0.00126	0.00044	0.00058	0.00037	0.00021	0.00016	0.00003	0.00012	0.00006	0.00005	0.00002	-	0.13340
MNTW2019 006	Tap	GW	0.00575	0.00002	0.00044	0.00050	-	-	0.00001	0.00035	0.00002	-	0.00001	0.00009	0.00002	-	-	-	-	0.00722
MNTW2019 018	Source	GW	0.00567	-	0.00039	0.00017	-	-	0.00033	0.00009	0.00004	0.00031	0.00024	0.00014	-	-	-	-	0.00001	0.00737
MNTW2019 017	Finished	GW	0.00498	0.15674	0.00148	0.00015	-	-	0.00037	0.00003	0.00003	0.00035	0.00025	0.00014	0.00002	-	-	-	0.00001	0.16453
MNTW2019 021	Tap	GW	0.00394	0.12653	0.00121	0.00122	-	-	0.00052	0.00001	-	0.00032	0.00023	0.00011	0.00002	-	-	-	-	0.13412
MNTW2019 002	Source	GW	0.00209	0.00003	0.00746	0.00533	0.00134	-	0.00009	0.00029	0.00009	0.00033	0.00024	0.00007	0.00003	0.00004	0.00006	-	-	0.01751
MNTW2019 005	Tap	GW	0.00112	-	0.00014	0.00002	-	-	-	0.00001	0.00002	0.00005	0.00004	0.00003	-	-	-	-	-	0.00141
MNTW2019 004	Source	SW	0.00076	-	0.00033	0.00050	-	-	-	-	-	-	-	-	-	-	-	-	-	0.00159
MNTW2019 003	Finished	SW	0.00040	-	0.00043	0.00039	-	-	0.00006	0.00006	0.00002	0.00014	0.00010	0.00002	-	-	-	-	0.00001	0.00164
MNTW2019 001	Tap	SW	0.00003	0.00001	0.00022	0.00010	-	-	0.00108	-	-	0.00015	0.00012	0.00002	-	-	-	-	-	0.00175
MNTW2019 016	Source	GW	-	-	0.00021	0.00005	-	-	0.00034	0.00001	0.00003	0.00013	0.00010	0.00002	-	-	-	-	0.00001	0.00091
MNTW2019 015	Tap	GW	-	0.05858	0.00002	0.00004	-	-	-	-	0.00002	-	-	-	-	-	-	-	-	0.05866
MNTW2019 033	Source	GW	-	-	0.00001	-	-	-	-	-	0.00001	-	-	-	-	-	-	-	-	0.00003
MNTW2019 032	Finished	GW	-	-	0.00002	-	-	-	-	-	-	-	-	-	-	-	-	-	-	0.00002
MNTW2019 034	Tap	GW	-	-	0.00002	0.00016	-	-	-	0.00007	-	-	-	-	-	-	-	-	-	0.00025
MNTW2019 035	Tap	GW	-	0.00001	0.00018	0.00178	-	-	0.00099	0.00074	-	0.00021	0.00015	0.00003	-	-	-	-	-	0.00409

Table S11. Site specific toxicity quotient(s) (TQ) for human-health benchmarks (lowest concentration available from MCL, MCLG, WHO, AAP, American Academy of Pediatrics, HBSL, or HHBP benchmarks) for each compound shown. TQ is the ratio of the measured concentration to the specific benchmark selected. See Table S12 for benchmark list and links.

[blank cells indicate chemical not detected. Red font indicate exceedance of presumptive effects threshold (1)]

Site code	Site Type	Source of Water	1,1,1-Trichloroethane maxTQ	1,1-Dichloro-2-propanone maxTQ	2,4-D maxTQ	Alachlor oxanilic acid maxTQ	Atrazine maxTQ	Barium maxTQ	Bentazon maxTQ	Benzene maxTQ	Boron maxTQ	Bromacil maxTQ	Bromodichloromethane maxTQ	Cadmium maxTQ	Chromium maxTQ	cis-1,2-Dichloroethene maxTQ	Copper maxTQ	Desamino metribuzin maxTQ	Desvenlafaxine maxTQ	Dibromochloromethane maxTQ	Dichlorofluoromethane maxTQ	Dichloromethane maxTQ	Dimethenamid maxTQ	Ethylbenzene maxTQ	Fluoride maxTQ	Lead maxTQ	Manganese maxTQ	Methyl tert-butyl ether maxTQ	Methylbenzotriazole maxTQ	Metolachlor maxTQ	Metolachlor oxanilic acid maxTQ	Metolachlor sulfonic acid maxTQ
MNTW2019 023	Source	SW	-	-	-	-	-	0.108	-	-	0.062	-	-	-	-	-	0.005	-	-	-	-	-	-	-	0.025	-	0.05	-	-	-	-	-
MNTW2019 022	Finished	SW	-	-	-	-	-	0.145	-	-	0.140	-	74.0	-	-	-	0.002	-	-	0.080	-	-	-	-	0.150	-	0.17	-	-	-	-	-
MNTW2019 024	Tap	SW	-	0.0001	-	-	-	0.146	-	-	0.146	-	182.0	-	-	-	0.388	-	-	0.133	-	-	-	0.00005	0.148	0.8	0.08	-	-	-	-	-
MNTW2019 025	Tap	SW	-	-	-	-	-	0.143	-	-	0.146	-	84.0	-	-	-	0.150	-	-	0.083	-	-	-	-	0.145	0.6	0.12	-	-	-	-	-
MNTW2019 010	Source	GW	-	-	-	-	-	0.175	-	-	0.054	-	-	-	0.02	0.0009	-	-	-	-	-	-	-	-	0.040	-	0.59	-	-	-	-	-
MNTW2019 009	Finished	GW	-	-	-	-	-	0.175	-	-	0.056	-	16.0	-	-	0.0007	0.015	-	-	0.007	-	-	-	-	0.058	0.8	0.67	-	-	-	-	-
MNTW2019 007	Tap	GW	-	0.0009	-	-	-	0.117	-	0.846	0.062	-	14.0	-	0.01	0.0209	0.063	-	-	0.003	0.001000	-	-	-	0.155	-	0.16	-	-	-	-	-
MNTW2019 008	Tap	GW	-	-	-	-	-	0.152	-	-	0.048	-	20.0	-	-	0.0007	0.252	-	-	0.012	-	-	-	-	0.150	0.6	0.58	-	-	-	-	-
MNTW2019 027	Source	GW	-	-	-	0.0015	0.0097	0.041	-	-	0.020	-	-	-	0.05	-	0.025	-	-	-	-	-	-	-	0.020	-	-	-	-	-	0.00005	0.00013
MNTW2019 026	Finished	GW	-	-	-	0.0013	0.0095	0.036	-	-	0.020	-	10.0	-	-	-	-	-	-	0.017	-	-	-	-	0.033	-	-	-	-	-	0.00009	0.00022
MNTW2019 030	Tap	GW	-	-	-	0.0009	0.0093	0.036	-	-	0.032	-	12.0	-	0.00	-	0.062	-	-	0.023	-	-	-	-	0.020	-	-	-	-	-	-	0.00030
MNTW2019 028	Source	GW	-	-	-	0.0007	0.0108	0.037	0.00001	-	0.042	-	-	-	0.01	-	0.005	-	-	-	-	-	-	-	0.018	-	0.01	-	-	-	0.00012	0.00045
MNTW2019 029	Finished	GW	-	-	-	0.0007	0.0097	0.037	0.00001	-	0.046	-	-	-	0.01	-	0.036	-	-	-	-	-	-	-	0.025	2.7	-	-	-	-	0.00011	0.00045
MNTW2019 031	Tap	GW	-	-	-	0.0006	0.0103	0.037	0.00001	-	0.044	-	2.0	-	-	-	0.072	-	-	0.002	-	-	-	-	0.020	2.9	0.08	-	-	-	0.00012	0.00043
MNTW2019 037	Source	GW	-	-	-	-	-	0.085	-	-	0.064	-	-	-	-	-	0.005	-	-	-	-	-	-	-	0.040	1.3	5.98	0.001200	-	-	-	-
MNTW2019 036	Finished	GW	-	-	-	0.0004	0.0009	0.106	-	-	0.082	-	60.0	-	-	0.0003	0.007	-	-	0.073	0.001000	-	-	-	0.068	0.6	0.03	0.000157	-	-	-	-
MNTW2019 038	Tap	GW	-	-	-	-	-	0.108	-	-	0.082	-	86.0	-	-	-	0.009	-	-	0.093	-	-	-	-	0.163	-	0.27	-	-	-	-	-
MNTW2019 039	Tap	GW	-	0.0001	-	-	-	0.105	-	-	0.080	-	100.0	-	-	-	0.800	-	-	0.093	-	1.25000	-	-	0.150	-	0.08	-	-	-	-	-
MNTW2019 011	Source	SW	-	-	0.0010	-	0.0065	0.044	-	-	0.050	-	-	-	0.02	-	0.004	0.000462	0.000229	-	-	-	0.00003	-	0.020	-	3.82	-	0.00427	0.00002	-	0.00022
MNTW2019 012	Finished	SW	-	-	-	-	-	0.011	-	-	0.038	-	84.0	-	0.01	-	0.002	-	-	0.007	-	-	-	-	0.200	-	-	-	-	-	-	0.00008
MNTW2019 013	Tap	SW	-	-	-	-	-	0.010	-	0.462	0.036	-	66.0	-	0.01	-	0.035	-	-	0.007	-	-	-	0.00004	0.203	1.3	-	-	-	-	-	-
MNTW2019 014	Tap	SW	-	-	-	-	-	0.010	-	-	0.038	-	64.0	-	0.01	-	0.033	-	-	0.005	-	-	-	-	0.198	0.7	-	-	-	-	-	0.00001
MNTW2019 020	Source	GW	-	-	-	-	0.0047	0.173	-	-	0.074	-	-	-	0.01	-	0.028	-	-	-	-	-	-	-	0.033	4.5	0.26	-	-	-	-	0.00015
MNTW2019 019	Finished	GW	-	-	-	-	0.0050	0.186	-	-	0.070	-	62.0	-	0.01	-	0.036	-	-	0.037	-	-	-	-	0.035	8.4	3.54	-	-	-	-	0.00012
MNTW2019 006	Tap	GW	-	-	-	-	0.0042	0.097	-	-	0.074	-	56.0	-	0.01	-	0.144	-	-	0.047	-	-	-	-	0.165	1.6	0.01	-	-	-	-	0.00005
MNTW2019 018	Source	GW	-	-	-	-	0.0060	0.164	-	-	0.052	0.00003	-	-	0.01	-	0.004	-	-	-	-	-	-	-	0.025	-	0.35	-	-	-	-	0.00005
MNTW2019 017	Finished	GW	-	-	-	-	0.0056	0.165	-	-	0.052	-	48.0	-	0.01	-	0.098	-	-	0.042	-	-	-	-	0.163	78.1	0.35	-	-	-	-	0.00006
MNTW2019 021	Tap	GW	-	-	-	-	0.0061	0.061	-	-	0.086	-	84.0	-	0.01	-	0.018	-	-	0.058	-	-	-	0.00005	0.150	-	-	-	-	-	-	0.00007
MNTW2019 002	Source	GW	0.00010	-	-	-	0.0016	0.053	-	-	0.048	-	-	-	0.04	-	0.006	-	-	-	-	-	-	-	0.285	-	0.05	-	-	-	-	-
MNTW2019 005	Tap	GW	-	-	-	-	-	-	-	-	0.058	-	-	-	-	-	0.017	-	-	-	-	-	-	-	0.065	-	-	-	-	-	-	-
MNTW2019 004	Source	SW	-	-	0.0014	-	0.0111	0.031	-	-	0.038	-	-	-	-	-	0.149	-	-	-	-	-	0.00006	-	0.028	-	0.46	-	-	0.00002	-	0.00007
MNTW2019 003	Finished	SW	-	-	-	-	0.0107	0.010	-	-	0.038	-	108.0	-	-	-	0.004	-	-	0.007	-	-	-	0.00003	0.170	11.6	-	-	-	0.00001	0.00003	-
MNTW2019 001	Tap	SW	-	-	0.0008	-	0.0099	0.010	-	-	0.034	-	106.0	-	-	-	0.004	-	-	0.005	-	-	-	-	0.165	3.0	-	-	-	0.00001	-	0.00007
MNTW2019 016	Source	GW	-	-	-	-	-	0.196	-	-	0.056	-	-	-	-	-	0.001	-	-	-	-	-	-	-	0.020	0.6	6.08	-	-	-	-	-
MNTW2019 015	Tap	GW	-	-	-	-	-	0.200	-	-	0.050	-	-	-	-	-	0.002	-	-	-	-	-	-	-	0.018	-	5.77	-	-	-	-	-
MNTW2019 033	Source	GW	-	-	-	0.0005	0.0043	0.050	-	-	0.018	-	-	-	-	-	0.014	-	-	-	-	-	-	-	0.050	0.9	0.43	-	-	-	-	0.00015
MNTW2019 032	Finished	GW	-	-	-	0.0007	0.0048	0.053	-	-	0.020	-	-	6.000000	-	-	0.037	-	-	-	-	-	-	-	1.380	23.2	2.11	-	-	-	-	0.00016
MNTW2019 034	Tap	GW	-	-	-	0.0004	0.0038	-	-	-	0.018	-	28.0	-	-	-	0.353	-	-	0.017	-	-	-	-	0.198	0.9	0.05	-	-	-	-	0.00010
MNTW2019 035	Tap	GW	-	-	-	0.0004	0.0029	0.019	-	-	0.016	-	54.0	-	-	-	0.311	-	-	0.040	-	-	-	0.00003	0.198	2.3	0.09	-	-	-	-	0.00008

Site code	Site Type	Source of Water	Microcystins maxTQ	Molybdenum maxTQ	m-Xylene plus p-xylene maxTQ	Nickel maxTQ	Nitrate maxTQ	OIET maxTQ	o-Xylene maxTQ	PFBS maxTQ	PFNA maxTQ	PFOA maxTQ	PFBA maxTQ	PFHxS maxTQ	PFPeA maxTQ	Prometon maxTQ	Saxatoxins maxTQ	Selenium maxTQ	Simazine maxTQ	Strontium maxTQ	Styrene maxTQ	Sulfamethoxazole maxTQ	Terbuthylazine maxTQ	Tetrachloroethene maxTQ	Tetrachloromethane maxTQ	trans-1,2-Dichloroethene maxTQ	Tribromomethane maxTQ	Trichloroethene maxTQ	Trichloromethane maxTQ	Uranium maxTQ	Zinc maxTQ	ΣTQ
MNTW2019 023	Source	SW	-	-	-	-	0.107	-	-	0.001	-	-	0.0044	0.724	-	0.00001	0.1	-	-	0.060667	-	-	-	0.46552	-	-	-	-	0.01857	-	0.006	1.7
MNTW2019 022	Finished	SW	-	-	-	-	0.037	-	-	-	-	-	0.0022	-	-	-	-	-	-	0.101000	-	-	-	-	-	-	36.0	-	0.03286	-	0.0035	110.9
MNTW2019 024	Tap	SW	-	-	-	-	0.032	-	-	-	-	-	0.0020	-	-	-	-	-	-	0.101667	-	-	-	-	-	-	48.0	-	0.13714	-	0.0085	232.1
MNTW2019 025	Tap	SW	-	-	-	-	0.024	-	-	-	-	-	0.0020	-	-	-	-	-	-	0.101667	-	-	-	-	-	-	34.0	-	0.04000	-	0.0145	119.6
MNTW2019 010	Source	GW	-	0.167	-	-	-	-	-	-	-	1245	0.0023	-	-	-	-	-	-	0.069000	-	-	-	-	-	0.00047	-	1.2	-	-	0.006	1247.4
MNTW2019 009	Finished	GW	-	-	-	-	0.003	-	-	-	-	-	0.0026	-	-	-	-	-	-	0.069667	-	-	-	-	-	0.00038	2.0	0.9	0.01143	50	0.024	70.8
MNTW2019 007	Tap	GW	-	-	-	-	0.004	-	-	-	-	-	0.0016	-	-	-	-	-	-	0.059333	-	-	-	-	-	0.00047	-	0.8	0.01857	-	0.0065	16.3
MNTW2019 008	Tap	GW	-	-	-	-	0.004	-	-	-	-	-	0.0019	-	-	-	-	-	-	0.067000	-	-	-	-	-	0.00028	4.0	0.6	0.01286	-	0.0055	26.5
MNTW2019 027	Source	GW	-	-	-	-	0.803	-	-	0.001	-	2052.5	0.0607	0.397	0.2305	0.00001	-	-	0.00189	0.036333	-	-	-	0.43103	-	-	-	-	0.00143	-	0.055	2054.7
MNTW2019 026	Finished	GW	-	-	-	-	-	-	-	0.002	-	1817.5	0.0455	-	0.1894	0.00002	-	-	-	0.034333	-	-	0.00163	0.39655	-	-	14.0	-	0.00286	-	0.0025	1842.3
MNTW2019 030	Tap	GW	-	-	-	-	0.528	0.000	-	0.002	-	1365	0.0330	-	-	-	-	-	-	0.033200	-	-	-	0.18966	-	-	28.0	1.5	0.00286	-	0.0175	1407.5
MNTW2019 028	Source	GW	-	-	-	-	0.594	0.000	-	-	-	1632.5	0.0198	-	-	0.00001	-	-	-	0.032333	-	0.000105	-	-	-	-	-	3.3	-	-	0.014	1636.6
MNTW2019 029	Finished	GW	-	-	-	-	0.584	0.000	-	-	-	-	0.0233	0.359	-	-	-	-	-	0.032333	-	0.000040	-	-	-	-	-	2.7	-	-	0.0105	6.6
MNTW2019 031	Tap	GW	-	-	-	-	0.519	0.000	-	-	-	1450	0.0222	0.456	-	0.00001	-	-	-	0.032333	-	0.000031	-	-	-	-	2.0	2.9	0.00143	-	0.178	1461.3
MNTW2019 037	Source	GW	-	-	-	-	-	-	-	-	-	-	0.0018	-	-	-	-	-	-	0.074667	-	-	-	-	-	-	-	-	-	-	0.0055	7.6
MNTW2019 036	Finished	GW	-	-	-	0.150	0.013	-	-	-	-	-	0.0022	-	-	-	-	-	-	0.069333	-	-	-	-	-	-	48.0	9.0	0.01571	-	0.064	118.2
MNTW2019 038	Tap	GW	-	-	-	-	0.008	-	-	0.001	-	-	0.0019	-	-	-	-	-	-	0.070667	-	-	-	-	-	-	66.0	9.8	0.04286	-	0.069	162.8
MNTW2019 039	Tap	GW	-	-	-	-	0.007	-	-	-	-	-	0.0021	-	-	-	-	-	-	0.071000	-	-	-	0.18966	-	-	48.0	1.5	0.06286	50	0.062	202.5
MNTW2019 011	Source	SW	1.67	-	-	-	-	0.001	-	-	-	-	0.0015	-	-	-	-	-	-	0.030667	-	-	-	-	-	-	-	0.9	-	40	0.0045	46.6
MNTW2019 012	Finished	SW	-	-	-	-	0.038	-	-	-	-	-	0.0013	-	-	-	-	-	-	0.023333	-	-	-	-	-	-	-	-	0.29714	-	0.002	84.6
MNTW2019 013	Tap	SW	-	-	0.00001	-	0.060	-	-	-	-	-	0.0013	-	-	-	-	-	-	0.025667	-	-	-	-	-	-	-	-	0.24429	-	0.004	68.4
MNTW2019 014	Tap	SW	-	-	-	-	0.062	-	-	-	-	-	0.0011	-	-	-	-	-	-	0.025667	-	-	-	-	-	-	-	-	0.23857	-	0.0045	65.3
MNTW2019 020	Source	GW	-	-	-	-	0.551	-	-	0.001	-	-	0.0018	-	-	-	-	-	-	0.092667	-	-	-	-	-	-	-	-	0.00143	-	0.0075	5.7
MNTW2019 019	Finished	GW	-	-	-	-	0.561	-	-	-	-	-	0.0018	-	-	-	-	-	-	0.092333	-	-	-	-	-	-	12.0	-	0.03286	-	0.013	87.0
MNTW2019 006	Tap	GW	-	-	-	-	0.432	-	-	-	-	-	0.0015	-	-	-	-	-	-	0.093333	-	-	-	-	-	-	18.0	-	0.02571	-	0.069	76.8
MNTW2019 018	Source	GW	-	-	-	0.060	0.465	-	-	-	-	-	0.0021	-	-	-	-	-	-	0.060667	-	-	-	-	-	-	-	-	-	-	0.01	1.2
MNTW2019 017	Finished	GW	-	-	-	-	0.474	-	-	-	-	565.1	0.0017	-	-	-	-	-	-	0.059667	-	-	-	-	-	-	18.0	-	0.01857	40	0.0135	750.7
MNTW2019 021	Tap	GW	-	-	0.00001	-	0.389	-	0.00001	0.001	-	-	0.0015	-	-	-	-	-	-	0.077000	-	-	-	-	-	-	22.0	-	0.04714	-	0.0115	106.9
MNTW2019 002	Source	GW	-	-	-	-	0.345	-	-	0.002	-	1112.5	0.0189	0.433	-	-	-	-	-	0.049333	-	-	-	0.22414	-	-	-	-	-	-	0.0185	1114.1
MNTW2019 005	Tap	GW	-	-	-	-	-	-	-	-	-	632.825	0.0034	-	-	-	-	-	-	-	0.00068	-	-	-	-	-	-	-	-	-	0.0035	633.0
MNTW2019 004	Source	SW	5.00	-	-	-	-	0.001	-	-	-	-	0.0025	-	-	-	-	-	-	0.023333	-	-	-	-	-	-	-	-	-	-	0.0025	5.7
MNTW2019 003	Finished	SW	-	-	-	0.070	0.017	0.001	-	0.001	-	812.5	0.0025	-	-	-	-	-	-	0.023000	-	-	-	-	0.33333	-	-	-	0.41000	-	0.0065	933.2
MNTW2019 001	Tap	SW	-	-	-	-	0.021	0.001	-	0.001	-	952.5	0.0029	-	-	-	-	-	-	0.022667	-	-	-	-	0.33333	-	-	-	0.41143	-	0.0075	1062.5
MNTW2019 016	Source	GW	-	-	-	-	0.003	-	-	-	-	-	0.0221	-	-	-	-	-	-	0.062000	-	-	-	-	-	-	-	-	-	40	0.006	47.0
MNTW2019 015	Tap	GW	-	-	-	-	-	-	-	-	-	-	0.0220	-	-	-	-	-	-	0.063333	-	-	-	-	-	-	-	-	-	40	0.3205	46.4
MNTW2019 033	Source	GW	-	0.233	-	0.040	0.079	0.000	-	0.001	-	1535	0.0350	-	-	-	-	0.100000	-	0.024000	-	-	-	-	-	-	-	-	-	40	0.01	1577.0
MNTW2019 032	Finished	GW	-	-	-	0.160	0.079	0.000	-	0.001	1.181666667	1045	0.030637757	0.295	-	-	-	0.100000	-	0.024000	-	-	-	-	-	-	-	-	-	-	0.555	1080.2
MNTW2019 034	Tap	GW	-	-	-	0.050	0.077	0.000	-	0.001	-	2087.5	0.031986971	0.315	-	-	-	0.100000	-	-	-	-	-	-	-	-	6.0	-	0.06429	-	0.0145	2123.7
MNTW2019 035	Tap	GW	-	-	-	0.070	0.058	0.000	-	0.001	-	-	0.028506086	-	-	-	-	-	-	0.026333	-	-	-	-	-	-	16.0	-	0.03429	60	0.0315	133.2

Table S12. Human-health-based benchmarks in microgram/L (µg/L) used to create toxicity quotient results in Table S11.

[AAP, American Academy of Pediatrics; DWEL, Drinking Water Lifetime Exposure Level; DWHA (OH), Ohio State Drinking Water Health Advisory; HBSL, Health Based Screening Level; HHBP, Human Health Benchmark Pesticide; HRL (MN), Minnesota State Health Risk Level MCL, National Primary Drinking Water Regulation Maximum Contaminant Level; MCL (NY), New York State Maximum Contaminant Level; MCL (MI), Michigan State Maximum Contaminant Level; MCL (NH) New Hampshire Proposed State Maximum Contaminant Level; MCLG, National Primary Drinking Water Regulation Maximum Contaminant Level Goal; MCLG (RL), Reporting Limit (from Table S2) was used in place of an MCLG of zero; Tier 1 PCL (TX), Texas State Tier 1 Protection Concentration Levels; WHO, World Health Organization Drinking Water Guideline. Organoleptic standards not based on human-health concerns; like National Secondary Drinking Water Standards for Al, Cl, Fe, Mn, SO4, Zn; not included. Total Chromium and chromium (IV) have the same WHO Drinking-water standard; only Total Chromium used for TQ analyses. Compounds with an MCLG of 'zero' were set to the laboratory reporting limit (except Pb, which is evaluated against the American Academy of Pediatrics limit of 1.0 ug/L): Trichloroethene, Bromodichloromethane, Tribromomethane, Dichloromethane, Benzene, Tetrachloroethene.]

CAS	Chemical	Value	Type
71-55-6	1,1,1-Trichloroethane	200	MCL
71-55-6	1,1,1-Trichloroethane	1000	DWEL
75-34-3	1,1-Dichloro-2-propanone	80	RAA16 (MN)
75-35-4	1,1-Dichloroethane	20	RAA17 (MN)
94-75-7	2,4-D	70	MCL
171262-17-2	Alachlor oxanilic acid	50	RAA16 (MN)
7440-38-2	Arsenic	0.02	MCLG (RL)
1912-24-9	Atrazine	3	MCLG
7440-39-3	Barium	1300	WHO
25057-89-0	Bentazon	890	HHBP
71-43-2	Benzene	0.026	MCLG (RL)
7440-42-8	Boron	500	RAA17 (MN)
314-40-9	Bromacil	5000	DWEL
75-27-4	Bromodichloromethane	0.05	MCLG (RL)
7440-43-9	Cadmium	0.5	HRL15 (MN)
7440-47-3	Chromium	50	WHO
156-59-2	cis-1,2-Dichloroethene	70	MCLG
7440-50-8	Copper	1300	MCL
35045-02-4	Desamino metribuzin	10	RAA12 (MN)
93413-62-8	Desvenlafaxine	20	HBV15 (MN)
124-48-1	Dibromochloromethane	60	MCLG
75-09-2	Dichloromethane	0.04	MCLG (RL)
87674-68-8	Dimethenamid	40	HBSL
100-41-4	Ethylbenzene	700	MCLG
16984-48-8	Fluoride	4000	MCLG

CAS	Chemical	Value	Type
75-43-4	Dichlorofluoromethane	20	RAA17 (MN)
7439-92-1	Lead	1	AAP
7439-96-5	Manganese	100	HBV18 (MN)
57837-19-1	Metalaxyl	500	HBSL
29385-43-1	Methylbenzotriazole	20	RAA19 (MN)
51218-45-2	Metolachlor	600	HBSL
152019-73-3	Metolachlor oxanilic acid	1000	HVB18 (MN)
171118-09-5	Metolachlor sulfonic acid	1000	HVB18 (MN)
1634-04-4	Methyl tert-butyl ether	700	RAA13 (MN)
7439-98-7	Molybdenum	30	HBSL
179601-23-1	m-Xylene plus p-xylene	10000	MCL
7440-02-0	Nickel	100	HRL93 (MN)
14797-55-8	Nitrate	10000	MCLG
2163-68-0	OIET	60	HHBP
95-47-6	o-Xylene	10000	MCL
375-95-1	Perfluorononanoic acid (PFNA)	0.006	MCL (MI)
1763-23-1	Perfluorooctanesulfonic acid (PFOS)	0.00002	EPA HA
335-67-1	Perfluorooctanoic acid (PFOA)	0.000004	EPA HA
375-73-5	Perfluorobutanesulfonic acid (PFBS)	2	EPA HA
375-22-4	Perfluorobutanoic acid (PFBA)	7	HRL-chronic (MN)
355-46-4	Perfluorohexanesulfonic acid (PFHxS)	0.018	MCL (NH)
2706-90-3	Perfluoropentanoic acid (PFPeA)	0.093	Tier 1 PCL (TX)
1610-18-0	Prometon	500	DWEL
139-40-2	Propazine	700	DWEL
122-34-9	Simazine	4	MCL
7440-24-6	Strontium	3000	RAA19 (MN)
100-42-5	Styrene	100	MCL
723-46-6	Sulfamethoxazole	100	RAA13 (MN)
34014-18-1	Tebuthiuron	900	HBSL
5915-41-3	Terbuthylazine	2	HBSL
127-18-4	Tetrachloroethene	0.058	MCLG (RL)
56-23-5	Tetrachloromethane	0.06	MCLG (RL)
156-60-5	trans-1,2-Dichloroethene	100	MCL
75-25-2	Tribromomethane	0.05	MCLG (RL)
79-01-6	Trichloroethene	0.025	MCLG (RL)
67-66-3	Trichloromethane	70	MCLG
7440-61-1	Uranium	0.1	MCLG (RL)
7440-66-6	Zinc	2000	HRL94
35523-89-8	Saxatoxins	0.3	DWHA (OH)
101043-37-2	Microcystins	0.3	DWHA (OH)
7782-49-2	Selenium	30	HRL93 (MN)
